# Meeting abstracts of the 3° EVIta symposium

**DOI:** 10.20517/evcna.2023.57

**Published:** 2023-12-05

**Authors:** Antonella Bongiovanni, Annalisa Radeghieri, Lorenzo Arnaboldi, Donatella Lucchetti, Giovanna Barbieri, Barile Lucio, Maria Luisa Fiani, Marzia Bedoni, Marco Tripodi, Serena Cavallero, Paolo Bergese, Stefania Biffi, Michela Pozzobon, Carlo Morasso, Benedetta Bussolati, Silvia Monticone, Sandra Buratta, Elena Osto, Alfredo Ambrosone, Pietro Parisse, Valeria Crippa, Stefania Bruno, Roberta Tasso, Saara Laitinen, Simona Fontana, Federica Collino, Igea D’Agnano, Myriam Catalano, Saida Mebarek, Vito G. D’Agostino, Teresa Santantonio, Elisa Panzarini, Claudia Matteucci, Maria Felice Brizzi, Francesco Ferrara, Orazio Fortunato, Alessandro Gori, Marina Cretich, Stefania Raimondo, Ilaria Bellezza, Claudia Martini, Elisabetta Affabris, Fabiola Olivieri, Nunzio Iraci, Giovanna D’Amico, Carolina Balbi, Stefano Papa, Valeria Tarallo, Francesca Fallarino, Daniela Lisini, Fabrizio Bianchi, Alfredo Budillon, Lorenza Lazzari, Valentina Bollati, Ilaria Giusti, Mauro Manno, Massimiliano Ruscica, Alessandro Romano, Valentina Cauda, Gentili Chiara, Daniela Bosisio, Elia Di Schiavi, Dini Luciana, Enrico Lupia, Giuseppe Vassalli, Galimberti Daniela, Paolo Simioni, Roberto Pisano, Vincenzo Denaro, Lorena Urbanelli, Giorgia Melli, Chiara Fenoglio

The 3rd Symposium of the Italian Society of Extracellular Vesicles (The 3° EVIta Symposium), September 13-15, 2023 [Table 1].

**Table 1:** Table of Content

**No**	**Abstract title**	**Authors**	**Pages**
1	Functional enzymatic assays to predict the potency of extracellular vesicles	Giorgia Adamo, Sabrina Picciotto, Angela Paterna, Paola Gargano, Estella Rao, Samuele Raccosta, Monica Salamone, Daniele Paolo Romancino, Mauro Manno, Antonella Bongiovanni	6
2	Exploiting Seahorse Analyzer to evaluate metabolic variations of cells treated with surface functionalized extracellular vesicles	Silvia Alacqua, Miriam Romano, Rossella Zenatelli, Agnese Segala, Maurizio Ragni, Paolo Bergese, Giuseppe Pomarico, Alessandra Valerio, Annalisa Radeghieri	6
3	Size, lipid and protein characterization of different subfractions of extracellular vesicles, with a particular focus on non-membranous exomeres secreted by a melanoma cell line	Felice M. Accattatis, Laura Bianchi, Agnese Granata, Alfonso Carleo, Fabrizio Francomano, Monica Rodolfo, Elisabetta Vergani, Miriam Romano, Paolo Bergese, Marco Brucale, Francesco Valle, Alberto Corsini, Stefano Bellosta, Lorenzo Arnaboldi	7
4	EVs released after chemotherapy treatment of colorectal cancer cells reprogram the cancer-associated fibroblasts	Giulia Artemi, Filomena Colella, Alessandro Sgambato, Donatella Lucchetti	8
5	Extracellular vesicles secreted by MHC class II expressing melanoma cells and their role in immune escape and cancer progression	Francesca Costantini, Caterina Di Sano, Samuele Raccosta, Mauro Manno, Giovanna Barbieri	8
6	Implementation of membrane-sensing-peptide technology in a multiparametric flow cytometry assay: a diagnostic platform for monitoring allograft rejection	Stefano Panella, Ilaria Barison, Jacopo Burrello, Roberto Frigerio, Chiara Castellani, Marny Fedrigo, Elena Provasi, Alessandro Gori, Annalisa Angelini, Marina Cretich, Lucio Barile	9
7	Study of sEV internalization by antigen-presenting cells	Valeria Barreca, Lorenzo Galli, Deborah Polignano, Valentina Tirelli, Massimo Sargiacomo, Maria Luisa Fiani	10
8	Blood derived Extracellular Vesicle as Parkinson’s disease biomarker for the monitoring of neurodegeneration and rehabilitation	Gemma Lombardi, Silvia Picciolini, Alice Gualerzi, Valentina Mangolini, Francesca Rodà, Luana Forleo, Aurora Mangolini, Silvia Ramat, Stefano Giuseppe Doronzio, Diego Longo, Francesca Cecchi, Marzia Bedoni	10
9	miRNA function and partitions are determined by consensus motifs and epitranscriptomic modifications	Sabrina Garbo, Daniel D’Andrea, Francesco Marocco, Gioele Gaboardi, Claudia Montaldo, Carla Cicchini, Cecilia Battistelli, Marco Tripodi	11
10	Comparative transcriptomic analyses on human intestinal organoids exposed to Anisakis-derived extracellular vesicles	Ilaria Bellini, Daniela Scribano, Cecilia Ambrosi, Claudia Chiovoloni, Silvia Rondon, Annamaria Pronio, Anna Teresa Palamara, Stefano D’Amelio, Serena Cavallero	12
11	Interaction and nanoplasmonics of lipoproteins and gold nanoparticles	Andrea Zendrini, Jacopo Cardellini, Roberto Frigerio, Debora Berti, Marina Cretich, Paolo Bergese	13
12	Syndecan-Syntenin-Alix complex mediates SRC function in macrophages’ small-EV release.	Vanessa Biemmi, Stefano Panella, Jacopo Burrello, Edoardo Lazzarini, Yulia Goshovska, Giuseppina Milano, Lucio Barile	14
13	Extracellular vesicles as a potential source of biomarkers in endometriosis	Barbara Bortot, Alessandro Mangogna, Alice Gualerzi, Silvia Picciolini, Giovanni Di Lorenzo, Federico Romano, Gabriella Zito, Ben Peacock, Rebecca Lees, Giuseppe Ricci, Stefania Biffi	14
14	*in vitro* models to study the anti-fibrotic and anti-oxidative mechanism of extracellular vesicles derived from umbilical cord mesenchymal stromal cells	Paola Bisaccia, Fabio Magarotto, Marcin Jurga, Maurizio Muraca, Michela Pozzobon	15
15	Raman spectroscopic analysis of lipoproteins quality in obese and healthy subjects	Arianna Bonizzi, Flavia Magri, Serena Mazzucchelli, Marta Truffi, Andrea Rizzi, Fabio Corsi, Roberta Cazzola, Carlo Morasso	16
16	Engineered red blood cell extracellular vesicles as a therapeutic strategy for the treatment of renal diseases	Alessia Brossa, Alessandro Gori, Marina Cretich, Cristina Grange, Ilaria Giusti, Vincenza Dolo, Malvina Koni, Maria Felice Brizzi, Benedetta Bussolati	16
17	Small-RNA sequencing of human urinary extracellular vesicles reveals the renal pro-inflammatory role of a high-sodium diet	Fabrizio Buffolo, Jacopo Burrello, Barbara Pardini, Giuseppe Matullo, Giovanni Birolo, Margherita Alba Carlotta Pomatto, Paolo Mulatero, Brooke Honzel, Yvonne Niebuhr, Anand Vaidya, Silvia Monticone	17
18	Agri-food byproducts: from waste to resource for isolation of NanoVesicles	Raffaella Latella, Lorena Urbanelli, Elisabetta Chiaradia, Anna Maria Salzano, Alessia Tognoloni, Roberto Maria Pellegrino, Husam Alabed, Giada Cerotti, Brunella Tancini, Andrea Scaloni, Carla Emiliani, Sandra Buratta	18
19	Cardiovascular risk, metabolic profile and Inflammatory fingerprint of obese subjects who underwent bariatric surgery	Jacopo Burrello, Mary Julieth Gonzalez, Lorenzo Airale, Alessio Burrello, Thomas Köstler, Urs Zingg, Lucio Barile, Elena Osto	19
20	Extracellular vesicles from plant biotechnology platforms: potential applications for human health	Elisa Cappetta, Mariapia Vietri, Marisa Conte, Valentina Santoro, Nunziatina De Tommasi, Fabrizio Dal Piaz, Mariaevelina Alfieri, Antonietta Leone, Alfredo Ambrosone	20
21	Small extracellular vesicles in triple-negative breast cancer models	Loredana Casalis, Beatrice Senigagliesi, Pietro Parisse	20
22	The impairment of the protein quality control system affects extracellular vesicle miRNA secretion in cellular models of TDP-43 proteinopathies	Elena Casarotto, Daisy Sproviero, Letizia Messa, Maria Garofalo, Stephana Carelli, Marta Cozzi, Marta Chierichetti, Riccardo Cristofani, Veronica Ferrari, Mariarita Galbiati, Margherita Piccolella, Paola Rusmini, Barbara Tedesco, Paola Pramaggiore, Laura Cornaggia, Guglielmo Patelli, Cristina Cereda, Stella Gagliardi, Angelo Poletti, Valeria Crippa	21
23	Extracellular vesicles derived from human liver stem cells counteract chronic kidney disease development and cardiac dysfunction in remnant kidney murine model	Elena Ceccotti, Massimo Cedrino, Giulia Chiabotto, Cristina Grange, Alessandra Ghigo, Alessandro Gambella, Maria Felice Brizzi, Giovanni Camussi, Stefania Bruno	22
24	Targeting of the tumor extracellular matrix by click chemistry-based surface engineering of extracellular vesicles (EVs)	Maria Chiara Ciferri, Nicole Rosenwassen, Enrico Millo, Daniele Reverberi, Silvia Bruno, Cansu Gorgun, Rodolfo Quarto, Roberta Tasso	23
25	Red Blood Cells-derived Extracellular Vesicles as targetable drug delivery vehicles	Maria Chiara Ciferri, Kai Härkönen, Juha Prittinen, Ulla Impola, Petra Ilvonen, Henri Tuovinen, Tapani Viitala, Enrico Millo, Roberta Tasso, Saara Laitinen	23
26	Developing liver spheroids as a model to investigate the role of colorectal cancer-derived small extracellular vesicles in metastatic niche establishment	Elisa Costanzo, Ornella Urzi’, Marzia Pucci, Marta Moschetti, Alice Conigliaro, Marco Loria, Maria Cristina Guerrera, Riccardo Alessandro, Simona Fontana	24
27	Urinary Extracellular Vesicles Fatty Acid Profiling captures lipid metabolism changes in childhood Steroid-Sensitive Nephrotic Syndrome	Giulia Cricri, Stefano Turolo, Linda Bellucci, Chiara Tamburello, Irene Paraboschi, Gianantonio Manzoni, William Morello, Giovanni Montini, Federica Collino	25
28	Small extracellular vesicle miRNA cargo profiling in human glioblastoma preclinical *in vivo* models as candidate biomarkers for liquid biopsy	Laura Vilardo, Ingrid Cifola, Giuliana Gatti, Nicolò Panini, Alessio Torcinari, Fabrizio Bonaventura, Tiziana Orsini, Francesca De Santa, Myriam Catalano, Marcello Raspa, Igea D’Agnano	26
29	Astrocytes-Derived Small Extracellular Vesicles Hinder Glioma Growth	Mariassunta De Luca, Carmela Serpe, Lucia Monaco, Arianna Rinaldi, Igea D’Agnano, Cristina Limatola, Myriam Catalano	26
30	The role of soluble proteins in the biological functions of matrix vesicles	Maryanne Trafani de Melo, Lucas Fabricio Bahia Nogueira, Pietro Ciancaglini, José Luis Millán, Massimo Bottini, Ana Paula Ramos, Saida Mebarek	27
31	Prognostic potential of urinary extracellular vesicle-microRNAs for prostate cancer patients	Giuseppe De Palma, Eleonora Torchia, Michela Notarangelo, Vito F. Di Lorenzo, Antonio Tufaro, Alessandro Mastrorosa, Vito G. D’Agostino	27
32	Interferon-gamma downregulates CD44 on extracellular vesicles via STAT1 in A549 lung cancer cells	Dian J. Salih, Katrin S Reiners, Zulema Antonia Percario, Loizzi Domenico, Sollitto Francesco, Martin Schlee, Elisabetta Affabris, Gunther Hartmann, Teresa Santantonio	28
33	Fruit-derived extracellular vesicle effects on a gastrointestinal barrier model of intestinal inflammation	Di Giulio Simona, Mariano Stefania, Carata Elisabetta, Elisa Panzarini	29
34	Dynamic expression of CD169 in blood cells and circulating microvesicles as a potential marker in the development and progression of COVID-19 and post-acute sequelae	Marialaura Fanelli, Vita Petrone, Rossella Chirico, Christian Maracchioni, Martina Giudice, Elisabetta Teti, Luigi Coppola, Chiara Sorace, Marco Iannetta, Marta Zordan, Pietro Vitale, Loredana Sarmati, Alexandre Lucas, Emanuela Balestrieri, Sandro Grelli, Claudia Maria Radu, Antonella Minutolo, Claudia Matteucci	29
35	Circulating EV from unstable angina patients as a novel diagnostic marker to stratify different patient populations	Saveria Femminò, Alessandro Sarcinella, Alberto Grosso, Stefania Bruno, Ovidio De Filippo, Fabrizio D’Ascenzo, Maria Felice Brizzi	30
36	On-chip device for flow-driven release of extracellular vesicles	Alessia Foscarini, Valeria Garzarelli, Antonio Turco, Annamaria Nigro, Maria Serena Chiriacò, Elisabetta Primiceri, Alessandro Romano, Francesco Ferrara	31
37	Plasma extracellular vesicles promote lung cancer pre-metastatic niche formation through endothelial modulation	Francesca Pontis, Ilaria Petraroia, Patrizia Ghidotti, Mattia Boeri, Sabina Sangaletti, Paola Suatoni, Ugo Pastorino, Fabio Maiullari, Roberto Rizzi, Claudia Bearzi, Gabriella Sozzi, Orazio Fortunato	32
38	Integrated diagnostic workflow for blood and urinary Extracellular Vesicles by Membrane Sensing Peptides and digital detection	Marina Cretich, Roberto Frigerio, Paola Gagni, Giulia Lodigiani, Stefano Panella, Jacopo Burrello, Adele Tanzi, Cristina Grange, Lucio Barile, Benedetta Bussolati, Alessandro Gori	32
39	Pan-specific, affinity isolation of small Extracellular Vesicles from minimally pretreated biological fluids by Membrane Sensing Peptides	Alessandro Gori, Roberto Frigerio, Paola Gagni, Giulia Lodigiani, Stefano Panella, Elena Provasi, Adele Tanzi, Cristina Grange, Lucio Barile, Benedetta Bussolati, Marina Cretich	33
40	Sorting of single lipoproteins from plasma and serum samples	Roberto Frigerio, Alessandro Gori, Paolo Bergese, Marina Cretich	34
41	Engineered small extracellular vesicles for biogenesis and immunomodulation studies	Lorenzo Galli, Deborah Polignano, Valeria Barreca, Roberta Bona, Valentina Tirelli, Massimo Sargiacomo, Maria Luisa Fiani	35
42	Antioxidant effect of nanovesicles derived from lemon juice on hepatocytes	Roberta Gasparro, Giulia Duca, Vincenza Tinnirello, Nima Rabienezhad Ganji, Simona Fontana, Riccardo Alessandro, Stefania Raimondo	35
43	Potential involvement of urinary and hepatocyte-derived extracellular vesicles in glyoxylate detoxification.	Leonardo Gatticchi, Rita Romani, Barbara Cellini, Ilaria Bellezza	35
44	Effects of Extracellular Vesicles derived from human microglia cells on glioblastoma tumor microenvironment	Lorenzo Germelli, Lorenzo, Ceccarelli, Laura Marchetti, Milena Rizzo, Aldo Moscardini, Miriam Romano, Chiara Giacomelli, Paolo Bergese, Claudia Martini	36
45	Immunoregulatory role of Extracellular Vesicles in advanced Non-Small Cell Lung Cancer	Patrizia Ghidotti, Diego Signorelli, Claudia Proto, Marta Brambilla, Francesca Pontis, Ilaria Petraroia, Benedetta Bussolati, Cristina Grange, Gabriella Sozzi, Elena Jachetti, Orazio Fortunato	37
46	The influence of interferons on extracellular vesicles produced by primary monocyte-derived macrophages	Flavia Giannessi, Valentina Lombardi, Zulema A. Percario, Andrea Sabatini, Alessandra Sacchi, Luca Battistini, Giovanna Borsellino, Daniela F. Angelini, Elisabetta Affabris	38
47	DNA: RNA hybrids accumulate in the cytoplasm of senescent endothelial cells and are released through extracellular vesicles	Angelica Giuliani, Deborah Ramini, Jacopo Sabbatinelli, Michele Guescini, Gianluca Storci, Spartaco Santi, Massimiliano Bonafè, Fabiola Olivieri	38
48	Characterization of urinary EVs isolated with different purification methods using single EV techniques	Cristina Grange, Diego Prudente, Benedetta Bussolati	39
49	Brain region specificity of astrocyte-derived extracellular vesicles: uncovering the mechanisms of neuroprotection in Parkinson’s disease	Loredana Leggio, Fabrizio Cavallaro, Greta Paternò, Maria Gaetana Giovanna Pittalà, Sharon N. Cox, Marco Falcone, Marco Catania, Mauro Distefano, Ernesto Picardi Rosaria Saletti, Nunzio Iraci	40
50	Activin A modulates the microRNA cargo of extracellular vesicles released by B-Cell Precursor Acute Lymphoblastic Leukemia (BCP-ALL) cells	Eugenia Licari, Giulia Cricrì, Francesca Raimondo, Marina Pitto, Silvia Bresolin, Andrea Biondi, Valentina Bollati, Erica Dander, Giovanna D'Amico	41
51	Exercise-stimulated extracellular vesicles promote cardiomyocyte pro-survival programming in a redox-dependent manner.	Veronica Lisi, Giorgia Senesi, Nadia Bertola, Matteo Pecoraro, Sara Bolis, Alice Gualerzi, Silvia Picciolini, Andrea Raimondi, Cristina Fantini, Elisa Moretti, Attilio Parisi, Paolo Sgrò, Luigi Di Luigi, Roger Geiger, Silvia Ravera, Giuseppe Vassalli, Daniela Caporossi, Carolina Balbi	41
52	C. jejuni CDT intoxicated-Caco-2 cells release EVs that inhibit proliferation in tumor intestinal epithelial and myeloid cells: potential utility for antitumor strategies.	Daniele Lopez, Barbara Canonico, Mariele Montanari, Michele Guescini, Raffaella Campana, Giovanna Panza, Caterina Ciacci, Francesca Luchetti, Claudio Ortolani, Stefano Papa	42
53	Cancer-derived exosomal-Alu RNA promotes colorectal cancer progression by inducing epithelial-to-mesenchymal transition through NLRP3 inflammasome activation.	Sara Magliacane Trotta, Antonio Adinolfi, Sandro De Falco, Valeria Tarallo	43
54	Amniotic fluid stem cell-derived extracellular vesicles reprogram type 2 conventional dendritic cells in experimental autoimmune encephalomyelitis	Giorgia Manni, Marco Gargaro, Simona Fontana, Marco Cipolloni, Tommaso Mazza, Doriana Ricciuti, Alessandro di Michele, Giulia Mencarelli, Benedetta Pieroni, Francesco Sarnari, Alessandra di Veroli, Rita Romani, Francesca Fallarino	43
55	Extracellular Vesicles from Adipose Tissue-derived Mesenchymal Stromal Cells loaded with Paclitaxel: isolation and characterization for future clinical use as an antitumor drug	Angela Marcianti, Eleonora Spampinato, Sara Nava, Simona Frigerio, Simona Pogliani, Giulia Maria Stella, Catia Traversari, Paola Gagni, Federico Cazzaniga, Angelo Guido Corsico, Daniela Lisini	44
56	Dissecting exosomal miRNAs as key players for the early identification of an aggressive subtype of lung adenocarcinoma	Francesco Mazzarelli, Elisa Dama, Roberto Cuttano ,Rosa Maria Perrone, Patricia Kiptiu, Valentina Melocchi, Kuku Miriam Afanga, Tommaso Colangelo, Fabrizio Bianchi	45
57	A novel prognostic biomarker signature reflected by Large Oncosomes is associated with aggressive Prostate Adenocarcinoma	Rossella Migliorino, Chiara Ciardiello, Domenico Mallardo, Maria S. Roca, Rita Lombardi, Vincenzo Gigantino, Giosuè Scognamiglio, Carlo Vitagliano, Biagio Pucci, Tania Moccia, Francesca Bruzzese, Susan Costantini, Marinella Pirozzi, Maria Mangini, Deniz Yilmaz, Anna Chiara De Luca, Michele Minopoli, Paolo Antonio Ascierto, Alessandra Leone, Elena Di Gennaro, Alfredo Budillon	46
58	Involvement of the extracellular vesicles/macrophages/neurons axis in Amyotrophic Lateral Sclerosis	Elisabetta Carata, Marco Muci, Stefania Mariano, Elisa Panzarini	47
59	Surface functionalization of EVs with antibodies and the protein corona ‘’variable’’	Angelo Musicò, Rossella Zenatelli, Miriam Romano, Andrea Zendrini, Silvia Alacqua, Selene Tassoni, Lucia Paolini, Chiara Urbinati, Marco Rusnati, Paolo Bergese, Giuseppe Pomarico, Annalisa Radeghieri	47
60	Comparing EVs from solid and fluid human tissues: do’s and don’ts.	Paolini Lucia, Mangolini Valentina, Simone Piva, Cattaneo Stefano, Brucale Marco, Valle Francesco, Montis Costanza, Federici Stefania, Gazzina Stefano, Guarneri Bruno, Radeghieri Annalisa, Latronico Nicola, Paolo Bergese	48
61	Small extracellular vesicles from human pluripotent stem cells: properties, miRNA/circRNA landscape and functions	Valeria Peli, Mario Barilani, Clelia Pistoni, Francesco Rusconi, Eva Maria Pinatel, Francesca Pischiutta, Alessandro Cherubini, Dorian Tace, Maria Chiara Iachini, Vincenza Dolo, Giovanna Damia, Roberta Roncarati, Beatrice Fontana, Ilaria Pace, Manuela Ferracin, Elisa R Zanier, Lorenza Lazzari	49
62	Effect of environmental and lifestyle factors on circulating oncomiRs carried by extracellular vesicles in a population of subjects with overweight or obesity	Paola Monti, Chiara Favero, Luca Ferrari, Laura Dioni, Francesca Bianchi, Angela Cecilia Pesatori, Elia Biganzoli, Valentina Bollati	50
63	Effects of intracellular pathway inhibitors on the secretion, protein, and lipid composition of fluorescent Bodipy sEV	Deborah Polignano, Valeria Barreca, Lorenzo Galli, Francesco Bonanni, Valentina Tirelli, Massimo Sargiacomo, Maria Luisa Fiani	51
64	Evaluation of extracellular vesicle-associated TGF-β as a biomarker in patients with endometriosis	Giuseppina Poppa, Giulia Di Fazio, Giulia Capanna, Maurizio Guido, Vincenza Dolo, Ilaria Giusti	51
65	Extrusion-based biotechnology for EV exogenous loading	Estella Rao, Angela Paterna, Giorgia Adamo, Sabrina Picciotto, Pamela Santonicola, Valeria Longo, Noemi Aloi, Paola Gargano, Daniele Romancino, Rita Carrotta, Samuele Raccosta, Paolo Colombo, Elia Di Schiavi, Antonella Bongiovanni, Mauro Manno	52
66	Characterization of extracellular vesicles in cardiomyopathies	Alessandra Stefania Rizzuto, Maria Francesca Greco, Andrea Faggiano, Marco Vicenzi, Stefano Carugo, Chiara Macchi, Massimiliano Ruscica	53
67	TDP-43 is a physiological EV cargo and its release is impaired in ALS pathology.	Annamaria Nigro, Roberto Furlan, Nilo Riva, Angelo Quattrini, Alessandro Romano	54
68	Fully artificial extracellular vesicles: a biomimicking strategy towards effective theranostic tools in nanomedicine.	Giada Rosso, Valentina Cauda	54
69	Spheroids culture affects cellular senescence and increases the angiogenic potential of Mesenchymal Stromal Cells (MSCs)-derived secretome and extracellular vesicles (EVs)	Matteo rovere, Daniele Reverberi, Maria Elisabetta Palmalà, Chiara Gentili	55
70	Small extracellular vesicles and interferons in U-373 astrocytoma cells line	Melissa Gionfriddo, Flavia Giannessi, Andrea Sabatini, Alessandra Sacchi, Zulema Antonia Percario, Luca Battistini, Giovanna Borsellino, Daniela F. Angelini, Elisabetta Affabris	55
71	Exosomes released by UV-treated keratinocytes activate pDCs via TLR7: a model mechanism of type I interferon triggering in psoriasis	Valentina Salvi, Carolina Gaudenzi, Silvia Alacqua, Silvano Sozzani, Paolo Bergese, Daniela Bosisio	56
72	C. elegans as an *in vivo* model to characterize EV bioactivity and biodistribution	Pamela Santonicola, Rosa Mocerino, Giorgia Adamo, Sabrina Picciotto, Andrea Zendrini, Daniele P. Romancino, Angela Paterna, Estella Rao, Samuele Raccosta, Stella Frabetti, Olivia Candini, Nicolas Touzet, Mauro Manno, Annalisa Radeghieri, Miriam Romano, Paolo Bergese, Antonella Bongiovanni, Elia Di Schiavi	56
73	Extracellular vesicles from plasma and skeletal muscle of Amyotrophic Lateral Sclerosis mice models induce differential metabolic alterations in recipient myotubes	Carolina Sbarigia, Stefano Tacconi, Simone Dinarelli, Silvia Scaricamazza, Cristiana Valle, Luciana Dini	57
74	Extracellular vesicles isolated from human plasma as a potential diagnostic and prognostic tool in burn septic shock patients	Martina Schiavello, Barbara Vizio, Filippo Mariano, Stefania Bruno, Ornella Bosco, Anna Pensa, Daniela Risso, Giovanni Camussi, Giuseppe Montrucchio, Enrico Lupia	58
75	Human cardiomyocyte-derived extracellular vesicles regulate cardiac fibroblast activation through miR-24	Giorgia Senesi, Alessandra M. Lodrini, Davide Ceresa, Sara Bolis, Paolo Malatesta, Marie-José Goumans, Carolina Balbi, Giuseppe Vassalli	59
76	Neural-Derived Extracellular Vesicles and their non-coding RNAs (ncRNAs) cargo in Frontotemporal Dementia and Bipolar Disorder: an epigenetic approach useful for a differential diagnosis	Maria Serpente, Chiara Fenoglio, Andrea Arighi, Emanuela Rotondo, Caterina Visconte, Marina Arcaro, Giorgio Bocca, Giuseppe Delvecchio, Lorena Di Consoli, Adele Ferro, Cecilia Prunas, Antonio Callari, Paolo Brambilla, Daniela Galimberti D	60
77	MicroRNAs loaded in circulating small neural-derived extracellular vesicles: potential biomarkers in early diagnosis of Alzheimer's disease	Tatiana Spadoni, Angelica Giuliani, Marica Pagliarini, Michele Guescini, Patrizia Ambrogini, Anna Rita Bonfigli, Giuseppe Pelliccioni, Maria Cristina Albertini, Laura Graciotti, Fabiola Olivieri	60
78	Study of isolated plasma extracellular vesicles bearing Sars-CoV-2 nucleoprotein of COVID-19 patients and their roles in the disease progression	Serena Toffanin, Giorgia Nuozzi, Claudia Maria Radu, Elena Campello, Cristiana Bulato, Paolo Simioni	61
79	From bench to bed translation of extracellular vesicles (EVs)-based pharmaceuticals: a comparative study of different preservation methods	Francesca Susa, Tania Limongi, Francesca Borgione, Silvia Peiretti, Marta Vallino, Valentina Cauda, Roberto Pisano	62
80	Looking for potential acute kidney injury biomarkers on the urinary extracellular vesicle surface	Adele Tanzi, Sarah Tassinari, Cristina Grange, Valentina Bettio, Daniela Capello, Vincenzo Cantaluppi, Benedetta Bussolati	62
81	Colorectal cancer EV ID-CARD: surface profile of vesicles extracted from different human CRC tissues.	Sarah Tassinari, Edoardo D’Angelo, Federico Caicci, Jacopo Burrello, Alessandro Musso, Giuseppe Giraudo, Marco Ettore Allaix, Giorgio Maria Saracco, Mario Morino, Paola Cassoni, Marco Agostini, Federica Collino, Benedetta Bussolati	63
82	Wharton's Jelly MSCs derived extracellular vesicles as a cell-free therapeutic approach for intervertebral disc degeneration	Veronica Tilotta, Giuseppina Di Giacomo, Claudia Cicione, Luca Ambrosio, Fabrizio Russo, Rocco Papalia, Gianluca Vadalà, Vincenzo Denaro	64
83	Autophagy beyond degradation: impairment of autophagic flux results in the release of extracellular vesicles carrying autophagy-associated markers	Giada Cerrotti, Sandra Buratta, Raffaella Latella, Roberto Maria Pellegrino Husam B.R. Alabed, Brunella Tancini, Paolo Gorello, Francesco Arcioni, Carla Emiliani, Lorena Urbanelli	65
84	Differential regulation of PBMCs by plasma-derived extracellular vesicles from Parkinson’s disease patients	Elena Vacchi, Stefano Panella, Carolina Balbi, Lucio Barile, Alain Kaelin-Lang, Giorgia Melli	65
85	The role of extracellular vesicles in the modulation of temozolomide-resistant and sensitive glioblastoma cells	Diana Vardanyan, Iulia Efimova, Robin Demuynck, Stefano Tacconi, Dmitri Krysko, Luciana Dini	66
86	Use of Extracellular Vesicles expressing SARS-CoV-2 Spike protein (S-EVs) as a model of virus-like particles for possible theragnostic applications	Roberta Verta, Cristina Grange, Sarah Tassinari, Benedetta Bussolati	67
87	Brain-derived Extracellular Vesicles in dementia	Caterina Visconte, Maria Serpente, Maria Teresa Golia, Claudia Verderio, Martina Gabrielli, Federica Sorrentino, Marina Arcaro, Andrea Arighi, Beatrice Arosio, Elio Scarpini, Daniela Galimberti, Chiara Fenoglio	68
88	Functional effects of plasma-derived extracellular vesicles of patients with Parkinson`s disease *in vitro* can reveal novel pathways of neurodegeneration	Ankush Yadav, Elena Vacchi, Sandra Pinton, Alain Kaelin-Lang, Giorgia Melli	69

## 1. Functional enzymatic assays to predict the potency of extracellular vesicles

Giorgia Adamo^1^, Sabrina Picciotto^1^, Angela Paterna^2^, Paola Gargano^1^, Estella Rao^2^, Samuele Raccosta^2^, Monica Salamone^1^, Daniele Paolo Romancino^1^, Mauro Manno^2^, Antonella Bongiovanni^1^

^1^Cell-Tech HUB and Institute for Research and Biomedical Innovation, National Research Council of Italy (CNR), Palermo, Italy.

^2^Cell-Tech HUB and Institute of Biophysics (IBF) - CNR, Via Ugo La Malfa 153, Palermo, Italy.

Extracellular vesicles (EVs) are crucial signaling mediators involved in intercellular and inter-organism communication, with potential use in cell-free therapies. Despite the guidelines established by MISEV2018 for the harmonization of EVs, standardization of functional or potency assays for EVs has become a crucial issue. Many *in vitro* and *in vivo* assays are used to determine EV bioactivity, but they are not applicable as universal assays. Developing reliable functional assays for EVs can be challenging due to their heterogeneity and different bioactivities. However, specific quantitative assays are required to validate EV preparations for subsequent clinical applications. In this study, a new, simple, universal, and highly sensitive enzymatic-based functional assay was developed to predict EV potency by evaluating the bioactivity and the membrane integrity in a single-step analysis. The assay was validated using nanoalgosomes (e.g., microalgae-derived extracellular vesicles) as a model system and explored for EV-quality check after different isolation, storage, and loading methods. The proposed functional enzymatic assay is quantitative, cost-effective, fast, and reliable, with the potential for numerous applications, including quality control assessment of EV potency.

## 2. Exploiting seahorse analyzer to evaluate metabolic variations of cells treated with surface functionalized extracellular vesicles

Silvia Alacqua^1,2^, Miriam Romano^1,2^, Rossella Zenatelli^1,2^, Agnese Segala^1^, Maurizio Ragni^3^, Paolo Bergese^1,2,4,5^, Giuseppe Pomarico^1,2^, Alessandra Valerio^1^, Annalisa Radeghieri^1,2^

^1^Dept. of Molecular and Translational Medicine, University of Brescia, Brescia, Italy.

^2^Center for Colloid and Surface Science (CSGI), Florence, Italy.

^3^Center for Study and Research on Obesity, Department of Medical Biotechnology and Translational Medicine, University of Milan, Milan, Italy.

^4^Institute for Research and Biomedical Innovation (IRIB), National Research Council, Palermo, Italy.

^5^National Inter-university Consortium of Materials Science and Technology (INSTM), Firenze, Italy.

Measurement of cellular bioenergetics is an increasingly applied technique employing label-free sensors to analyze the major pathways for energy production: mitochondrial respiration and glycolysis. The Seahorse Extracellular Flux Analyzer can detect real-time changes in oxygen consumption and proton efflux through oxygen consumption rate (OCR) and extracellular acidification rate (ECAR) parameters, providing information on live cell metabolism. Surprisingly, this technology has been scarcely employed to investigate metabolic effects induced by Extracellular Vesicle (EV) uptake in cultured cells. In our contribution, we will present explorative results on applying Seahorse XFe24 Analyzer (Agilent) for studying metabolic variations of cells treated with Red Blood Cell-derived EVs (RBC-EVs), functionalized or not with Cetuximab (CTX). Breast cancer EGFR-positive MDA-MB-231 cells were treated with MISEV 2018 compliant RBC-EVs, native and covalently functionalized by bioorthogonal method with recombinant monoclonal antibody CTX. This antibody is a well-known ligand for EGF receptor (EGFR) and a widely used antitumor drug. To evaluate the metabolic effect of functionalized RBC-EVs, we performed the Cell Mito Stress Test (Agilent), testing RBC-EVs and CTX, alone and in combination. Preliminary results generally showed higher OCR for treated cells compared to untreated cells due to an increase in mitochondrial activity, and greater effects for EVs functionalized with CTX, suggesting a higher specificity and efficacy in targeting cells. Seahorse analysis requires only a small cell number, provided they are evenly seeded, and seems to be more sensitive and informative in showing metabolic variations than common viability assays.

## 3. Size, lipid and protein characterization of different subfractions of extracellular vesicles, with a particular focus on non-membranous exomeres secreted by a melanoma cell line

Felice M. Accattatis^1^, Laura Bianchi^2^, Agnese Granata^3^, Alfonso Carleo^4^, Fabrizio Francomano^2^, Monica Rodolfo^5^, Elisabetta Vergani^5^, Miriam Romano^6,8^, Paolo Bergese^6,8^, Marco Brucale^7,8^, Francesco Valle^7,8^, Alberto Corsini^3^, Stefano Bellosta^3^, Lorenzo Arnaboldi^3^

^1^DFFSN, UNICAL, Rende, Italy.

^2^Dept. of Life Sciences, UNISI, Siena, Italy.

^3^DISFeB “Rodolfo Paoletti”, UNIMI, Milan, Italy.

^4^Fraunhofer Institute Hannover, Hannover, Germany.

^5^Fondazione IRCCS INT, Milan, Italy.

^6^DMMT, UNIBS, Brescia, Italy.

^7^CNR Bologna, Bologna, Italy.

^8^CSGI-Italian Center for Colloid and Interface Science, Firenze, Italy

Extracellular vesicles (EVs) participate in pathophysiological processes by transferring their cargo among cells, but unproper separation and characterization methods make it difficult to understand their functions. To overcome this problem, we optimized an ultracentrifugation method to size-separate different EV populations *in vitro*, following an algorithm developed by Livshits. After separation, we characterized five subfractions secreted by a melanoma cell line by transmission electron microscopy (TEM), atomic force microscopy (AFM), and dynamic light scattering (DLS). Fatty acid (FA) profiles were evaluated by GLC, while cargo proteins were analyzed by functional proteomics, western blot, and CONAN. AFM, TEM, and DLS confirmed the EV-subfractions theoretical sizes calculated by the algorithm. The continuous % increase in saturated FA ranging from parental cells to smallest-sized exomeres (33.61%-64.79%) and AFM data suggest different membrane rigidity and properties among populations. Mass spectrometry analysis identified 2003 proteins with qualitative/quantitative differential distribution among populations, with no significant presence of exogenous proteins (CONAN). The MetaCore pathway analysis performed on individual cargos evidenced common signaling pathways but also specific molecular properties characteristic of each fraction, suggesting distinctive behaviors and functions. MetaCore and western blot analysis revealed that prooncogenic proteins (MMP-9, TIF1-beta) are exclusively present in the exomeres, the most interesting particles. The interactomic analysis performed on exomeres also evidenced a complex and highly integrated network aimed at finely regulating target cell and environmental plasticity. We are planning to perform functional assays and to pharmacologically modify these fractions to determine the clinical relevance of these findings. Supported by EXTRALIPO Bando SEED-2019.

## 4. Evs released after chemotherapy treatment of colorectal cancer cells reprogram the cancer-associated fibroblasts

Giulia Artemi^1^, Filomena Colella^2^, Alessandro Sgambato^1,2^, Donatella Lucchetti^1,2^

^1^Dipartimento di Medicina e Chirurgia Traslazionale, Università Cattolica del Sacro Cuore, Rome, Italy.

^2^Fondazione Policlinico Universitario Agostino Gemelli IRCCS, Rome, Italy.

Despite the advancement of cancer therapies, colorectal cancer remains one of the leading causes of death worldwide. Chemotherapy is the main treatment for these patients, even if a great effort is needed to increase the efficacy of therapies and to improve patient survival. A better understanding of crosstalk between cancer cells and tumor microenvironment during the administration of chemotherapy is crucial to developing more effective therapeutic approaches. Extracellular vesicles (EVs) play a crucial role in intercellular communication and can induce a metabolic switch that occurs in cancer and tumor-stroma cells, supporting tumor cell growth. Increasing evidence suggests that cancer-associated fibroblast (CAF) can secrete metabolites to fuel tumor cell growth, allowing the Reverse Warburg Effect. The aim of this study was to analyze the effects of EVs isolated from HT29 cells treated with 5-fuoracil and oxaliplatin (5-Fu and Oxa) on CAFs metabolic reprogramming. Our data demonstrated an increase in the number of EVs released from HT29-treated 5-Fu and Oxa (EVs-5-Fu and EVs-Oxa) compared to EVs released by untreated cells (EVs-CTR), suggesting a putative increase in their biogenesis. Moreover, we showed an increase in proteins involved in glycolysis (as PKM2) and cancer progression (such as CD147, VEGF, and β-catenin) in EVs-5-Fu or EVs-Oxa compared to EVs-CTR. Finally, CAFs treated with EVs-5-Fu or EVs-Oxa showed an increased expression of Vimentin and α-SMA, markers of myofibroblast differentiation. In conclusion, our preliminary data showed that EVs released by the CRC cell line, subjected to chemotherapeutic treatments, could induce a reprogramming of CAFs.

## 5. Extracellular vesicles secreted by MHC class ii expressing melanoma cells and their role in immune escape and cancer progression

Francesca Costantini^1^, Caterina Di Sano^2^, Samuele Raccosta^3^, Mauro Manno^3^, Giovanna Barbieri^1^

^1^IRIB-CNR, Via Ugo La Malfa 153, Palermo, Italy.

^2^IFT-CNR, Via Ugo La Malfa 153, Palermo, Italy.

^3^IBF-CNR, Via Ugo La Malfa 153, Palermo, Italy.

Melanoma, one of the most widespread cancers in the Western population, is the deadliest form of skin cancer whose incidence rate will increase this year, making melanoma the second most diagnosed cancer. Melanoma cells secrete in their microenvironment extracellular vesicles that, for their nanoscale size, can circulate in advancing tumor front and in distant tissues, interacting with immune cells and different cell types as mediators of metastasis. Indeed, the metastatic progression of melanoma is associated with the expression of Major Histocompatibility Complex (MHC) class II molecules that are constitutively expressed in almost 50% of melanoma. Although MHC class II molecules expressed in melanoma cells may present tumor antigens to CD4+ T cells and trigger their effector functions, the constitutive MHC class II expression in melanoma is related to a bad prognosis. Therefore, the aim of our work was to understand the consequences of MHC class II-mediated signaling on extracellular vesicles secreted by melanoma cells. Indeed, we showed in extracellular vesicles secreted by class II constitutive expressing melanoma cell lines that the MHC class II-mediated signaling increases the expression of HLA-DR, adhesion receptors, PDL1, and STAT3. Furthermore, through co-culture experiments of PBMCs and extracellular vesicles, we showed that MHC class II-mediated signaling enhances the cytotoxic effects of extracellular vesicles on PBMCs. Indeed, our results suggest that MHC class II-mediated signaling plays a new role in promoting melanoma progression, enhancing, through the extracellular vesicles secreted, the metastatic dissemination of melanoma cells and inhibiting the immune response.

## 6. Implementation of membrane-sensing-peptide technology in a multiparametric flow cytometry assay: a diagnostic platform for monitoring allograft rejection

Stefano Panella^1^, Ilaria Barison^1,2,3^, Jacopo Burrello^1,4^, Roberto Frigerio^5^, Elena Provasi^1^, Chiara Castellani^3^, Marny Fedrigo^3^, Alessandro Gori^5^, Annalisa Angelini^3^, Marina Cretich^5^, Lucio Barile^1,2^

^1^Istituto Cardiocentro Ticino, Ente Ospedaliero Cantonale Lugano, Switzerland.

^2^Faculty of Biomedical Science, Università della Svizzera Italiana Lugano, Switzerland.

^3^Cardiovascular Pathology, Department of Cardiac, Thoracic and Vascular Sciences and Public Health, University of Padua, Padua, Italy.

^4^Division of Internal Medicine and Hypertension Unit, Department of Medical Sciences, University of Torino, Turin, Italy.

^5^National Research Council of Italy, Istituto di Scienze e Tecnologie Chimiche (SCITEC-CNR), Milan, Italy.

Circulating extracellular vesicles (EVs) are a promising tool for identifying cardiac allograft rejection and supplementing endomyocardial biopsy monitoring. However, due to variations in isolation, characterization, and the absence of standardized methods, it is challenging to compare results across studies and apply research findings to clinical practice. This study aimed to examine the feasibility of incorporating membrane sensing peptide (MSP) into flow-cytometer workflow for unbiased capture and surface antigen profiling of EVs, and validate the diagnostic platform using clinical samples from heart transplant patients.

We conjugated MSP on Ni-NTA beads to perform EV capture in plasma samples. EVs were analyzed using multiparametric flow cytometry, evaluating the expression of different inflammatory markers known to be upregulated in subjects experiencing acute cellular rejection (ACR). We evaluated the ability to efficiently isolate EVs by measuring the level of expression and distribution trend of tetraspanins and verified that these results were consistently congruent between MSP technology and state-of-the-art bead-based assay. Furthermore, we found that specific antigens were significantly overexpressed onto EVs’ surface derived from rejecting patients compared to control subjects. For some of these markers, the assay was able to distinguish different grades of ACR.

Our data show that MSP functionalized beads can be used to capture and isolate EVs from serum and plasma with high specificity, minimizing co-precipitation of contaminants. This technology can be implemented in high-throughput multiparametric flow cytometry. Such assay represents a concrete and plausible diagnostic platform adaptable to diverse clinically relevant contexts, applicable also in heart transplant monitoring.

## 7. Study of sEV internalization by antigen-presenting cells

Valeria Barreca^1^, Lorenzo Galli^1^, Deborah Polignano^1^, Valentina Tirelli^2^, Massimo Sargiacomo^1^, Maria Luisa Fiani^1^

^1^National Center for the Global Health, Istituto Superiore di Sanità, Rome, Italy.

^2^Core Facilities Technical-Service, Istituto Superiore di Sanità, Rome, Italy.

Small extracellular vesicles (sEV) are gaining recognition as important mediators of intercellular communication, playing a crucial role in modulating immune response. Both normal and tumor cells release sEV, but it is unclear whether they are selectively or non-selectively taken up by recipient cells. Recent studies have shown that sEV of endosomal origin are coated with high mannose glycans on their surface.

The aim of this study was to investigate the mechanisms of sEV internalization by antigen-presenting cells, specifically immature dendritic cells (iDCs) expressing the mannose receptor (MR) on their surface. The glycosylation profile of both cells and sEV can be altered in hypoxic conditions, such as those found in the tumor microenvironment.

Tumor cells change their metabolism from oxidative phosphorylation to glycolysis, leading to changes in glycosylation patterns. We wanted to evaluate the MR-specific uptake of sEV secreted under normoxic or hypoxic conditions. Fluorescent sEV (Bodipy sEV) were produced by pulsing melanoma cells with BODIPY FL C16, a fluorescent palmitic acid analogue, isolated through differential ultracentrifugation and quantified by Flow Cytometry (FC). iDCs were used to determine the specific uptake of Bodipy sEV. The results confirmed that iDCs selectively internalize sEV via MR and prompted phenotypic changes in the DCs. These findings shed light on the immunomodulatory properties of sEV in the innate immune system. However, further research is required to fully understand the underlying mechanisms and utilize the therapeutic potential of these findings.

## 8. Blood derived extracellular vesicle as parkinson’s disease biomarker for the monitoring of neurodegeneration and rehabilitation

Gemma Lombardi^1,2^, Silvia Picciolini^3^, Alice Gualerzi^3^, Valentina Mangolini^3,4^, Francesca Rodà^3,5^, Luana Forleo^3^, Aurora Mangolini^3^, Silvia Ramat^6^, Stefano Giuseppe Doronzio^2,7^, Diego Longo^2,7^, Francesca Cecchi^2,7^, Marzia Bedoni^3^

^1^Dipartimento di Neuroscienze, Psicologia, Area del Farmaco e Salute del Bambino, Università di Firenze, Firenze, Italy.

^2^IRCCS Fondazione Don Carlo Gnocchi ONLUS, Firenze, Italy.

^3^IRCCS Fondazione Don Carlo Gnocchi ONLUS, Milan, Italy.

^4^Dipartimento di Medicina Molecolare e Traslazionale, Università degli Studi di Brescia, Brescia, Italy.

^5^Clinical and Experimental Medicine PhD Program, University of Modena and Reggio Emilia, Modena, Italy.

^6^Parkinson Unit, Azienda Ospedaliera Universitaria Careggi, Firenze, Italy.

^7^Dipartimento di Neuroscienze, Psicologia, Area del Farmaco e Salute del Bambino, Università di Firenze, Firenze, Italy.

Rehabilitation is crucial in the treatment of people with Parkinson’s disease (pwPD) as it can ameliorate motor and non-motor impairments, improving their clinical profile and quality of life. Considering the complex biological processes occurring in the PD brain, the identification of accessible and measurable biomarkers to monitor the events induced by intensive rehabilitation would help in testing rehabilitation effectiveness, improving the design of clinical trials, and personalizing rehabilitation strategies.

Extracellular vesicles (EVs) were proved to be vehicles of a-synuclein and other PD-related molecules throughout the body; a correlation was found between the clinical profiling of pwPD and the biochemical modifications of blood EVs. In the present study, 20 pwPD undergoing a rehabilitation program based on treadmill training (8 weeks) were recruited. Clinical, neuropsychological, and instrumental variables were collected before and after treatment, as well as blood samples. EVs were isolated from serum with a combinatorial procedure based on size exclusion chromatography and ultracentrifugation. The bulk biochemical characterization of blood-derived EVs was obtained by Raman Spectroscopy and spectral differences in the EV fingerprint before and after rehabilitation were evaluated. Raman data were then correlated with clinical and motor parameters to evaluate the ability of Raman spectra to be predictive biomarkers of rehabilitation efficacy.

The preliminary data of the present project support the use of Raman spectroscopy for the evaluation of disease-related modification in EV content and chemical properties to be used as a clinical decision support tool for diagnosis and monitoring of pwPD.

## 9. miRNA function and partitions are determined by consensus motifs and epitranscriptomic modifications

Sabrina Garbo^1^, Daniel D’Andrea^1^, Francesco Marocco^1^, Gioele Gaboardi^1^, Claudia Montaldo^2^, Carla Cicchini^3^, Cecilia Battistelli^1^, Marco Tripodi^1^

^1^Department of Biochemical Sciences "A.ROSSI FANELLI"; University of Rome La Sapienza; Rome, Italy.

^2^National Institute for Infectious Diseases L. Spallanzani, IRCCS, Rome, Italy.

^3^Istituto Pasteur Italia-Fondazione Cenci Bolognetti, Dipartimento di Medicina Molecolare, University of Rome La Sapienza, Rome, Italy.

While EVs-mediated transfer of microRNAs has been reported to contribute to intercellular communication, the knowledge about molecular mechanisms controlling the selective partition of miRNAs between intracellular and EVs compartments is still largely limited.

Remarkably, the interactions between specific RNA-binding proteins and short G-rich RNA sequences have been proved causal for the loading of multiple miRNAs in EVs. With respect to intracellular miRNAs, while in silico analysis demonstrated that they are enriched in specific sequence determinants (CL-motifs and the CELL-motif), the interacting proteins remained unknown. Here, the RBP Poly-C-binding protein 2 has been identified as a direct interactor of the (CL)-motif: RNA immunoprecipitation (RIP) after UV cross-linking, coupled to RNA pull-down, demonstrated that this protein directly binds to miRNAs embedding this sequence and mutagenesis of the motif proved the specificity of PCBP2 binding. Moreover, the presence of multiple players that determine miRNA compartmentalization has been highlighted; we find that the previously characterized miRNA EV loader SYNCRIP and PCBP2 may contemporarily bind to miRNAs endowed with both hEXO and cell retention motifs (RIP and EMSA assays). Mechanistically, SYNCRIP knock-down appears to limit PCBP2 recruitment, while PCBP2 knock-down enhances SYNCRIP binding and allows the EVs loading of specific intracellular microRNAs. Notably, the impact of epitranscriptomic modification has been investigated, highlighting a further level of complexity to be considered when relating the specific repertoire of miRNAs to cellular differentiation and plasticity. Our results indicate that cells express specific miRNAs that do not impact endogenous transcripts but instead provide regulatory information for cell-to-cell communication. Molecular mechanisms governing both miRNA function and partition will be discussed.

## 10. Comparative transcriptomic analyses on human intestinal organoids exposed to anisakis-derived extracellular vesicles

Ilaria Bellini^1^, Daniela Scribano^1^, Cecilia Ambrosi^2^, Claudia Chiovoloni^1^, Silvia Rondon^1^, Annamaria Pronio^3^, Anna Teresa Palamara^1,4^, Stefano D’Amelio^1^, Serena Cavallero^1,5^

^1^Department of Public Health and Infectious Diseases, Sapienza University of Rome, Rome, Italy.

^2^Department of Human Sciences and Promotion of the Quality of Life, San Raffaele Open University, IRCCS, Rome, Italy.

^3^Digestive Endoscopy Unit, Department of General Surgery and Surgical Specialties "Paride Stefanini", Sapienza University of Rome, Azienda Policlinico Umberto I, Rome, Italy.

^4^Department of Infectious Diseases, Istituto Superiore di Sanità, Rome, Italy.

^5^Department of Public Health and Infectious Diseases, Sapienza University of Rome, Laboratory affiliated to Istituto Pasteur-Fondazione Cenci Bolognetti, Rome, Italy.

Anisakiasis is an accidental zoonosis caused by the consumption of raw fish parasitized with infective third-stage larva of Anisakis spp (L3). In severe cases, symptoms could lead to ulcers, granuloma, and chronic inflammation of the gastrointestinal tract, features potentially involved in the onset of a carcinogenic microenvironment. Interestingly, case reports of gastric and intestinal tumors in co-occurrence with anisakiasis are increasing from endemic countries. Nevertheless, investigations on Anisakis pathogenicity in humans are still scarce. The aim of this study is to investigate Anisakis tumorigenic potential using: human intestinal organoids (HIO), a cutting-edge model capable of exhibiting the architecture and functionality of the organ of origin and a newly discovered Anisakis messenger of pathogenicity, the extracellular vesicles (EVs). A comparative transcriptomic analysis carried out on HIO treated with Anisakis-derived EVs revealed several transcripts showing potential links to cancer processes when the top 100 most abundant transcripts and the differentially expressed genes in treated HIO were examined. In particular, the downregulation of EPHB2 and LEFTY1 and the upregulation of NUPR1 genes, known to be associated with colorectal cancer, were suggested by bioinformatics and confirmed by qRT-PCR. Gene expression and protein estimation of inflammatory and cancer-related factors were also performed, suggesting an immunosuppressive action with a decreasing trend in Il33 and Il1β gene expression and no alteration in Il8. This study represents the first attempt to deepen the Anisakis tumorigenic potential in human infections using HIO and EVs.

## 11. Interaction and nanoplasmonics of lipoproteins and gold nanoparticles

Andrea Zendrini^1,2^, Jacopo Cardellini^2,3^, Roberto Frigerio^1,4^, Debora Berti^2,3^, Marina Cretich^4^, Paolo Bergese^1,2,5^

^1^Department of Molecular and Translational Medicine, Università degli Studi di Brescia, Viale Europa 11, Brescia, Italy.

^2^Center for Colloid and Surface Science (CSGI), Via della Lastruccia 3, 50019 Sesto Fiorentino, Firenze, Italy.

^3^Department of Chemistry “Ugo Schiff”, Università degli Studi di Firenze, Via della Lastruccia 3, 50019 Sesto Fiorentino, Firenze, Italy.

^4^Istituto di Scienze e Tecnologie Chimiche “Giulio Natta”─National Research Council of Italy (SCITEC-CNR), Milan, Italy.

^5^National Center for Gene Therapy and Drugs based on RNA Technology CN3, Milan, Italy.

Lipoproteins are micelle-like extracellular nanoparticles constituted by a hydrophobic core of non-polar lipids surrounded by an amphiphilic monolayer of phospholipids that also embed lipid-binding proteins. Lipoproteins act as lipid carriers in the bloodstream and their molecular components (e.g., cholesterol) are classically used as metabolic and disease biomarkers. As nanoparticles, lipoproteins are very recently being investigated as drug delivery vectors and adjuvant carriers for vaccines. The techniques to characterize/track lipoproteins at the molecular level are well-established, but the understanding of lipoprotein nanoscale (mesoscale) properties is poor and fragmented, although needed to achieve the full potential of lipoproteins in nanomedicine. Recently, citrate-capped gold nanoparticles (AuNPs) emerged as convenient, sensitive, and robust colorimetric probes to determine the concentration, stiffness, and presence of protein co-isolates in extracellular vesicle (EV) preparations. The working mechanism of such assays is based on the clustering of AuNPs onto the EV membrane, which is triggered by the substitution of the citrate molecules capping the AuNPs by the phospholipids from the outer leaflet of the membrane. In this contribution, we will provide the first experimental evidence that the interaction of AuNPs reported for EVs extends to lipoproteins. Such interaction leads to AuNP-lipoprotein hybrids and is sensitive to lipoprotein classes and AuNP/lipoprotein molar ratio. These findings build a new bridge connecting EVs and lipoproteins, candidate gold nanoplasmonics as an innovative analytical method to evaluate lipoprotein formulations at the mesoscale and to enable the realization of lipoprotein–inorganic nanoparticle hybrid systems with amazing properties ahead.

## 12. Syndecan-syntenin-alix complex mediates SRC function in macrophages’ small-EV release

Vanessa Biemmi^1,2^, Stefano Panella^1^, Jacopo Burrello^1,3^, Edoardo Lazzarini^1^, Yulia Goshovska^1,2^, Giuseppina Milano^4^, Lucio Barile^1,2^

^1^Cardiovascular Theranostics, Istituto Cardiocentro Ticino, Laboratories for Translational, Research, Ente Ospedaliero Cantonale Lugano, Lugano Switzerland.

^2^Faculty of Biomedical Science, Università della Svizzera Italiana Lugano, Lugano, Switzerland.

^3^Division of Internal Medicine and Hypertension Unit, Department of Medical Sciences, University of Torino, Turin, Italy.

^4^Department of Cœur-Vaisseaux, Centre Hospitalier Universitaire Vaudois, Lausanne, Switzerland.

Due to their ability to remain stable in all body fluids, Extracellular vesicles (EVs) can connect distant cells and regulate inflammatory processes by transferring small molecules. Macrophages (M0) are important cellular players in most inflammatory processes, including those occurring after myocardial infarction. M0 express endothelin receptor A (ETA) after maturation; the role of ETA in mediating EV release is not fully understood. In this study, we investigate the role of ETA as a regulator of SRC kinase in the pathway of inflammatory EV release.

Healthy volunteers' buffy-coat-derived M0 cells were differentiated into an inflammatory M1 phenotype via cytokine stimulation. Inhibition of ETA had a specific effect on reducing the amount of released small EV in both M0 and M1, while the secretion of large EV remained unaffected according to Particle-Metrix and flow cytometry analyses. The mechanism behind this effect involved a decrease in the activation of Src kinase (pSrc), which led to the impairment of the Syndecan-Syntenin-Alix complex formation. Additionally, the decrease in pSrc resulted in a reduction in the expression of Rab5, Rab7, and Rab27 proteins.

Our findings highlight the essential role of SRC kinase in regulating the biogenesis and release of inflammatory macrophages' EVs through the regulation of Syndecan-Syntenin-Alix sorting complex formation. This sorting mechanism could potentially impact the regulation of inflammatory processes mediated by small EVs from M1 macrophages in pathological conditions.

## 13. Extracellular vesicles as a potential source of biomarkers in endometriosis

Barbara Bortot^1^, Alessandro Mangogna^1^, Alice Gualerzi^2^, Silvia Picciolini^2^, Giovanni Di Lorenzo^1^, Federico Romano^1^, Gabriella Zito^1^, Ben Peacock^3^, Rebecca Lees^3^, Giuseppe Ricci^1,4^, Stefania Biffi^1,*^

^1^Institute for Maternal and Child Health, IRCCS Burlo Garofolo, Trieste, Italy.

^2^IRCCS Fondazione Don Carlo Gnocchi, Milan, Italy.

^3^NanoFCM Co., Ltd, Medicity Nottingham, Nottingham, UK.

^4^Department of Medical, Surgical and Health Science, University of Trieste, Trieste, Italy.

Endometriosis is a gynecological disease affecting 5%-10% of women of reproductive age, and it is characterized by endometrial-like tissue present outside of the uterus. Small EVs present in the microenvironment of the uterus and peritoneum may facilitate the expansion of ectopic endometrial cells beyond the confines of the uterus. This phenomenon is similar to the role of EVs in the formation of pre-metastatic niches and organotropism in malignant tumors. The primary aim of this research was to develop a quantitative EVs-based test for the monitoring of disease progression and treatment intervention in patients diagnosed with endometriosis. We isolated the small EVs from the peritoneal fluids of endometriosis patients and controls to assess a specific panel of indicators that exhibit a positive correlation with the disease. Prior research has demonstrated that there exists an interaction between platelets and endometriotic lesions, which contributes to the advancement of endometriosis and the establishment of a hypercoagulable state in females affected by endometriosis. The utilization of nano-flow cytometry for single-particle phenotyping analysis allowed us to define the degree of platelet activation, which is of interest since it will facilitate personalized patient evaluations. Moreover, we observed a modulation of some markers similar to what is found in the ascites of ovarian cancer patients. Raman spectra of EVs from the peritoneal fluids were also obtained. Overall, molecular signatures of disease are detectable in the peritoneal fluid, which is reflective of the microenvironment, and the characterization of endometriosis-specific small EVs opens up new avenues for the investigation of endometriosis.

## 14. *In vivo* models to study the anti-fibrotic and anti-oxidative mechanism of extracellular vesicles derived from umbilical cord mesenchymal stromal cells

Paola Bisaccia^1,2^, Fabio Magarotto^1,2^, Marcin Jurga^3^, Maurizio Muraca^2,4,5^, Michela Pozzobon^1,2^

^1^Stem Cells and Regenerative Lab, Institute of Pediatric Research Città della Speranza, Padua, Italy.

^2^Department of Women and Children Health, Università di Padua, Padua, Italy.

^3^EXO Biologics, Liège, Belgium.

^4^Institute of Pediatric Research Città della Speranza, Padua, Italy.

^5^Lifelab Program, Consorzio per la Ricerca Sanitaria (CORIS), Veneto Region, Padua, Italy.

Our previous *in vivo* experiments showed that mesenchymal stromal cell-derived extracellular vesicles (MSC-EVs) could counteract the development of Bronchopulmonary Dysplasia (BPD) in a rat model. Based on these results, to better understand the protective mechanisms against fibrosis and oxidative stress, two *in vitro* models were developed: the fibrosis model with primary rat macrophages and the oxidative stress model with human alveolar epithelial cells treated with MSC-EVs after damage.

EVs were produced following Good Manufacturing Practice (GMP-grade) using human Wharton’s-jelly derived MSCs. EVs were isolated by tangential flow filtration (TFF) and characterized according to MISEV2018. Macrophages from bone marrow were analyzed for αSMA and CD90 expression by flow cytometry after TGFβ1 treatment. Human alveolar epithelial cells were treated with rotenone, an inhibitor of complex I of the mitochondrial respiratory chain that led to Reactive Oxygen Species (ROS) production. Immunofluorescence for 8-oxo-dG, qRT PCR of the genes involved in the antioxidant response, and assessment of the production of mitochondrial superoxide and ROS in a time course of 7 days were performed.

MSC-EVs suppressed the induction of αSMA expression in macrophages. Rotenone led to DNA oxidation and ROS production. The addition of MSC-EVs reduced the pro-oxidative effects.

MSC-EVs counteract the development of fibrosis and the redox imbalance. These results can contribute to unraveling the mechanism of action of these nanoparticles in preventing the development of BPD, as previously demonstrated in a specific rat model.

## 15. Raman spectroscopic analysis of lipoproteins quality in obese and healthy subjects

Arianna Bonizzi^1^, Flavia Magri^1,2^, Serena Mazzucchelli^3^, Marta Truffi^1^, Andrea Rizzi^4^, Fabio Corsi^1,3^, Roberta Cazzola^3^, Carlo Morasso^1^

^1^Istituti Clinici Scientifici Maugeri IRCCS, Via Maugeri 4, Pavia, Italy.

^2^Department of Internal medicine and Medical Therapy, Università degli Studi di Pavia; C.so Strada Nuova, Pavia, Italy.

^3^Department of Biomedical and Clinical Sciences, Università degli Studi di Milan, Via G.B. Grassi 74, Milan, Italy.

^4^S.C. Chirurgia Generale Tradate, Ospedale Galmarini di Tradate, ASST dei Sette Laghi, Tradate, Italy.

Routine measurement of cholesterol (CL) levels transported in the lipoproteins (LPs) is one of the main clinical approaches used to assess the risk of cardiovascular events (CVD). Nevertheless, recent clinical studies demonstrate that the quantity of CL transported in blood does not fully capture the CVD risk.

Clinical interest thus focused on assessing the quality of LPs, as well as their lipid/protein composition and oxidative status. Furthermore, the level of antioxidants transported by LPs appears to be strictly related to their functionality.

In this scenario, Raman Spectroscopy (RS), a light scattering technique, could be a valid approach to rapidly obtain relevant information related to the quality of LPs.

The ability of RS to extract information on the chemical fingerprint of LPs was here tested in a clinical study aimed at profiling obese patients (Ob, *n* = 39) and a control group of healthy volunteers (HC, *n* = 26).

The study found that a careful evaluation of the peaks related to the molecular vibrations of CL, triglycerides, unsaturated fatty acids, carotenoids, and proteins provided an immediate measure of LPs’ quality. Clear differences emerged between the biochemical and oxidative state of LPs of Ob’s and HC.

Overall, these results unravel the effectiveness of RS as a viable approach to identifying the quality of LPs in different clinical populations and pave the road toward a more comprehensive understanding of the role of LPs in metabolic disorders.

## 16. Engineered red blood cell extracellular vesicles as a therapeutic strategy for the treatment of renal diseases

Alessia Brossa^1,2^, Alessandro Gori^3^, Marina Cretich^3^, Cristina Grange^4^, Ilaria Giusti^5^, Vincenza Dolo^5^, Malvina Koni^4^, Maria Felice Brizzi^4^, Benedetta Bussolati^1,2^

^1^Department of Molecular Biotechnology and Health Sciences, University of Torino, Turin, Italy.

^2^Molecular Biotechnology Center, University of Torino, Turin, Italy.

^3^Consiglio Nazionale delle Ricerche, Istituto di Chimica del Riconoscimento Molecolare (ICRM), Via Mario Bianco, 9, Milan, Italy.

^4^Department of Medical Sciences, University of Turin, Corso Dogliotti 14, Turin, Italy.

^5^Clinical Pathology Unit, Department of Life, Health and Environmental Sciences, University of L'Aquila, L'Aquila, Italy.

The use of engineered extracellular vesicles (EVs) for clinical purposes represents a novel strategy in regenerative medicine. However, cellular EV sources are limited in terms of availability and safety. Red blood cell-derived extracellular vesicles (RBC-EVs) could be used for large-scale production of engineered EVs with therapeutic potential and for autologous therapy. In literature, different approaches have been described to exploit RBC-EVs as a drug delivery system. We are currently evaluating different protocols of RBC-EVs engineering that would allow the loading of therapeutic RNAs and peptides. RBC-EVs have been isolated from healthy donors using ultracentrifugation and tangential flow filtration, in order to scale up EV production. RBC-EVs are characterized by nanoparticle tracking analysis, transmission electron microscopy (TEM), and super-resolution microscopy, in order to verify their number, dimension, purity, and marker expression. TEM analysis showed intact RBC-EVs with a diameter of around 50 nm. RBC-EVs express the tetraspanins CD9, CD63, CD81, and the RBC marker CD47, as evaluated by super-resolution microscopy. EV loading is performed using cholesterol-modified miRNAs and a peptide targeting KIM-1, specifically expressed in injured proximal tubular cells, to obtain specific cell targeting and increase the delivery efficiency. Engineered RBC-EVs will be tested on *in vitro* models of renal cell carcinoma using renal cancer stem cells, and of acute and chronic kidney damage using a temperature-sensitive human renal proximal tubule cell line. We aim to obtain an easy scalable protocol for RBC-EVs isolation and engineering that would allow the broadening of the therapeutic applications of extracellular vesicles in regenerative medicine.

## 17. Small-rna sequencing of human urinary extracellular vesicles reveals the renal pro-inflammatory role of a high-sodium diet

Fabrizio Buffolo^1^, Jacopo Burrello^1^, Barbara Pardini^2^, Giuseppe Matullo^3^, Giovanni Birolo^2^, Margherita Alba Carlotta Pomatto^3^, Paolo Mulatero^1^, Brooke Honzel^4^, Yvonne Niebuhr^4^, Anand Vaidya^4^, Silvia Monticone^1^

^1^Division of Internal Medicine and Hypertension Unit, Department of Medical Sciences, University of Torino, Turin, Italy.

^2^Molecular and Genetic Epidemiology, Italian Institute for Genomic Medicine (IIGM), Turin, Italy.

^3^Department of Medical Sciences, University of Torino, Turin, Italy.

^4^Center for Adrenal Disorders, Brigham and Women's Hospital, Harvard Medical School, Boston, MA, USA.

High sodium intake is the most important acquired risk factor for the development of arterial hypertension. Beyond hemodynamic mechanisms, high sodium diet (HSD) increases the risk of hypertension through pro-inflammatory pathways. However, these mechanisms have been mainly investigated in preclinical models.

The goal of our study is to assess the role of HSD in renal pathophysiology through the evaluation of small-RNA cargos of urinary extracellular vesicles (uEVs). Fourteen subjects without hypertension were prospectively enrolled. Each patient underwent 5-7 days of a low-sodium diet and HSD, with tight control of compliance with 24-h urinary sodium excretion. The uEVs were isolated from a 24 h urine collection at the end of each diet phase by ultra-centrifugation, characterized and compared through miRNA sequencing analysis.

HSD induced a significant reduction of plasma renin activity and aldosterone levels (22.1 *vs.* 7.9 ng/dl, *P* < 0.001). The miRNA sequencing identified 111 small RNAs, including 30 small RNAs differentially expressed. Pathways and network analysis showed enrichment of cellular stress and immune system pathways after HSD, while pathways related to peroxisome proliferator-activated receptor alpha (PPARα) regulation were inhibited in HSD. *In vitro*, the inhibition of miR-320b, which is downregulated in HSD, increases intercellular adhesion molecule 1 (ICAM-1) in proximal tubular cells. Mimicking of miR-10b-5p, which is upregulated in HSD, reduces PPARα, which has an anti-inflammatory role in the kidney.

In conclusion, the small-RNA characterization of human uEVs reveals that HSD has a pro-inflammatory role at the renal level, potentially contributing to the development of hypertension and kidney damage.

## 18. Agri-food byproducts: from waste to resource for isolation of nanovesicles

Raffaella Latella^1^, Lorena Urbanelli^1,4^, Elisabetta Chiaradia^2^, Anna Maria Salzano^3^, Alessia Tognoloni^2^, Roberto Maria Pellegrino^1^, Husam Alabed^1^, Giada Cerotti^1^, Brunella Tancini^1^, Andrea Scaloni^3^, Carla Emiliani^1,4^, Sandra Buratta^1,4^

^1^Dept. Chemistry, Biology and Biotechnology, University of Perugia, Perugia, Italy.

^2^Dept. Veterinary Medicine, University of Perugia, Perugia, Italy.

^3^ISPAAM, CNR, Naples, Naples, Italy.

^4^Extracellular Vesicles network (EV-net) of the University of Perugia, Perugia, Italy.

The growing global population and the progress in technologies lead to an increasing demand for food production and the associated processing industry with a consequent generation of huge amounts of food waste and byproducts with a significant impact on the environment, economy, and society. However, agri-food waste is rich in bioactive high-value compounds (i.e., phenolic compounds, vitamins, carotenoids, *etc.*), exhibiting a wide range of health-promoting effects that are extracted with different techniques for nutritional and pharmacological applications. Here, we evaluated a possible alternative approach for waste valorization consisting of their use for the isolation of NanoVesicles (NVs), natural carriers of bioactive compounds. In particular, NVs were isolated from the Olive Vegetation Water (OVW), a byproduct generated during olive oil production, and from Serum-Milk (SM) obtained during the production of Grana Padano cheese. Both of them are highly polluting, difficult to dispose of and produced in large quantities. NV-OVW were isolated through differential centrifugation followed by density-gradient centrifugation, whereas NV-SM were isolated by differential centrifugation and by a Nichel-Based Isolation method. NV-OVW and NV-SM have been submitted to biophysical characterization through Scanning Electron Microscopy, Nanoparticle Tracking Analysis, and Cryo-Transmission Electron Microscopy. Then, NV-OVW and NV-SM were biochemically characterized by profiling their protein and lipid cargo. Understanding the biomolecules packaged in waste-derived NVs is a crucial step in predicting their biological effects on mammalian cell lines, which represents the final goal of this study.

## 19. Cardiovascular risk, metabolic profile and inflammatory fingerprint of obese subjects who underwent bariatric surgery

Jacopo Burrello^1,2^, Mary Julieth Gonzalez^3^, Lorenzo Airale^2^, Alessio Burrello^4^, Thomas Köstler^5^, Urs Zingg^5^, Lucio Barile^1^, Elena Osto^3^

^1^Laboratory for Cardiovascular Theranostics, Cardiocentro Ticino Institute, Bellinzona, Switzerland.

^2^Division of Internal Medicine & Hypertension Unit, Department of Medical Sciences, University of Torino, Turin, Italy.

^3^Institute of Clinical Chemistry, University and University Hospital of Zurich, Zurich, Switzerland.

^4^Department of Electrical, Electronic and Information Engineering (DEI), University of Bologna, Bologna, Italy.

^5^Department of General Surgery, Limmattal Hospital, Schlieren, Switzerland.

Bariatric surgery has emerged as an effective treatment for obesity, resulting in substantial and durable weight loss and improvement in several obesity-related comorbidities. Extracellular vesicles (EVs) reflect endothelial dysfunction and inflammation, and may predict post-surgical outcomes in these patients. The aim of our study was to exploit an EV signature to assess cardiovascular (CV) risk and metabolic-inflammatory profile before and after bariatric surgery.

We enrolled 62 patients; for each subject, clinical/biochemical parameters, disease status, medications, and EV profiling were evaluated at baseline (T0) and 1-/3-years (T1/T2,) after surgery (sleeve-gastrectomy or Roux-en-Y-gastric-bypass). EVs were isolated from serum by beads-based immuno-capture and analyzed for the expression of membrane-associated antigens.

After bariatric surgery, patients gradually lost weight (weight loss 31 Kg; 29.9% BMI reduction). Accordingly, the overall CV risk and metabolic-inflammatory profile of patients improved: blood pressure, HbA1c, triglycerides, LDL, uric acid, white blood cells, and C-reactive protein decreased, while HDL and renal function (eGFR) increased at follow-up; the prevalence of hypertension, dyslipidemia, and diabetes decreased together with the number of assumed drugs. Consistently, expression levels of EV-specific markers (CD9-CD63-CD81) and 11 out of the 37 evaluated antigens (from endothelium, platelets, and inflammatory cells) decreased at T1/T2, reflecting changes in main CV risk indicators. Interestingly, lower baseline levels of CD4-CD31-CD40-CD42a-CD62P were associated with a complete post-surgical outcome, defined as no residual disease without medications, with a BMI at T2 lower than 30 Kg/sqm.

EV-derived biomarkers reflected the improvement of CV profile in patients who underwent bariatric surgery and may become a new tool to predict post-surgical outcomes.

## 20. Extracellular vesicles from plant biotechnology platforms: potential applications for human health

Elisa Cappetta^1^, Mariapia Vietri^1^, Marisa Conte^1^, Valentina Santoro1, Nunziatina De Tommasi^1^, Fabrizio Dal Piaz^2,3^, Mariaevelina Alfieri^4^, Antonietta Leone^1^, Alfredo Ambrosone^1^

^1^Department of Pharmacy, University of Salerno, Via Giovanni Paolo II 134D, Fisciano, Italy.

^2^Department of Medicine, Surgery and Dentistry “Scuola Medica Salernitana”, University of Salerno, Baronissi, Italy.

^3^Operative Unit of Clinical Pharmacology, University Hospital “San Giovanni di Dio e Ruggi d’Aragona”, Salerno, Italy.

^4^Clinical Pathology, Santobono-Pausilipon Children’s Hospital, Naples, Italy.

Non-mammalian sources of extracellular vesicles (EVs) may provide alternative tools for therapeutic applications. For instance, plant-derived nano and microvesicles (PDEVs) have shown important antioxidant, antitumor and antimicrobial properties. However, the safe use of PDEVs for biomedical purposes is still limited mainly due to the lack of standard protocols for plant EVs isolation and the natural/seasonal variability of their biomolecular cargo. Importantly, current purification protocols based on destructive procedures lead to the production of artificially created EVs and make it difficult to distinguish between true EVs and other types of small vesicles.

To overcome these issues, we set up standardized procedures to obtain bioactive EVs from various plant *in vitro* cultures, including *hairy roots* (HRs) and cell suspensions (CSs) of different medicinal plants and crops.

By Dynamic Light Scattering, Nanoparticle Tracking Analysis and Scanning Electron Microscopy analyses, we showed that EVs purified from the conditioned medium of HRs and CSs have a round-shaped morphology and range in size prevalently between 100-200 nm. Proteomic and metabolomic approaches were carried out to profile the biomolecular cargo of such plant-derived EVs and identify species-specific biomarkers. Finally, we evaluated the antitumoral activity of HR-derived EVs, showing their selective and strong pro-apoptotic activity in pancreatic and breast cancer cells. Additional tests to study the neuroprotective and antimicrobial bioactivity of these EVs are currently on the way.

In conclusion, our approach represents a significant step towards the setup of new plant biotechnology strategies to produce nonconventional EVs with therapeutic interest.

## 21. Small extracellular vesicles in triple-negative breast cancer models

Loredana Casalis^1^, Beatrice Senigagliesi^2^, Pietro Parisse^3^

^1^Elettra Sincrotrone Trieste, Trieste, Italy.

^2^Bordeaux Neurocampus, Bordeaux, France.

^3^CNR-IOM, Trieste, Italy.

Small extracellular vesicles (sEVs, < 200 nm in diameter) are increasingly recognized as potent messengers in cell-cell and cell-extracellular matrix communication and have been shown to be involved in several cancer-related processes such as development, progression, niche preparation, and metastasis. However, their specific role in cancer spreading is still unclear. To shed light on that, we isolated (through ultracentrifugation) and thoroughly characterized sEVs derived from Triple Negative Breast Cancer (TNBC) cell lines, a clinically relevant model of metastatic cancer, focusing on the sEVs-induced modulation of the mechanical properties of non-metastatic target cells, measured by Atomic Force Microscopy (AFM). We found that TNBC-derived sEVs were able to induce a decrease in cell stiffness, rearrangements in cytoskeleton, focal adhesions, cellular morphology, increase in Yap downstream gene expression, and chromatin decondensation. Moreover, we explored the influence of ECM stiffness on sEVs release by plating TNBC cells on collagen-coated polydimethylsiloxane (PDMS) substrates at (two) different stiffnesses *vs.* standard glass substrate. After careful control of the cell growth conditions (vitality, morphology by immunofluorescence microscopy and AFM), we performed a multiparametric analysis of the TNBC-derived sEV based on complementary techniques including AFM and Asymmetric Flow Field Flow Fractionation with a Multi-Angle Light Scattering detector. Interestingly, we observed that soft substrates promote the release of a higher number of sEVs with larger dimensions with respect to harder ones. More data are needed to better understand ECM-sEVs release induction.

## 22. The impairment of the protein quality control system affects extracellular vesicle mirna secretion in cellular models of TDP-43 proteinopathies

Elena Casarotto^1^, Daisy Sproviero^2^, Letizia Messa^3^, Maria Garofalo^4^, Stephana Carelli^3^, Marta Cozzi^1^, Marta Chierichetti^1^, Riccardo Cristofani^1^, Veronica Ferrari^1^, Mariarita Galbiati^1^, Margherita Piccolella^1^, Paola Rusmini^1^, Barbara Tedesco^1^, Paola Pramaggiore^1^, Laura Cornaggia^1^, Guglielmo Patelli^1^, Cristina Cereda^5^, Stella Gagliardi^4^, Angelo Poletti^1^, Valeria Crippa^1^

^1^Dipartimento di Scienze Farmacologiche e Biomolecolari (DiSFeB), Department of Excellence 2018-2022, Università degli Studi di Milan, via Balzaretti 9, Milan, Italy.

^2^Fondazione IFOM Istituto Fondazione di Oncologia Molecolare, via Adamello 16, Milan, Italy.

^3^Centro di Ricerca Pediatrica "Romeo ed Enrica Invernizzi", Dipartimento di Scienze Biomediche e Cliniche "L. Sacco", Università degli Studi di Milan, via G.B. Grassi 74, Milan, Italy.

^4^Genomic and post-Genomic Center, IRCCS - Mondino Foundation, via Mondino 2, Pavia, Italy.

^5^UOC Screening Neonatale e Malattie Metaboliche, Dipartimento della Donna, della Mamma, del Neonato, ASST Fatebenefratelli Sacco - Ospedale dei Bambini "V. Buzzi", via Castelvetro 24, Milan, Italy.

TDP-43 proteinopathies are neurodegenerative diseases characterized by the abnormal accumulation of misfolded/aberrantly posttranslated species of the TAR DNA-binding protein-43 (TDP-43) in the cytosol of the affected cells. This condition is frequently associated with an impaired function of the Protein Quality Control (PQC) system (i.e., ubiquitin-proteasome system (UPS) and autophagy together with chaperone and co-chaperone proteins), which, together with the extracellular secretion, is responsible for TDP-43s homeostasis. Recently, we demonstrated that when the PQC is impaired, extracellular vesicles (EVs) [both large (LEVs) and small (SEVs)] are enriched with abnormal TDP-43 species and become harmful to recipient cells. Since EVs transport not only proteins but also miRNAs and miRNAs are deregulated in TDP-43 proteinopathies, we investigated whether the PQC impairment could also alter the miRNA content of EVs.

We isolated LEVs and SEVs from the culture medium of immortalized motoneuronal NSC34 cells treated or not with UPS or autophagy inhibitors (MG132 or NH4Cl, respectively) and analyzed their miRNA content. miRNA libraries were generated using Small RNA-Seq Library Prep Kit (Lexogen) and sequenced on a NextSeq 500/550 (Illumina). Interaction prediction was carried out on TarBase v.8 database. The results showed that miRNAs were differentially expressed (DE) both in MG132-SEVs and NH4Cl-SEVs. Interestingly, among DE miRNAs in treated-SEVs, 43 were in common and targeted mainly the prion disease pathway. These data suggest that in pathological conditions, EVs could contribute to the propagation of the disease by transporting both toxic TDP-43 species and potentially harmful miRNA.

## 23. Extracellular vesicles derived from human liver stem cells counteract chronic kidney disease development and cardiac dysfunction in remnant kidney murine model

Elena Ceccotti^1^, Massimo Cedrino^2^, Giulia Chiabotto^1^, Cristina Grange^1^, Alessandra Ghigo^3,4^, Alessandro Gambella^1^, Maria Felice Brizzi^1^, Giovanni Camussi^1,3^, Stefania Bruno^1,3^

^1^Department of Medical Sciences, University of Torino, Turin, Italy.

^2^Unicyte AG, Obendorf, Switzerland.

^3^Molecular Biotechnology Center, University of Torino, Turin, Italy.

^4^Department of Molecular Biotechnology and Health Sciences, University of Torino, Turin, Italy.

Chronic renal disease (CKD) is a serious clinical hurdle without adequate therapeutic strategies to prevent its progression.

The murine model of 5/6th partial nephrectomy with pole ligation (PNx) is the most suitable approach to mimic the human progressive renal failure and related uremic cardiomyopathy

In this study, we evaluated whether extracellular vesicles released from human liver stem cells (HLSC-EVs) may revert functional and histopathological alterations in PNx mice.

EVs were purified by ultracentrifugation and characterized in accordance with ISEV guidelines. PNx was performed in 10-week-old SCID mice by ligation of both poles of the left kidney, followed by removal of the right kidney one week later. EV treatment was performed weekly for 4 weeks, starting 1 month after the nephrectomy.

Renal function and cardiac function were evaluated using specific biochemical assays and echocardiography, respectively. Histological analyses were performed to quantify renal and cardiac interstitial fibrosis and glomerular sclerosis. The expression levels of genes involved in the development of fibrosis and inflammation were evaluated by real-time PCR.

PNx mice treated with EVs had an amelioration of renal function and showed a significant reduction of interstitial fibrosis, glomerular sclerosis, and capillary rarefaction. This trend of improvement was also confirmed by the statistically significant modulation of fibrotic and inflammatory markers. Moreover, EV treatment ameliorated cardiac function and significantly reduced interstitial fibrosis, which is a key hallmark of diastolic dysfunction.

In PNx mice, EV administration interferes with the development of CKD and ameliorates cardiomyopathy.

## 24. Targeting of the tumor extracellular matrix by click chemistry-based surface engineering of extracellular vesicles

Maria Chiara Ciferri^1^, Nicole Rosenwassen^1^, Enrico Millo^1^, Daniele Reverberi^2^, Silvia Bruno^1^, Cansu Gorgun^1^, Rodolfo Quarto^1,2^, Roberta Tasso^1,2^

^1^Department of Experimental Medicine (DIMES), University of Genova, Genova, Italy.

^2^IRCCS Ospedale Policlinico San Martino, Genova, Italy.

EVs have been recently considered a new class of effective delivery vehicles thanks to their role as mediators in intercellular communication. EV surface can be modified to incorporate ligands, making natural nanoparticles valuable tools for targeted therapy of various diseases. In this study, we propose a two-step EV-engineering protocol to obtain a tissue-specific drug delivery system based on: (1) loading with a medicinal cargo and (2) surface functionalization with a fluorescent peptide against an extracellular matrix oncofetal variant of the fibronectin (fibronectin extra-domain B, EDB) associated with tumor progression. After comparing different isolation methods to find the one with the optimal compromise between purity and yield, plasma EVs were isolated from healthy donors by sucrose cushion ultracentrifugation (sUC). Drug (Paclitaxel - PTX) loading was achieved by sonication, while membrane functionalization, with the anti-EDB azo-FITC- peptide, was obtained through a copper-free click chemistry reaction. The achievement of the reaction was evaluated by flow cytometry and the targeting efficiency was analyzed by ELISA and by 2D/3D tumor cell culture models. The cytotoxic effect of PTX-loaded EVs on cancer cells was assessed by MTT assay. Both copper-free click chemistry and sonication turned out to be valid engineering techniques: about 40% of EVs were functionalized and a reduction by 50% of the cell viability was observed after PTX-loaded-EV treatment. In addition, *in vitro* experiments showed that clicked-EVs successfully target EDB-fibronectin. Taken together, our data suggest that the direct chemical labeling of EVs can positively improve their use in molecular imaging and as drug carriers.

## 25. Red blood cells-derived extracellular vesicles as targetable drug delivery vehicles

Maria Chiara Ciferri^1^, Kai Härkönen^2,3^, Juha Prittinen^2,3^, Ulla Impola^2^, Petra Ilvonen^2,4^, Henri Tuovinen^5^, Tapani Viitala^5^, Enrico Millo^1^, Roberta Tasso^1,6^, Saara Laitinen^2^

^1^Department of Experimental Medicine (DIMES), University of Genova, Genova, Italy.

^2^Finnish Red Cross, Blood Service, Helsinki, Finland.

^3^A.I. Virtanen Institute, University of Eastern Finland, Joensuu, Finland.

^4^Doctoral program of Drug Research, University of Helsinki, Helsinki, Finland.

^5^Faculty of Science and Engineering, University of Helsinki and Åbo Akademi University, Helsinki, Finland.

^6^IRCCS Ospedale Policlinico San Martino, Genova, Italy.

Because of their ability to transport functional cargoes and the possibility to modify their surface to incorporate ligands, EVs have been recently considered valuable delivery vehicles, especially for targeted therapy. Particularly, red blood cell-derived nanoparticles, which have been found to be beneficial in terms of yield, bioavailability, and drug loading, have shown promising potential as efficient delivery systems for the targeted delivery of drugs. In this study, we propose an exogenous method for the functionalization of nanoerythrosome surface (NanoEs) with a fluorescent peptide able to target the tumor extracellular matrix. Our strategy is based on the “classic” click chemistry reaction of an azide with an alkyne (Huisgen cycloaddition), without copper. In this method, a DBCO-NHS ester tied to the NanoE-membrane links to an azo-fluorescent peptide which can recognize an oncofetal variant of the fibronectin (EDB-fibronectin), highly expressed by the tumor extracellular matrix. Flow cytometry was selected to (1) detect the fluorescent signal associated with an accomplished NanoE functionalization and (2) evaluate whether NanoE staining with CFSE can affect the efficiency of the click chemistry. Moreover, *in vitro* experiments, analyzed by Ligand Tracer technology and Imaging flow cytometry, were conducted on a cancer cell line to examine the interaction and subsequent internalization of anti-EDB-NanoEs. Click chemistry turned out to be a successful targeting system (about 50% of engineered NanoEs) and functionalized NanoEs were efficiently internalized by responder cells. Our EV membrane represents a promising tool that can be potentially applied both for molecular imaging and the targeted therapy of various pathological conditions.

## 26. Developing liver spheroids as a model to investigate the role of colorectal cancer-derived small extracellular vesicles in metastatic niche establishment

Elisa Costanzo^1^, Ornella Urzi’^1^, Marzia Pucci^1^, Marta Moschetti^1^, Alice Conigliaro^1^, Marco Loria^1^, Maria Cristina Guerrera^2^, Riccardo Alessandro^1^, Simona Fontana^1^

^1^Department of Biomedicine, Neuroscience, and Advanced Diagnostics, University of Palermo, Palermo, Italy.

^2^Department of Veterinary Sciences, University of Messina, Messina, Italy.

Colorectal cancer (CRC)-derived liver metastases represent the leading cause of CRC-related deaths. Metastasis establishment is preceded by the formation of metastatic niche (MN), a supportive microenvironment for metastatic cell colonization, which is highly impacted by tumor-derived small extracellular vesicles (sEVs). Our previous study demonstrated that CRC-derived sEVs (CRC-sEVs) induce in healthy hepatocytes (Heps) the epithelial-mesenchymal transition, an early event leading to liver fibrosis. Our goal is now to better investigate the pro-fibrotic effects of CRC-sEVs in Heps, thus outlining the events underlying MN establishment. Since liver fibrosis is overly complex to be represented in a bidimensional system, we have developed a tridimensional model of human healthy hepatocytes spheroids (h-HeSphs) characterized by dimension and other physical parameters.

sEVs isolated from CRC cell line SW480 were used to treat h-HeSphs, obtained by seeding human healthy hepatocytes (THLE-2) in ultra-low attachment 96 well plates. After evaluating the CRC-sEVs uptake by the h-HeSphs, we assessed the CRC-sEVs’ ability to alter the expression of hepatocytes’ structural and functional markers, such as apolipoprotein E, albumin, and cytokeratins 8/18. Moreover, by co-culturing h-HeSphs with SW620 cells, the metastatic counterpart of SW480 cells expressing green fluorescent protein (SW620-GFP), we revealed that CRC-sEVs treatment enhanced tumor cell invasiveness, thus suggesting that CRC-sEV-conditioned Heps can actively support metastatic colonization of liver.

Taken together, these results, beyond showing HeSPHs model suitability to study tumor-derived sEVs’ role in modulating target cell behavior, reveal that CRC-sEVs can affect Heps properties, inducing them to acquire an active role in shaping the liver metastatic niche.

## 27. Urinary extracellular vesicles fatty acid profiling captures lipid metabolism changes in childhood steroid-sensitive nephrotic syndrome

Giulia Cricri’^1^, Stefano Turolo^2^, Linda Bellucci^1^, Chiara Tamburello^2^, Irene Paraboschi^3^, Gianantonio Manzoni^3^, William Morello^2^, Giovanni Montini^1,2,4^, Federica Collino^1,2,4^

^1^Laboratory of Translational Research in Paediatric Nephro-urology, Foundation IRCCS Ca' Granda Ospedale Maggiore Policlinico, Milan, Italy.

^2^Pediatric Nephrology, Dialysis and Transplant Unit, Foundation IRCCS Ca' Granda Ospedale Maggiore Policlinico, Milan, Italy.

^3^Pediatric Urology Unit, Foundation IRCCS Ca' Granda Ospedale Maggiore Policlinico, Milan, Italy.

^4^Department of Clinical Sciences and Community Health, University of Milan, Milan, Italy.

Kidney inflammation is a critical contributor to progressive renal injury in pediatric Steroid-Sensitive Nephrotic Syndrome (SSNS). Altered blood Fatty Acid (FA) levels classically observed in SSNS can lead to modifications in cellular lipid metabolism, triggering inflammation. In this context, urine-derived Extracellular Vesicles (uEVs) can unveil the FA changes related to kidney dysfunction in SSNS children at different clinical phases.

EVs were isolated from urine (uEVs) of SSNS children at the onset of the disease (Onset), relapse (Rel), and remission (Rem) by ultracentrifugation. EVs number, measured by NTA, was higher in all the different clinical phases (*P* < 0.01) compared to controls (CTRL), while the size distribution was different only at onset compared to both CTRL and SSNS Rem (*P* < 0.05). Significant variations in the levels of saturated FA, mono- and polyunsaturated FA, analyzed by gas chromatography, were found in uEVs from Onset and Rel SSNS compared to CTRL and Rem patients. Onset SSNS patients presented reduced levels of stearic acid (18:0, *P* < 0.05) and an abundance of α-linolenic acid (18:3n3) (*P* < 0.01) compared to CTRL and of lignoceric acid (24:0), and DHA (22:6n3) compared to both CTRL (*P* < 0.01) and Rem SSNS (*P* < 0.05). The 24:0 and 22:6n3 levels were significantly changed in Rel patients as in the Onset group (*P* < 0.05 versus CTRL). Interestingly, the FA profile during the remission phase was still different from that of CTRL patients (18:3n3, EPA 20:5n3 and 24:1; *P* < 0.05). Lastly, Rel SSNS showed a strong reduction in *omega-3/-6* ratio, associated with an inflammatory milieu.

These data corroborate the use of uEVs FA content for monitoring the occurrence and progression of childhood SSNS.

## 28. Small extracellular vesicle miRNA cargo profiling in human glioblastoma preclinical *in vivo* models as candidate biomarkers for liquid biopsy

Laura Vilardo^1^, Ingrid Cifola^1^, Giuliana Gatti^2^, Nicolò Panini^3^, Alessio Torcinari^4^, Fabrizio Bonaventura^4^, Tiziana Orsini^4^, Francesca De Santa^4^, Myriam Catalano^5^, Marcello Raspa^3^, Igea D’Agnano^1^

^1^CNR, Institute for Biomedical Technologies, Segrate, Italy.

^2^Department of Medical Biotechnology and Translational Medicine, University of Milan, Milan, Italy.

^3^Flow Cytometry Application Resource Service Unit, Neurogenomics Research Centre, Human Technopole, Milan, Italy.

^4^CNR, Institute of Biochemistry and Cell Biology, Monterotondo, Italy.

^5^Department of Physiology and Pharmacology, Sapienza University, Rome, Italy.

Extracellular vesicles (EVs) are candidate next-generation biomarkers for liquid biopsy-based cancer diagnosis and monitoring disease recurrence in cancer patients. Next to well-established, cancer-associated components in the blood, such as circulating tumor cells and cell-free DNA, an increasing number of studies have highlighted EVs as valuable carriers of novel cancer biomarkers. However, the lack of standardized and clinically feasible protocols for EV purification and characterization still limits the applicability of EV-based tumor biomarker analysis.

Glioblastoma (GBM) is one of the most aggressive and lethal forms of primary brain tumors. EVs released by GBM cells can stimulate their own malignancy by suppressing the immune response or affecting the tumor microenvironment.

We developed a human GBM xenograft mouse model to identify circulating small EVs (sEVs)-enriched miRNAs secreted in the blood specifically by tumor cells. We previously studied the miRNA and protein content of the sEVs released by the U87 GBM cells in the culture medium, showing that the sEV-miRNA and -protein contents are not functionally correlated. We traced the U87 sEVs by transfecting the cells with a construct coding for a red fluorescent (dTomato, dT) tetraspanin CD81 fusion protein. To enrich the dT-CD81 cell population, we sorted the fluorescent cells and expanded them *in vitro*. We then characterized the sEVs released by the U87MG-dT-CD81 cells by microscopy, WB, and FACS. We injected the U87MG-dT-CD81 cells in NSG mouse brains and purified plasma circulating sEVs. We sorted by FACS the fluorescent sEVs (about 1% of total sEVs) and profiled the miRNA content by RNASeq.

## 29. Astrocytes-derived small extracellular vesicles hinder glioma growth

Mariassunta De Luca^1^, Carmela Serpe^1^, Lucia Monaco^1^, Arianna Rinaldi^1^, Igea D’Agnano^2^, Cristina Limatola^3^, Myriam Catalano^1^

^1^Department of Physiology and Pharmacology, Sapienza University, Rome, Italy.

^2^Institute of Biomedical Technologies, CNR, Segrate, Italy.

^3^Department of Physiology and Pharmacology, Laboratory Affiliated to Istituto Pasteur Italia Fondazione Cenci Bolognetti, Sapienza University, Rome, Italy.

In the brain, small extracellular vesicles (sEVs) represent a way used by all cells to exchange biological information (lipids, proteins, and nucleic acids such as mRNA, miRNA, and ctDNA) in the body. This happens during brain functional processes but also in pathological conditions (such as inflammation, neurodiseases, or cancer).

We focused the attention on the bidirectional crosstalk between healthy and cancer cells in the most common and malignant primary brain tumor, glioblastoma (GBM). GBM has a high rate of migration and invasion that permits a rapid growth of the tumor mass. Among healthy brain parenchymal cells, astrocytes play pro-tumoral or antitumoral roles depending on the stage of GBM progression. In this study, we show that astrocytes-derived sEVs (ADEVs) exert a defensive mechanism against tumor cell growth and invasion. We found that the effect is mediated by the transfer to tumor cells of factors that hinder glioma growth, reducing both tumor volume and tumor cell proliferation *in vivo* and prolonging glioma-bearing mice survival. Among many factors, we identified miR124 that is enriched in ADEVs. Among downregulated target genes of miR124, we found the volume-regulated anion channel (VRAC). VRAC channels regulate GBM cell migration and invasion. ADEVs, by increasing miR124 in GBM cells, reduce migration and invasion of these cancer cells.

In conclusion, astrocytes acutely promote the rebuilding of brain functionality in the context of GBM by the release of ADEVs enriched in miR124.

## 30. The role of soluble proteins in the biological functions of matrix vesicles

Maryanne Trafani de Melo^1,2^, Lucas Fabricio Bahia Nogueira^1,3^, Pietro Ciancaglini^1^, José Luis Millán^4^, Massimo Bottini^3,4^, Ana Paula Ramos^1^, Saida Mebarek^2^

^1^Faculdade de Filosofia, Ciências e Letras de Ribeirão Preto – USP, Departamento de Química, Ribeirão Preto, Brasil.

^2^University Lyon, Université Claude Bernard Lyon 1, CNRS UMR 5246, ICBMS, Lyon, France.

^3^Department of Experimental Medicine, University of Rome Tor Vergata, Rome, Italy.

^4^Sanford Burnham Prebys, La Jolla, USA.

During physiologic and ectopic calcifications, cells differentiated into a mineralizing phenotype shed from the microvilli, a special class of extracellular vesicles, also named matrix vesicles (MVs), which bind to the collagenous matrix and contribute to the formation of biological apatite. The biological functions of MVs have been assumed to be driven by their biochemical machinery. The MVs’ proteins have been described as native, that is, they are prearranged on the leaflets or within the lipid bilayer of the parent cells’ microvilli before the MVs are released. However, previous proteomic studies carried out by our group have revealed that several proteins of MVs do not have a microvilli origin, suggesting that a protein corona is formed on the surface of MVs. In this study, we have investigated if the protein corona modulates the ability of MVs to bind to a collagenous matrix and initiate biomineralization. MVs were isolated from MC3T3 and MOVAS cells, and their peripheral proteins were shaved off from the vesicles’ surface using a high-ionic-strength buffer. After this treatment, the isolated protein corona-devoid matrix vesicles (“shaved matrix vesicles” or SMVs) showed a distinct protein profile, a lower ability to mineralize, and a lower ability to bind to a collagenous matrix than native MVs. Our data strongly suggest that the peripheral soluble proteins might have a key role in the biological functions of MVs during both physiologic and ectopic mineralization processes.

## 31. Rognostic potential of urinary extracellular vesicle-micrornas for prostate cancer patients

Giuseppe De Palma^1^, Eleonora Torchia^2^, Michela Notarangelo^2^, Vito F. Di Lorenzo^3^, Antonio Tufaro^1^, Alessandro Mastrorosa^3^, Vito G. D’Agostino^2^

^1^Institutional BioBank, Experimental Oncology and Biobank Management Unit, IRCCS Istituto Tumori “Giovanni Paolo II”, Bari, Italy.

^2^Laboratory of Biotechnology and Nanomedicine, Department of Cellular, Computational and Integrative Biology (CIBIO), University of Trento, Trento, Italy.

^3^Urology Unit, IRCCS Istituto Tumori “Giovanni Paolo II”, Bari, Italy.

Prostate cancer (PCa) has become the most common cancer in the last decade in the male population of Western countries. Patients are subjected to invasive prostate biopsy to confirm PCa. One of the problems is that about 70%-80% of the prostate-tissue biopsies are deemed unnecessary. Thus, finding minimally invasive sources of biomarkers is of utmost importance. Limitations of biopsy histology may be addressed by complementary liquid biopsy testing.

Extracellular vesicles (EVs) are membranous particles secreted by cells in our fluids that carry heterogeneous molecular cargo, including nucleic acids, partially reflecting the cells of origin. It has been shown that EVs are effectively released in urine, and operative task forces are in place to improve protocols for urinary EVs (uEVs) recovery and characterization. Recently, several explorative studies indicated that uEV-RNA is a potential source of prognostic biomarkers for PCa, including both coding and (short/long) non-coding RNAs. We enrolled urine samples from three cohorts of subjects diagnosed with benign hyperplasia and PCa grouped according to Gleason scores. We recovered uEVs using differential ultracentrifugation or nickel-based isolation and profiled them by NTA. We observed that a fluorescence-based scatter mode relatively improved correlations between patient stratification and particle distribution. We performed uEV-RNA isolation and transcriptomic analysis by preparing cDNA libraries and NovaSeq6000 runs with a preference for miRNAs. Among several deregulated uEV-miRNA targets in reciprocal sample-grade comparisons, we found that miR-375, already known in prostate cancer progression, followed a trend of secretion coherent with disease grading and could contribute to liquid biopsy testing of prognostic biomarkers.

## 32. Interferon-gamma downregulates cd44 on extracellular vesicles via stat1 in a549 lung cancer cells

Dian J. Salih^1,2,3^, Katrin S Reiners^3^, Zulema Antonia Percario^4^, Loizzi Domenico^5^, Sollitto Francesco^5^, Martin Schlee^3^, Elisabetta Affabris^4^, Gunther Hartmann^3,#^, Teresa Santantonio^6,#^

^1^Department of Anatomy, College of Medicine, University of Duhok, Duhok, Kurdistan region of Iraq.

^2^Department of Clinical and Experimental Medicine, University of Foggia, Foggia, Italy.

^3^Institute of Clinical Chemistry and Clinical Pharmacology, University Hospital Bonn, Bonn, Germany.

^4^Department of Science, University Rome Tre, Rome, Italy.

^5^Department of Clinical and Surgical Sciences, University of Foggia, Foggia, Italy.

^6^Infectious Diseases Unit, Department of Clinical and Surgical Sciences, University of Foggia, Foggia, Italy.

Lung cancer is a leading cause of cancer-related deaths worldwide, and the identification of novel therapeutic targets and mechanisms underlying cancer progression is crucial to improving patient outcomes. Extracellular vesicles (EVs) have emerged as important mediators of intercellular communication and cancer progression. CD44, a transmembrane glycoprotein, has been implicated in cancer progression, including lung cancer. However, the regulation of CD44 lung cancer cells and EVs remains unclear. In this study, we explore the role of IFN-γ in regulating CD44 expression in A549 lung cancer cells and EVs and its dependence on the STAT1 pathway. Lung cancer cells [A549, wild type (wt), and STAT1-knockout] were treated with varying time points and concentrations of IFN-γ. We isolated EVs from conditioned media 24 or 48 h after treatment using differential ultracentrifugation. CD44 expression was analyzed in cells and EVs by flow cytometry and Western blotting. We confirmed STAT1 activation and translocation of phospho-STAT1 to the nucleus using immunofluorescent microscopy and Western blotting. The quantity and purity of EVs were assessed by nanoparticle tracking analysis (NTA), flow cytometry, and Western blotting.

We found that IFN-γ treatment downregulated CD44 expression in A549-wt cells, but not in A549 STAT1-knockout cells. This finding was also observed in EVs, with decreased CD44 levels in EVs derived from A540-wt IFN-γ-treated cells compared to STAT1-knockout cells.

IFN-γ-induced downregulation of CD44 in A549 lung cancer cells is STAT1-dependent and leads to decreased CD44 levels in EVs. To gain a full understanding of the underlying mechanisms and potential therapeutic implications of this regulatory pathway, further research is necessary.

Keywords: Lung cancer, extracellular vesicles, IFN-γ, CD44, STAT1.

## 33. Fruit-derived extracellular vesicle effects on a gastrointestinal barrier model of intestinal inflammation

Di Giulio Simona^#^, Mariano Stefania^#^, Carata Elisabetta, Elisa Panzarini

Department of Biological Sciences and Technologies, University of Salento, Lecce, Italy.

^#^Authors contributed equally.

Plant-derived extracellular vesicles (P-EVs) have been proposed as a potential nanomedicine for intestinal disorders; however, their impact on intestinal barrier integrity in gut inflammation has not been explored yet. It is widely known that a diet rich in fruits and vegetables preserves the integrity of the intestinal epithelial barrier, avoiding inflammatory stimuli and contributing to maintaining a “healthy gut”. The ability to transport bioactive molecules and the low toxicity give the P-EVs a remarkable versatility in the field of nanomedicine: the physicochemical stability in gastric and intestinal fluids makes them the ideal candidate for nutraceutical use. In this context, this study aimed to evaluate fruit-derived EVs on an *in vitro* gastrointestinal (GI) barrier model of intestinal inflammation through cytokines gene expression evaluation with RT-qPCR. *In vitro* model of the inflamed GI barrier is based on a triple culture obtained by using Caco-2, HT29, and Raji B cells co-cultured on filter cell culture chamber inserts (Transwell with polycarbonate filters and 0.4-um filter dimension) and exposed to dextran-sulfate sodium salt (DSS) and TNF-α. Cells were pretreated with P-EVs isolated from pomegranate, yellow grapes, lemon, and kiwi juice for 12 h before inflammation induction. P-EVs exert a protective effect on epithelial tight junction functionality and an antioxidant and anti-inflammatory activity upon internalization in Caco-2 cells visualized by confocal microscopy. These results showed the fundamental capability of P-EVs to modulate inflammation and their potential beneficial effect on intestinal mucosa.

## 34. Dynamic expression of CD169 in blood cells and circulating microvesicles as a potential marker in the development and progression of covid-19 and post-acute sequelae

Marialaura Fanelli^1^, Vita Petrone^1^, Rossella Chirico^1^, Christian Maracchioni^1^, Martina Giudice^1^, Elisabetta Teti^2^, Luigi Coppola^2^, Chiara Sorace^3^, Marco Iannetta^2,3^, Marta Zordan^2,3^, Pietro Vitale^3^, Loredana Sarmati^2,3^, Alexandre Lucas^4^, Emanuela Balestrieri^1^, Sandro Grelli^1,5^, Claudia Maria Radu^6^, Antonella Minutolo^1^, Claudia Matteucci^1^

^1^Department of Experimental Medicine, University of Rome Tor Vergata, Rome, Italy.

^2^Infectious Diseases Clinic, Tor Vergata Hospital, Rome, Italy.

^3^Department of Systems Medicine, University of Rome Tor Vergata, Rome, Italy.

^4^We-Met platform, Institut des Maladies Métaboliques et Cardiovasculaires (I2MC), plateau We-Met, Inserm UMR1297 and Université Paul Sabatier, Toulouse, France.

^5^Virology Unit, Policlinic of Tor Vergata, Rome, Italy.

^6^Thrombotic and Hemorrhagic Diseases Unit Department of Medicine - DIMED University of Padua, Padua, Italy.

An elevated inflammatory response and immune dysregulation are the main consequences of SARS-CoV-2 infection and characterize COVID-19 disease. This dysregulated inflammatory state persists even after infection, generating the post-acute sequelae of SARS-CoV-2 infection (PASC). The identification of new biomarkers that can characterize COVID-19 and predict its long-term effects is needed. CD169+ macrophages play an important role in viral infections, and recently, it has been demonstrated that CD169 was strongly overexpressed in the blood of COVID-19 patients (COV). Considering the close implication of CD169 expression in COVID-19, in the present study, the analysis of CD169 was also extended to PASC individuals, encompassing examinations of CD169 both on blood cells and circulating microvesicles (MVs) in association with serum inflammatory markers. Flow cytometry was used for the evaluation of HLA-DR and CD169 protein expression on leukocyte populations and MVs from 60 COV, 33 PASC, and 43 healthy donors (HD). Serum inflammatory markers were assessed by Ella Automated Immunoassay System. The median fluorescence intensity ratio of CD169 between monocytes and lymphocytes (CD169 RMFI) was found to be significantly higher in COV than in HD and PASC. Among the leukocyte populations, monocytes showed a significantly higher percentage of HLA-DR+CD169+ in COV and PASC than in HD and correlated with CXCL10 and the number of platelets. HLA-DR+CD169+ MVs were significantly elevated in COV and PASC compared to HD and correlated with coagulation factors. These data underline CD169 expression at the cellular level and circulating MVs as potential markers of COVID-19 and post-acute sequelae.

## 35. Circulating EV from unstable angina patients as a novel diagnostic marker to stratify different patient populations

Saveria Femminò, Alessandro Sarcinella, Alberto Grosso, Stefania Bruno, Ovidio De Filippo, Fabrizio D’Ascenzo, Maria Felice Brizzi

Department of Medical Sciences, Corso Dogliotti, 14, University of Turin, Turin, Italy.

Acute coronary syndrome (ACS) represents a major cause of hospitalization, disability, and death worldwide. Considering non-ST-segment elevation ACS (NSTE-ACS), unstable angina (UA) accounts for nearly 10% of ACS, although there is a significant decrease after hs-troponin assays implementation. Nevertheless, UA is associated with higher rates of future myocardial infarction and coronary revascularizations compared with non-UA patients, prompting the need for risk stratification. We have previously shown that circulating extracellular vesicles (EVs) from both patients with NSTE-ACS and UA were significantly enriched in troponin, suggesting possible myocardial distress/damage in UA. The present study aims to determine if troponin-enriched EVs, their cell of origin, and their miR cargo could be useful in stratifying different patient populations. ENDPOINTS: primary endpoint is a statistically significant difference in the expression of troponin-enriched EVs (%), miR cargo, and markers of their cell of origin between patients primarily referred to invasive vs non-invasive tests. Safety endpoint: major adverse cardiac events (MACE), a composite of all-cause mortality, recurrent myocardial infarction, unplanned coronary revascularization after the index event, or congestive heart failure requiring hospitalization. EVs recovered from 50 UA and 54 NSTE-ACS patients were analyzed. We demonstrated that, unlike NSTE-ACS, EVs from UA patients are mainly derived from platelets and are enriched in mir130a-3p and miR126-5p. A correlation between clinical features and troponin, miRs and specific platelet markers has been investigated, providing the bases to potentially stratify different patient populations.

## 36. On-chip device for flow-driven release of extracellular vesicles

Alessia Foscarini^1^, Valeria Garzarelli^1,2^, Antonio Turco^1^, Annamaria Nigro^3^, Maria Serena Chiriacò^1^, Elisabetta Primiceri^1^, Alessandro Romano^3^, Francesco Ferrara^1^

^1^CNR NANOTEC - Institute of Nanotechnology, Via per Monteroni, Lecce, Italy.

^2^University of Salento, Dept. of Mathematics & Physics E. de Giorgi, Via Arnesano, Lecce, Italy.

^3^Institute of Experimental Neurology, San Raffaele Scientific Institute, Milan, Italy.

Extracellular Vesicles (EVs) are cell-derived vesicles released from cells into their surrounding environment, with multiple roles encompassing cancer progression, immunological stimulation, and neurodegeneration.

Advanced microfabrication technologies enable the realization of Lab-On-Chip (LoC) devices to study biological phenomena. In particular, LoCs based on fluid dynamic simulations and microfabrication techniques (micro-milling and 3D printing) allow precise microenvironment control with high reproducibility.

This work aims to provide an innovative microfluidic device to study the release of EVs from different cell lines in response to dynamic conditions and mechanical stimuli. Molded plastic substrates assembled on a glass slide create a microfluidic chamber where Oral Squamous Carcinoma (OECM-1), neuroblastoma (SH-SY5Y), and microglial (CHME-5) cell lines were seeded, reaching 50% of confluence, and exposed to a controlled culture medium flow for different time-points. A complete medium replacement in the dynamic condition was obtained, setting the flow medium rate and avoiding shear stress. The large and small EVs released from cells in dynamic and static conditions were isolated by differential ultracentrifugation, and the different EV populations were characterized using high-resolution flow cytometry and western blot assays. We found that the release kinetics of EVs is affected by static or dynamic cell culture conditions.

Our microfluidic device mimics the dynamic physiological scenario at the cells’ surface where extracellular fluid is the carrier of EVs that can be absorbed or moved away, providing an on-demand customized microfabricated tool to unravel the still unknown mechanisms of EV biogenesis.

## 37. Plasma extracellular vesicles promote lung cancer pre-metastatic niche formation through endothelial modulation

Francesca Pontis^1^, Ilaria Petraroia^1^, Patrizia Ghidotti^1^, Mattia Boeri^1^, Sabina Sangaletti^2^, Paola Suatoni^3^, Ugo Pastorino^3^, Fabio Maiullari^4^, Roberto Rizzi^4^, Claudia Bearzi^4^, Gabriella Sozzi^1^, Orazio Fortunato^1^

^1^Unit of Epigenomics and Biomarkers of Solid Tumors, Fondazione IRCCS Istituto Nazionale dei Tumori, Milan, Italy.

^2^Molecular Immunology Unit, Fondazione IRCCS Istituto Nazionale dei Tumori, Milan, Italy.

^3^Thoracic Surgery Unit, Fondazione IRCCS Istituto Nazionale dei Tumori, Milan, Italy.

^4^Istituto Nazionale Genetica Molecolare INGM "Romeo Ed Enrica Invernizzi", Milan, Italy.

Lung cancer is the deadliest cancer worldwide, primarily because of metastatic spread. Extracellular Vesicles (EVs) play a crucial role in creating pre-metastatic niches (pMN). We aim to elucidate the role of plasma EVs in pMN establishment and identify new prognostic biomarkers.

Plasma EVs were obtained by ultracentrifugation from 40 early-stage patients who survived at 5 years (ESA-EVs) and 40 patients who died within two years (ESD-EVs). Heavy-smoker cancer-free individuals were used as control (HS-EVs). EV’s characterization was performed following MISEV guidelines. Functional experiments were carried out *in vitro* (2D and 3D-bioprinted models) and *in vivo*. EV’s miRNA cargo was investigated using Nanostring and digital PCR. Alongside common EV-related markers, plasma EVs express platelet and endothelial markers. *In vitro*, endothelial cells were the most avid EV incorporators among stromal cells. Functionally, ESD-EVs increased “pro-inflammatory” endothelial features (VCAM1, CXCR4, and CXCL1) in 2D and 3D models. *In vivo*, plasma EVs accumulated mainly in the lungs and liver, with less presence observed in the bone marrow. In detail, EVs were mostly taken up by endothelial cells and macrophages in the lung and liver and by CD45+/F480- in the BM. Moreover, more immunosuppressive Siglec F+/G-MDSC and NK cells were detected in the lungs of ESD-EV-treated mice than ESA- and HS-EVs. Furthermore, VCAM1, CXCL1, and IL-6 were increased in the lungs upon ESD-EV administration. Three miRNAs (miR-1307, miR-199a, miR-29a) were enriched in ESD-EVs compared to controls, and transient transfection of miR-29a resulted in endothelial cell activation.

Our findings suggested that miR-29 inside plasma EVs could drive lung pMN by endothelial cell activation.

## 38. Integrated diagnostic workflow for blood and urinary extracellular vesicles by membrane sensing peptides and digital detection

Marina Cretich^1^, Roberto Frigerio^1,4^, Paola Gagni^1^, Giulia Lodigiani^1^, Stefano Panella^2^, Jacopo Burrello^2^, Adele Tanzi^3^, Cristina Grange^3^, Lucio Barile^2^, Benedetta Bussolati^3^, Alessandro Gori^1^

^1^Consiglio Nazionale delle Ricerche, Istituto di Scienze e Tecnologie Chimiche (SCITEC-CNR), Milan, Italy.

^2^Istituto Cardiocentro Ticino, EOC, Bellinzona, Switzerland.

^3^Università degli Studi di Turin, Turin, Italy.

^4^Department of Molecular and Translational Medicine, Università degli Studi di Brescia, Viale Europa 11, Brescia 25123, Italy.

Extracellular Vesicles (EVs) are gaining increasing importance as potential biomarkers in many pathological conditions. This area of research faces big challenges due to EVs’ small size, low refractive index, huge heterogeneity, and high sensitivity demand in detecting low abundant disease-specific sub-populations. Such need can be met by innovative affinity probes and digital detection, namely capable of reaching single-molecule sensitivity. Small EVs present fairly distinctive lipid membrane features that could be considered a "universal" marker, alternative or complementary to traditional characteristic surface-associated proteins. Our recent work has identified Membrane Sensing Peptides (MSP) as a novel class of molecular ligands for integrated small EV isolation and analysis. The membrane recognition and binding mechanisms are based on complementary electrostatic interactions between the peptide and the phospholipids on the outer membrane leaflet, which subsequently can lead to the insertion of hydrophobic residues into the membrane defects. MSP can be used for the general capturing of all small EVs and subsequent unbiased immune phenotyping of surface antigens. Here, we present the integration of MSP onto the bead-based digital platform for Single Molecule Immunoassays and their application for isolation-free EV analysis from complex bio-samples. Specifically, we will first show a comparison of different bio-conjugation strategies of MSP onto beads for efficient EV capturing from urine, serum, and plasma, demonstrating the feasibility of our workflow directly in clinical samples. Then, we will report on a study on patient stratification by profiling serum EV-specific surface antigens known to be relevant for cardiovascular risk assessment (CD62p, CD42a, CD31) on MSP-modified beads by digital immune phenotyping.

## 39. Pan-specific, affinity isolation of small extracellular vesicles from minimally pretreated biological fluids by membrane sensing peptides

Alessandro Gori^1^, Roberto Frigerio^1,4^, Paola Gagni^1^, Giulia Lodigiani^1^, Stefano Panella^2^, Elena Provasi^2^, Adele Tanzi^3^, Cristina Grange^3^, Lucio Barile^2^, Benedetta Bussolati^3^, Marina Cretich^1^

^1^Consiglio Nazionale delle Ricerche, Istituto di Scienze e Tecnologie Chimiche (SCITEC-CNR), Milan, Italy.

^2^Istituto Cardiocentro Ticino, EOC, Bellinzona, Switzerland.

^3^Università degli Studi di Torino, Turin, Italy.

^4^Department of Molecular and Translational Medicine, Università degli Studi di Brescia, Viale Europa 11, Brescia 25123, Italy.

Affinity-based systems for isolation of Extracellular Vesicles (EVs) from complex biosamples are commonly plagued by poor recovery and often require a pre-concentration step. Here, we report an easy, timesaving, and robust protocol based on bead-based affinity isolation with Membrane Sensing Peptides (MSP), and we apply it to minimally pretreated biofluids (serum, plasma, and urine). The affinity protocol is based on the use of agarose beads carrying cation chelates that would specifically bind to the 6His-tagged MSP. EV downstream analyses can be performed on the agarose beads by simply adding lysis or nucleic-acid extraction buffers. Alternatively, EVs can be gently eluted by competition with imidazole or by a quick treatment in a saline solution for traceless EV release and subsequent characterization and functional analysis.

EV isolation is demonstrated with minimal carry-over of common contaminants such as lipoproteins for the blood-based workflow and uromodulin for the urinary EVs. A comparison of yield and purity is reported with standard procedures using antibody-modified beads, ultracentrifugation (UC), ultrafiltration (UF), and size-exclusion chromatography (SEC).

This new isolation methodology is based on affinity to general membrane characteristics of EVs, thus being not biased by the relative abundance of surface markers; the protocol can be used in EV samples from any species, including animal, plant, and bacterial vesicles for which antibodies have not yet been developed; it is suitable to small volumes for a clinical routine or can be scaled up according to operator needs.

## 40. Sorting of single lipoproteins from plasma and serum samples

Roberto Frigerio^1,2^, Alessandro Gori^1^, Paolo Bergese^2,3,4^, Marina Cretich^1^

^1^Consiglio Nazionale delle Ricerche, Istituto di Scienze e Tecnologie Chimiche (SCITEC-CNR), Milan, Italy.

^2^Department of Molecular and Translational Medicine, Università degli Studi di Brescia, Viale Europa 11, Brescia, Italy.

^3^Center for Colloid and Surface Science (CSGI), Via della Lastruccia 3, 50019 Sesto Fiorentino, Firenze, Italy.

^4^National Center for Gene Therapy and Drugs based on RNA Technology – CN3, Milan, Italy.

Cells secrete a huge variety of bio-nanoparticles with different physicochemical characteristics and functions, including Extracellular Vesicles (EVs) and lipoproteins (LPs), which play a crucial role in cell-to-cell communication, regulating physiological and pathological processes through the transfer of several biomolecules such as RNA, which can be taken up by other cells.

As has happened in the last 10 years for EVs, LPs as nanoparticles are gaining ground as a natural key player in nanomedicine, starting from drug delivery and liquid biopsy. LPs analysis is well established in clinical biochemistry at the molecular composition level, where they are quantified by collective (macroscopic) parameters such as the molar concentration of their characteristic molecular components, e.g., cholesterol and Apolipoprotein A or B. Separation, characterization, and manipulation at the single particle level are quite unexplored for LPs. Indeed, it is still challenging to individually sort the different types of particles contained in plasma or serum.

In our contribution, we will report on a new ultra-sensitive digital assay to detect lipoproteins at the single particle level with SiMoA (Single Molecule Assay) technology. This platform was previously applied by our group to analyze EVs in serum. In this new assay, paramagnetic beads are conjugated with an antibody directed to a specific Apolipoprotein and captured LPs are detected in a sandwich assay using the same antibody. This assay scheme limits the detectability only to those LP particles that express at least two molecules of the same Apolipoprotein, suggesting the detection of single particles. We applied this assay to sort LP particles in human plasma and serum, opening up new possibilities for diagnostic applications.

## 41. Engineered small extracellular vesicles for biogenesis and immunomodulation studies

Lorenzo Galli^1^, Deborah Polignano^1^, Valeria Barreca^1^, Roberta Bona^1^, Valentina Tirelli^2^, Massimo Sargiacomo^1^, Maria Luisa Fiani^1^

^1^National Center for the Global Health, Istituto Superiore di Sanità, Rome, Italy.

^2^Core Facilities Technical- Scientific Service, Istituto Superiore di Sanità, Rome, Italy.

Small extracellular vesicles (sEVs) are nanovesicles secreted constitutively by all cells and play a crucial role in intercellular communication and immune response regulation. As naturally occurring vesicles, they have a low intrinsic immunogenic profile but can be manipulated with genetic engineering to incorporate exogenous proteins to induce and enhance innate and acquired immunity. In our study, we use a mutant human immunodeficiency virus (HIV-1) Nef protein (Nefmut) defective for all pathogenic Nef functions, which presents an N-terminal palmitoylation domain, thereby improving association with lipid rafts and incorporation into sEVs. Nefmut is fused at its C-terminal with red fluorescent protein (Nefmut -mScarlet) or green fluorescent protein (Nefmut -GFP). To follow the biogenesis of these engineered sEVs, we use a methodology developed in our laboratory to metabolically label the membrane bilayer of sEVs with fluorescent palmitic acid analogs, BODIPY FL C16 (C16) or BODIPY 558/568 (C12). By transfecting HEK293T cells with the Nefmut -mScarlet or Nefmut vectors and pulsing them with C16 or C12, we obtained a mixed population of sEVs that can be purified and characterized. The results show that the number of cell-secreted sEVs greatly differs for the Nefmut -containing sEVs in comparison with Bodipy sEVs, which we have shown is a distinct subpopulation of sEVs, namely exosomes. Nefmut sEVs were characterized for typical exosome markers and analyzed in iodixanol density gradients. Further analysis will show if these different sEV populations display different behaviors in terms of efficiency of transfer to recipient cells and, ultimately, stimulation of the immune system.

## 42. Antioxidant effect of nanovesicles derived from lemon juice on hepatocytes

Roberta Gasparro, Giulia Duca, Vincenza Tinnirello, Nima Rabienezhad Ganji, Simona Fontana, Riccardo Alessandro, Stefania Raimondo

Dept. of Biomedicine, Neurosciences and Advanced Diagnostics (Bi.N.D), Section of Biology and Genetics, Palermo, Italy.

Nowadays, the biological applications of products derived from plants are attracting the attention of the scientific community; among those, nanovesicles from lemon juice (LNVs) have shown anti-inflammatory and antioxidant properties *in vitro* and *in vivo*. The liver is a complex organ that is frequently exposed to stimuli. These stimuli can determine the development of oxidative stress, which in turn can cause the development of several liver diseases. This project aims to study the protective effects of LNVs on healthy human hepatocytes (THLE-2) exposed to menadione, a well-known inducer of oxidative stress. Our findings demonstrate that LNVs can reduce the production of ROS in a statistically significant manner. Furthermore, we observed that Nrf2 and HO1, essential actors of the antioxidant response, are upregulated in hepatocytes after the pretreatment with LNVs. Several studies reported that Nrf2 can be activated by the phosphorylation of AKT. This protein is also involved in the first steps of Epithelial-Mesenchymal Transition (EMT), the process that can cause liver fibrosis. Consequently, we will further investigate the effect of LNVs on EMT markers. In conclusion, our preliminary results support the hypothesis of the protective role of LNVs against oxidative stress, which is one of the most common conditions that lead to the development of liver diseases.

## 43. Potential involvement of urinary and hepatocyte-derived extracellular vesicles in glyoxylate detoxification

Leonardo Gatticchi^1^, Rita Romani^1,2^, Barbara Cellini^1^, Ilaria Bellezza^1,2^

^1^Department of Medicine and Surgery, Section of Physiology and Biochemistry, University of Perugia, Perugia, Italy.

^2^Extracellular Vesicles network (EV-net), University of Perugia, Perugia, Italy.

Primary hyperoxalurias (PH) are rare genetic metabolic diseases of glyoxylate metabolism characterized by oxalate accumulation. The three types of PH (type 1, 2, and 3) are distinguished by the deficit in a specific oxalate detoxifying enzyme. PH2 is caused by the deficit in glyoxylate reductase/hydroxypyruvate reductase (GR/HPR), which catalyzes the conversion of glyoxylate into glycolate. GR/HPR deficit leads to glyoxylate accumulation, which is metabolized to oxalate by lactate dehydrogenase (LDH), resulting in kidney stone formation and nephrocalcinosis. GR/HPR activity is abundant in both liver and kidney.

Urine is a rich pool of EVs originating from cells of the urogenital tract. Urinary extracellular vesicles (uEVs) can reflect kidney damage, thus representing a source of diagnostic disease biomarkers for renal diseases, including primary hyperoxalurias. For example, uEVs contain molecular biomarkers which differ between PH1 patients with or without nephrocalcinosis or kidney stones.

We isolated EVs from HepG2 hepatic cells conditioned medium and from urine samples of healthy volunteers by sequential ultracentrifugation. Molecular characterization revealed the presence of EV markers, the absence of intracellular contaminant proteins, and the expression of GR/HPR and LDH. Both enzymes were active, and, most importantly, they can perform enzymatic activities in the intact EVs, indicating that EVs can uptake the substrates and release the reaction products. Our preliminary data suggest that hepatic and uEVs, by acting as independent metabolic units, can be directly involved in glyoxylate metabolism, thus possibly contributing to the pathogenesis of genetic forms of hyperoxaluria and representing possible diagnostic markers.

## 44. Effects of extracellular vesicles derived from human microglia cells on glioblastoma tumor microenvironment

Lorenzo Germelli^1^, Lorenzo Ceccarelli^1,2^, Laura Marchetti^1^, Milena Rizzo^3^, Aldo Moscardini^4^, Miriam Romano^5,6^, Chiara Giacomelli^1^, Paolo Bergese^5,6,7^, Claudia Martini^1^

^1^Department of Pharmacy, University of Pisa, Pisa, Italy.

^2^Department of Biotechnology, Chemistry and Pharmacy, University of Siena, Siena, Italy.

^3^Institute of Clinical Physiology (IFC), CNR, Pisa, Italy.

^4^SNS (Scuola Normale Superiore, NEST laboratories), Pisa, Italy.

^5^Department of Molecular and Translational Medicine, University of Brescia, Brescia, Italy.

^6^Center for Colloid and Surface Science (CSGI), Firenze, Italy.

^7^Institute for Research and Biomedical Innovation- IRIB,Consiglio Nazionale delle Ricerche CNR, Palermo, Italy.

Microglia are the major resident immune cells in the central nervous system (CNS) and represent the first line of defense against alterations in tissue homeostasis. Indeed, in glioblastoma, a large percentage of tumor-associated macrophages (TAMs) are microglia that, through a plethora of signaling pathways, modulate tumor progression. Small Extracellular Vesicles (EVs) are a component of the microglia secretome, and their cargo reflects the status of the parental cells with beneficial or detrimental effects in many physio-pathological conditions. Herein, the biophysical properties, miRNA cargo, and biological effects of EVs from human C20 microglia cells activated with the pro-inflammatory interleukin IL-1β were evaluated. In particular, inflammatory conditions increased the production of EVs. These EVs were characterized by a smaller mean diameter and higher miR-155-5p loading than EVs derived from unstimulated C20 cells. As expected, the EVs derived by inflamed cells prompted the pro-inflammatory phenotype, motility, and invasion capacity of receiving C20 cells, supporting the creation of an inflammatory tumor microenvironment. On the other hand, they significantly reduced the U87MG motility. Our data contribute to filling a gap in the knowledge of human microglia EVs, shedding light on their effects in the tumor microenvironment.

## 45. Immunoregulatory role of extracellular vesicles in advanced non-small cell lung cancer

Patrizia Ghidotti^1^, Diego Signorelli^2^, Claudia Proto^2^, Marta Brambilla^2^, Francesca Pontis^1^, Ilaria Petraroia^1^, Benedetta Bussolati^3^, Cristina Grange^3^, Gabriella Sozzi^1^, Elena Jachetti^4^, Orazio Fortunato^1^

^1^Epigenomic and Biomarkers of Solid Tumor Unit, Fondazione IRCCS Istituto Nazionale dei Tumori, Milan, Italy.

^2^Thoracic Oncology Unit, Fondazione IRCCS Istituto Nazionale dei Tumori, Milan, Italy.

^3^Department of Medical Sciences, University of Turin, Turin, Italy.

^4^Molecular Immunology Unit, Fondazione IRCCS Istituto Nazionale dei Tumori, Milan, Italy.

**Introduction:** Tumor PD-L1 is the only clinical biomarker used for immune checkpoint inhibitors alone or in combination with chemotherapy in NSCLC. Extracellular vesicles (EVs) were described as biomarkers in cancer and as modulators of anticancer immune response. In this study, we evaluated EVs of advanced NSCLC patients with low PD-L1 expression to find biomarkers for combinational therapy and to investigate their role as regulators of antitumor immune response.

**Material and Methods:** Plasma EVs from patients were characterized following MISEV guidelines. The tetraspanin profile was assessed via super-resolution microscopy. MiRNAs within EVs were evaluated using miRCURY LNA miRNA Focus Panel. Proteomics analysis on EVs was done by Tymora Analytical. EV subpopulations were sorted through FACS or immunocapture beads. Activated T cells cultured with different EV subpopulations were analyzed by flow cytometry.

**Results:** R- and NR-EVs were similar in terms of size and concentration. WB confirmed the presence of CD9, CD81, Tsg101, and HDL contaminants. Super-resolution microscopy revealed a slight increase in CD9+ EVs in NR. We found 9 miRNAs differentially expressed between the two groups and proteomics analysis revealed high levels of CD9, integrins, and adhesion molecules. Moreover, 132 proteins changed after immunotherapy. We isolated EV subpopulations based on their expression of CD9 and CD45. In functional experiments, CD9-CD45- EVs affected the percentage and Granzyme B production of CD4+ and CD8+ T cells.

**Conclusions:** R and NR EVs differ in terms of 9 miRNAs. Further analyses will identify specific proteins differentially present in EVs. Further studies will elucidate the role of EV subpopulations in immune response modulation in lung cancer.

## 46. The influence of interferons on extracellular vesicles produced by primary monocyte-derived macrophages

Flavia Giannessi^1,2^, Valentina Lombardi^1^, Zulema A. Percario^1^, Andrea Sabatini^1^, Alessandra Sacchi^1^, Luca Battistini^2^, Giovanna Borsellino^2^, Daniela F. Angelini^2^, Elisabetta Affabris^1^

^1^Department of Science, Rome Tre University, Rome, Italy.

^2^Neuroimmunology Unit, Santa Lucia Foundation IRCCS, Rome, Italy.

Cellular response to pathogens influences the production of cytokines, chemokines, and EVs. Interferons (IFNs) are fundamental effectors of antimicrobial innate immunity and important regulators of the adaptive immune response. The aim of this work was to analyze whether type I (IFNα2b and IFNβ), type II (IFNγ), and type III (IFNλ) IFNs can influence the composition of the EVs. As a cellular model, we used primary monocyte-derived macrophages (MDM) of healthy donors differentiated using GM-CSF (Granulocyte-Macrophage-Colony-Stimulating-Factor). Small-EVs (sEVs) were purified by size exclusion chromatography (SEC) from supernatants of cells treated for 20 h. To characterize concentration and dimensions, the vesicles were analyzed with Nanoparticle Tracking Analysis (NTA). sEVs surface markers were examined by a new flow cytometric multiplex bead-based platform (MacsPlex exosomes human kit) to evaluate the expression level of 37 different membrane proteins. Although no significant changes in the number or size of sEVs were observed, IFN treatments induce a significant down-regulation of CD9, CD63, and CD81 exosomal markers on sEVs. In addition, IFN-EVs showed a significant modulation of some adhesion molecules, co-stimulatory protein, and major histocompatibility complexes, suggesting sEVs participate in IFNs mediate the immune response. Experiments of treatment on autologous PBL with MDM-IFNs-sEVs are in progress to evaluate the impact of vesicles on T and B lymphocyte response. Altogether, our results show that EVs are affected by the IFN treatments, which can alter their “message”, inducing different outcomes in target cells.

## 47. DNA: RNA hybrids accumulate in the cytoplasm of senescent endothelial cells and are released through extracellular vesicles

Angelica Giuliani^1^, Deborah Ramini^2^, Jacopo Sabbatinelli^1,3^, Michele Guescini^4^, Gianluca Storci^5^, Spartaco Santi^6,7^, Massimiliano Bonafè^8^, Fabiola Olivieri^1,2^

^1^Department of Clinical and Molecular Sciences, Università Politecnica Delle Marche, Ancona, Italy.

^2^Clinic of Laboratory and Precision Medicine, IRCCS INRCA, Ancona, Italy.

^3^Laboratory Medicine Unit, Azienda Ospedaliera Universitaria "Ospedali Riuniti", Ancona, Italy.

^4^Department of Biomolecular Sciences, Università di Urbino Carlo Bo, Urbino, Italy.

^5^IRCCS Azienda Ospedaliero-Universitaria di Bologna, Bologna, Italy.

^6^IRCCS, Istituto Ortopedico Rizzoli, Bologna, Italy.

^7^Consiglio Nazionale delle Ricerche (CNR) Institute of Molecular Genetics "Luigi Luca Cavalli-Sforza", Bologna, Italy.

^8^Department of Experimental, Diagnostic and Specialty Medicine, Università of Bologna, Bologna, Italy.

Cellular senescence is a state of irreversible cell growth arrest in which cells remain metabolically active and acquire a stereotyped Senescence-Associated Secretory Phenotype characterized by the release of a variety of modulators, including extracellular vesicles (EVs). During senescence, molecular, physiological, and compartmental assets of cells are perturbed. DNA fragments, various species of RNAs, and DNA:RNA hybrids accumulate in the cytoplasm of senescent cells. These misplaced endogenous nucleic acids (NAs) can be sensed by receptors of the innate immune system, which normally recognize nucleic acids of bacteria and viruses. However, the role of these misplaced NCs in cells is still debated.

Here, we showed an increased abundance of NAs in the cytoplasm of senescent Human Umbilical Vein Endothelial Cells (HUVECs) compared to their non-senescent counterparts. Moreover, we isolated EVs of senescent and non-senescent cells through ultracentrifugation and characterized their surface epitopes using flow cytometry (MACSPlex). We found that senescent HUVECs released a significantly increased number of EVs evaluated with Nanoparticle Tracking Assay compared to young cells. EVs released by senescent endothelial cells showed pro-inflammatory/prosenescent features: they induce IL-6 release in THP-1/monocyte cells and growth arrest in proliferating HUVECs. Finally, we demonstrated an enhanced release of DNA:RNA hybrids in EVs by Transmission electron microscopy evaluation of S9.6 immunogold labeling.

Overall, our data suggest that cytoplasmatic accumulation of NAs in senescent endothelial cells represents an innovative marker of cellular senescence and that senescence could be spread to neighboring cells through EVs, at least in part through the action of such misplaced NAs.

## 48. Characterization of urinary evs isolated with different purification methods using single ev techniques

Cristina Grange^1^, Diego Prudente^2^, Benedetta Bussolati^2^

^1^Department of Medical Sciences, University of Turin, Turin, Italy.

^2^Department of Molecular Biotechnology and Health Sciences, University of Turin, Turin, Italy.

Extracellular vesicles (EVs), thanks to their complex cargo, appear to be ideal candidates as biomarkers of pathological processes, being relevant indicators in diagnostic and prognostic aspects. The use of new nanotechnologies such as super-resolution microscopy and nano-flow cytometry enables precise and accurate single EV evaluation. This has a major impact on the use of EVs for biomarker discovery. Urine is an ideal source of biomarkers, especially for kidney and urinary tract diseases, and it can be easily collected in large amounts without risk to the patient. A method that can efficiently isolate intact and pure EVs is the first step to exosome-based liquid biopsies.

Here, we compared different isolation methods to identify the best and most reliable protocols for markers’ evaluation at a single EV level, focusing on urinary EVs isolated from healthy subjects. In particular, we used six different protocols: four were based on ultracentrifugation, one implicated the use of SEC, and one utilized a commercial exosome precipitation reagent. Moreover, the use of a fixative to pretreat the clear urine to avoid immediate processing was also evaluated.

Urinary EVs isolated with different methods were analyzed using Super-Resolution Microscopy and nano-flow cytometer. Stem, regenerative, and renal markers were compared. uEV sub-populations were identified by the co-expression of renal markers from different nephron segments with the classical exosomal ones. In parallel, urinary EV yield, EV integrity and purity were also evaluated.

The analysis of marker expression on biofluid-EVs will be fundamental for the application of EVs as biomarkers for human pathologies.

## 49. Brain region specificity of astrocyte-derived extracellular vesicles: uncovering the mechanisms of neuroprotection in parkinson’s disease

Loredana Leggio^1^, Fabrizio Cavallaro^1^, Greta Paternò^1^, Maria Gaetana Giovanna Pittalà^2^, Sharon N. Cox^3^, Marco Falcone^1^, Marco Catania^1^, Mauro Distefano^1^, Ernesto Picardi^3^, Rosaria Saletti^2^, Nunzio Iraci^1^

^1^Department of Biomedical and Biotechnological Sciences, University of Catania, Catania, Italy.

^2^Department of Chemical Sciences, University of Catania, Catania, Italy.

^3^Department of Biosciences, Biotechnologies and Biopharmaceutics, University of Bari “Aldo Moro”, Bari, Italy.

Parkinson’s disease (PD) is a neurodegenerative disorder characterized by the progressive loss of dopaminergic neurons in the ventral midbrain (VMB) and their terminals in the striatum (STR). In this context, Astrocytes (AS) play an important role in the homeostatic regulation of dopaminergic neurons, with either detrimental or beneficial effects. However, the intercellular signaling between AS and neurons in PD has not been fully elucidated. Extracellular vesicles (EVs) have been proposed as possible mediators of this complex AS-neurons communication. We demonstrated that small EVs (100 nm) released by VMB- and STR-AS are able to protect cells from oxidative stress, and to recover the activity of mitochondrial complex I injured by MPP+ neurotoxin. Interestingly, only VMB-AS-EVs ameliorate ATP production, thus fully restoring the mitochondrial functionality. To shed light on the molecular mechanisms responsible for this region-specific neuroprotection of AS-EVs, we investigated (1) how and when AS-EVs enter target neurons, and (2) which are the AS-EV molecular cargoes. We found that target cells internalize AS-EVs over time, reaching a plateau after 48 h, and that EV uptake is mainly due to active endocytosis rather than membrane fusion. These data are supported by the GO Enrichment Analysis on EV proteins, which revealed the Integrin binding as one of the most representative Molecular Functions, thus suggesting the involvement of critical surface proteins in this process. Moreover, pathways related to mitochondrial activity are specifically enriched in VMB-AS-EVs. These results highlight novel AS-EV molecular candidates for the propagation of specific intercellular signaling with neuroprotective implications for PD.

## 50. Activin a modulates the microRNA cargo of extracellular vesicles released by B-cell precursor acute lymphoblastic leukemia (BCP-ALL) cells

Eugenia Licari^1^, Giulia Cricrì^1^, Francesca Raimondo^2^, Marina Pitto^2^, Silvia Bresolin^3^, Andrea Biondi^1,4^, Valentina Bollati^5^, Erica Dander^1^, Giovanna D'Amico^1^

^1^Tettamanti Center, Fondazione IRCCS San Gerardo dei Tintori, Monza, Italy.

^2^Clinical Proteomics and Metabolomics Unit, University of Milan-Bicocca, Milan, Italy.

^3^Women's and Children's Health Department, University of Padua, Padua, Italy.

^4^Pediatric Clinic, Fondazione IRCCS San Gerardo dei Tintori, Monza, Italy.

^5^EPIGET LAB, Department of Clinical Sciences and Community Health, Milan, Italy.

Activin A (ActA) exerts a pro-leukemic action by increasing BCP-ALL cell migration. Since Extracellular Vesicle (EVs) production is similarly dependent on cytoskeleton remodeling, we aimed to investigate the ActA effect on BCP-ALL vesiculation. After 24 h, ActA significantly increased the amount of small-EVs and large-EVs released by the BCP-ALL cell line 697 [Fold Change (FC) = 1.51, *P* < 0.008 and 1.31, *P* = 0.008, respectively, *n* = 10], but also modified EV miRNA cargo. Among deregulated miRNAs, ActA increased miR-491-5p of 1.5- and 3-folds in 697 cells and their EVs (*P* < 0.001). To study the role of miR-491-5p in BCP-ALL, we overexpressed it by transfecting 697 cells with a specific mimic. Interestingly, miR-491-5p promoted 697 cell proliferation up to 40% at day 4, compared to negative control transfected cells (*P* = 0.003, *n*= 7). Accordingly, EVs released from ActA-stimulated 697 cells significantly enhanced the proliferation of 697 cells, compared to EVs obtained from unstimulated cells (FC = 24% at day 3, *P* = 0.01, *n* = 4). Since miR-491-5p was described to mediate chemoprotection in solid tumors, we further investigated the ActA/miR-491-5p axis in Asparaginase (ASNase) resistance. Indeed, we stimulated or not 697 cells with ActA and then treated them with ASNase. Importantly, ActA+ASNase 697 cells exhibited increased viability (FC = 11,3%, *P* = 0.006, *n* = 3) and the highest level of miR-491-5p (FC = 2.5, *P* = 0.008, *n* = 8) compared to unstimulated cells. Accordingly, miR-491-5p downregulation by a specific inhibitor reduced 40% of ActA-mediated anti-apoptotic ability (*P* = 0.04, *n* = 3), possibly modulating the TP53 pathway (MiR-System database prediction). Indeed, miR491-5p inhibition resulted in a 5-8-fold increase in the mRNA expression of the pro-apoptotic molecule *TP53AIP1* (*P* = 0.03, *n* = 6). Overall, the ActA/miR-491-5p axis could represent a new target in BCP-ALL therapy.

## 51. Exercise-stimulated extracellular vesicles promote cardiomyocyte pro-survival programming in a redox-dependent manner

Veronica Lisi^1^, Giorgia Senesi^2,3^, Nadia Bertola^4^, Matteo Pecoraro^5^, Sara Bolis^6^, Alice Gualerzi^7^, Silvia Picciolini^7^, Andrea Raimondi^5,8^, Cristina Fantini^1^, Elisa Moretti^1^, Attilio Parisi^1^, Paolo Sgrò^1^, Luigi Di Luigi^1^, Roger Geiger^5^, Silvia Ravera^4^, Giuseppe Vassalli^2,3,9^, Daniela Caporossi^1^, Carolina Balbi^2,9^

^1^Department of Movement, Human and Health Sciences, University of Rome Foro Italico, Piazza Lauro de Bosis 15, Rome, Italy.

^2^Cellular and Molecular Cardiology, Istituto Cardiocentro Ticino, Laboratories for Translational Research, Ente Ospedaliero Cantonale, Bellinzona, Switzerland.

^3^Faculty of Biomedical Sciences, Università della Svizzera Italiana, Lugano, Switzerland.

^4^Department of Experimental Medicine, University of Genoa, Genova, Italy.

^5^Institute for Research in Biomedicine, Università della Svizzera italiana, Bellinzona, Switzerland.

^6^Cardiovascular Theranostics, Istituto Cardiocentro Ticino, Laboratories for Translational Research, Ente Ospedaliero Cantonale, Bellinzona, Switzerland.

^7^Laboratory of Nanomedicine and Clinical Biophotonics (LABION), IRCCS Fondazione Don Carlo Gnocchi, Milan, Italy.

^8^Centro Imaging Sperimentale, IRCCS Istituto Scientifico San Raffaele, Milan, Italy.

^9^Center for Molecular Cardiology, Zurich, Switzerland.

Physical exercise (PE) has been demonstrated to have beneficial effects on redox homeostasis maintenance. This is due to the stimulation of adaptive homeostasis in response to regular PE, adjusting antioxidant responses, and counteracting reactive oxygen species (ROS) increase that may contribute to the development of cardiovascular diseases (CVD). The mechanisms of such adaptations may involve inter-tissue communication through circulating extracellular vesicles (EVs). This study aimed to investigate the activity of AOEs, such as Catalase and Glutathione Reductase (GR), in plasma EVs collected from physically active healthy males before (EVs_Pre) and after (EVs_Post) a single endurance exercise (30’,70% HRmax). The study also measured the activity of AOEs in an *in vitro* model of human-induced-cardiomyocytes (hCM) treated with these EVs for 1 hour. The results showed a significant increase of Catalase and GR activity in POST_EVs (*P* < 0.0001), while ROS concentration decreased (*P* < 0.0001) compared to Pre_EVs. Additionally, Catalase and GR significantly increased in hCM treated with Post_EVs (*P* < 0.0001). Under the same treatment, lipid oxidation and ROS decreased compared to both untreated and PRE_EVs treated hCM. However, nuclear Nrf2 levels increased in hCM treated with both Pre_EVs and Post_EVs (*P* < 0.05). This study demonstrated that a single endurance exercise can modulate AOE activity in plasma EVs, impacting the redox homeostasis of hCM. The shuttle of AOEs and/or the increase of nuclear Nrf2 could decrease ROS, leading to potential cardioprotective effects. These findings support the idea that regular PE can be an effective strategy for maintaining redox homeostasis and potentially reducing the risk of CVD

## 52. C. jejuni CDT intoxicated-Caco-2 cells release EVs that inhibit proliferation in tumor intestinal epithelial and myeloid cells: potential utility for antitumor strategies

Daniele Lopez^1,2^, Barbara Canonico^1^, Mariele Montanari^1^, Michele Guescini^1^, Raffaella Campana^1^, Giovanna Panza^1^, Caterina Ciacci^1^, Francesca Luchetti^1^, Claudio Ortolani^1^, Stefano Papa^1^

^1^Department of Biomolecular Sciences, University of Urbino, Urbino, Italy.

^2^Department of Pure and Applied Sciences, University of Urbino, Urbino, Italy.

Extracellular vesicles (EVs) are spontaneously arising nanoparticles released by prokaryotic and eukaryotic cells. EVs have harvested significant interest in recent years due to their prominent role in intercellular communication; indeed, they transport not only host cellular content including proteins, DNA, RNA, and lipids, but also pathogen-derived molecules. Cytolethal Distending Toxins (CDTs) from different bacterial sources (C. jejuni, A. actinomycetemcomitans, and H. ducreyi) have been prospected as important molecules in the development of antitumor agents. This study aims to investigate the content of active CDT in EVs released by CaCo-2-treated cells. EVs produced by Caco-2 cells treated with C. jejuni ATCC 33291 lysate (EvA), C. jejuni 11168H cdtA mutant lysate (EvM), and by untreated control cells (EvC) were isolated by ultracentrifugation and studied by nanoparticle tracking analysis (NTA) and flow cytometry (FC). EVs labeled by PKH67 can be tracked by both FC and confocal microscopy (CM) after their administration to homologous Caco-2 and heterologous U937 cells. EV-induced effects reveal a moderate induction of apoptosis and alteration of autophagic features. Since the peculiar CDT-like effect is cell enlargement and G2/M cell cycle blocking, DNA content and cell cycle phases were evaluated in uninfected homologous Caco-2 and heterologous U937 cells, previously treated by EVs derived from infected Caco-2. In the current study, EVs represent affordable CDT carriers, which are particularly suitable for limiting the proliferation of colorectal adenocarcinoma Caco-2 cells. In conclusion, these data can support the setting up of potential bacterial-related bio-therapeutic tools in multidrug anticancer protocols.

## 53. Cancer-derived exosomal-alu RNA promotes colorectal cancer progression by inducing epithelial-to-mesenchymal transition through NLRP3 inflammasome activation

Sara Magliacane Trotta, Antonio Adinolfi Sandro De Falco, Valeria Tarallo 

Angiogenesis lab, Institute of Genetics and Biophysics ‘Adriano Buzzati-Traverso’, CNR, Naples, Italy.

Alu elements are among the most abundant interspersed repetitive elements (SINEs) in the human genome. It has been shown that Alu RNA accumulation is responsible for human diseases, such as geographic atrophy, one of the main causes of human blindness. This is the first evidence showing the involvement of Alu RNAs in a human disease. Interestingly, increased levels of Alu RNA expression have been observed in several cancers, although no correlation has yet been established. Furthermore, we demonstrated that Alu RNA accumulation induces epithelial-to-mesenchymal transition (EMT) and is associated with colorectal cancer (CRC) progression.

Here, we show that Alu RNA is able to induce EMT through NLRP3 inflammasome activation and the release of IL-1 in CRC cells. Moreover, Alu RNA is stored and transported by exosomes and can be transferred to CRC cells. Exosomal-Alu RNA induces tumorigenesis, promoting invasion, growth in an anchorage-independent manner, and metastasis. Corroborating this data, we found that the significantly increased expression of Alu RNA correlates with the induction of NLRP3 priming in human CRC patients. Furthermore, the expression level of Alu RNA from circulating exosomes correlates with CRC progression in the preclinical model.

These findings reveal the direct involvement of Alu RNA in cancer pathogenesis and their presence in CRC cell-derived exosomes could be used as a non-invasive diagnostic biomarker.

## 54. Amniotic fluid stem cell-derived extracellular vesicles reprogram type 2 conventional dendritic cells in experimental autoimmune encephalomyelitis

Giorgia Manni^1,7^, Marco Gargaro^1^, Simona Fontana^2^, Marco Cipolloni^3^, Tommaso Mazza^4^, Doriana Ricciuti^1^, Alessandro di Michele^5^, Giulia Mencarelli^1^, Benedetta Pieroni^1^, Francesco Sarnari^1^, Alessandra di Veroli^6^, Rita Romeni^1,7^, Francesca Fallarino^1,7^

^1^Department of Medicine and Surgery, University of Perugia, Perugia, Italy.

^2^Department of Biomedicine, Neuroscience and Advanced Diagnostic (Bi.N.D), School of Medicine and Surgery, University of Palermo, Palermo, Italy.

^3^TES Pharma S.r.l. Loc. Taverne Corciano, University of Perugia, Perugia, Italy.

^4^Laboratory of Bioinformatics, PI Fondazione IRCCS Casa Sollievo della Sofferenza, Rome, Italy.

^5^Department of Physics and Geology, University of Perugia, Perugia, Italy.

^6^Department of Chemistry, Biology and Biotechnology, University of Perugia, Perugia, Italy.

^7^Extracellular Vesicles network (EV-net) of the University of Perugia, Perugia, Italy.

Extracellular vesicles (EVs), released from human amniotic fluid stem cells (HAFSCs), have attractive attention due to their proliferative, regenerative, anti-inflammatory, and immunoregulatory properties. Dendritic cells (DCs) are potent antigen-presenting cells that control adaptive immunity and balance effector and regulatory components of the immune response.

In this study, we investigated the potential of HAFSC-EVs to promote tolerogenic effects on specific subsets of murine DCs. We demonstrated that HAFSC-EVs are preferentially internalized by conventional dendritic cell type 2 (cDC2) and only minimally by other cDCs, both *in vitro* and *in vivo*. Moreover, we found that HAFSC-EVs binding to cDC2 is mediated by ITGB1 integrin.

Analysis of protein and miRNA cargo revealed the enrichment of several immunoregulatory pathways in HAFSC-EVs. Indeed, inflammatory cDC2 conditioned with HAFSC-EVs acquired strong tolerogenic functions. Transfer of cDC2 conditioned with HAFSC-EVs *in vivo* resulted in the suppression of autoimmune responses and significant improvement in the clinical score of experimental autoimmune encephalomyelitis (EAE). These results demonstrate that HAFSC-EVs, which are naturally loaded with immunoregulatory mediators, contribute to reprogramming inflammatory cDC2 to tolerogenic functions, leading to the control of autoimmune responses.

## 55. Extracellular vesicles from adipose tissue-derived mesenchymal stRomel cells loaded with paclitaxel: isolation and characterization for future clinical use as an antitumor drug

Angela Marcianti^1^, Eleonora Spampinato^1^, Sara Nava^1^, Simona Frigerio^1^, Simona Pogliani^1^, Giulia Maria Stella^2^, Catia Traversari^1^, Paola Gagni^3^, Federico Cazzaniga^4^, Angelo Guido Corsico^2^, Daniela Lisini^1^

^1^Cell Therapy Production Unit, Scientific Direction, IRCCS Neurologic Institut C. Besta Foundation, Milan, Italy.

^2^Department of Medical Sciences and Infective Diseases, Unit of Respiratory Diseases, IRCCS Policlinico San Matteo Foundation and University of Pavia Medical School, Pavia, Italy.

^3^Scitec CNR, Milan, Italy.

^4^Division of Neurology 5 and Neuropathology, IRCCS Neurologic Institut C. Besta Foundation, Milan, Italy.

Extracellular vesicles (EVs) obtained from Mesenchymal StRomel Cells (MSCs) loaded with Paclitaxel (PTX) can represent innovative drugs to treat oncological diseases. The aims of this project were: (A) to assess the possibility of standardizing a Good Manufacturing Practice (GMP)-compliant protocol for the preparation of MSC-EVs loaded with PTX (MSC-PTX-EVs) obtained from lipoaspirated adipose tissue of healthy donors; (B) to characterize the EVs and PTX-EVs.

MSCs obtained from 9 donors were expanded under GMP conditions for a maximum of 3 passages. To obtain MSC-PTX-EVs, PTX (10 µg/mL) was added for 20 h to the culture medium before supernatant collection. EVs were isolated by supernatant ultracentrifugation (130 mL) and characterized in terms of concentration and size by Nanoparticle Tracking Analysis (NTA), protein content by microBCA, morphology by Transmission Electron Microscopy (TEM), and identity by flow cytometry analysis.

MSC-EVs and MSC-PTX-EVs are similar for concentration (9, 57E + 09 ± 7, 61E + 09 *vs*. 9, 36E + 09 ± 6, 34E + 09 particle/mL) and size distribution (205, 02 ± 13,69 nm *vs.* 207, 52 ± 10,82 nm). EV size/integrity were confirmed by TEM. Protein content was 81,77 ± 23,38 ng/mL *vs*. 108, 48 ± 34, 93 ng/mL. All data are reported as mean ± SD. Flow cytometry analysis showed high expression levels of EV markers CD9, CD81, and CD63, as well as MSC markers CD29, CD44, CD146, and CD105.

Studies to evaluate EV drug content, antitumor efficacy, and the possibility of scaling up the preparation process using a bioreactor are ongoing. Preliminary data demonstrate that our EV/EV-PTX preparation protocol is successful and permits maintaining product integrity/identity. These results indicate a possible future clinical use of EV-PTX as an antitumor drug.

## 56. Dissecting exosomal miRNAs as key players for the early identification of an aggressive subtype of lung adenocarcinoma

Francesco Mazzarelli^†^, Elisa Dama, Roberto Cuttano ,Rosa Maria Perrone, Patricia Kiptiu, Valentina Melocchi, Kuku Miriam Afanga, Tommaso Colangelo, Fabrizio Bianchi

Cancer Biomarkers Unit, Fondazione IRCCS Casa Sollievo della Sofferenza, San Giovanni Rotondo, Italy.

Non-Small Cell Lung cancer is the most frequently diagnosed lung cancer type (80%-90% of all cases) and lung adenocarcinoma (LUAD) is the major subtype (~ 40% of NSCLC). Treatment of lung cancer is difficult due to high pathological, cellular, and molecular heterogeneity. Therefore, gaining new knowledge about mechanisms that drive lung cancer progression is urgently needed. Recently, we discovered a tumor molecular subtype, namely C1-LUAD, using a 10-genes prognostic signature that correlates with a high-plasticity cell state, increased tumor mutational burden, immune evasion phenotype, and infaust prognosis. Non-invasive biomarkers for the early identification of C1-LUAD are needed. Coherently, we first investigated in plasma samples from LUAD patients a subset of exosomal-miRNAs (exo-miRs) that interact with the C1-LUAD transcriptome. We demonstrated that a model of 6-exo-miRs can accurately diagnose aggressive C1-LUAD tumors with an AUC = 0.73. We also found that C1-LUAD tumor cells are eager to internalize both self and exogenous exosomes compared with other tumor cells, thus suggesting a differential uptake kinetics. C1-LUAD cells also showed a peculiar exo-miRs profile, with miR-223-3p being the top significantly enriched in their exosomes but depleted in the intracellular compartment. Interestingly, when we re-expressed miR-223-3p in targeted C1-LUAD cells, we observed a reduction in cell migration and invasion. Notably, C1-LUAD exosomes enriched in miR-223-3p reduced T cell viability and proliferation, as well as IFN-gamma production. Overall, this study identifies a 6-exo-miRs signature for the early identification of aggressive lung cancer and provides insights into the role of exo-miRs in disease progression.

## 57. A novel prognostic biomarker signature reflected by large oncosomes is associated with aggressive prostate adenocarcinoma

Rossella Migliorino^1^, Chiara Ciardiello^1^, Domenico Mallardo^2^, Maria S. Roca^1^, Rita Lombardi^3^, Vincenzo Gigantino^4^, Giosuè Scognamiglio^4^, Carlo Vitagliano^1^, Biagio Pucci^1^, Tania Moccia^1^, Francesca Bruzzese^3^, Susan Costantini^1^, Marinella Pirozzi^5^, Maria Mangini^6^, Deniz Yilmaz^6^, Anna Chiara De Luca^6^, Michele Minopoli^7^, Paolo Antonio Ascierto^2^, Alessandra Leone^1^, Elena Di Gennaro^1^, Alfredo Budillon^8^

^1^Experimental Pharmacology Unit, Istituto Nazionale Tumori -IRCCS- Fondazione G. Pascale, Naples, Italy.

^2^Department of Melanoma, Cancer Immunotherapy and Development Therapeutics, Istituto Nazionale Tumori, -IRCCS-Fondazione G. Pascale, Naples, Italy.

^3^Animal Facility, Istituto Nazionale Tumori -IRCCS- Fondazione G. Pascale, Naples, Italy.

^4^Pathology Unit, Istituto Nazionale Tumori-IRCCS- Fondazione G. Pascale, Naples, Italy.

^5^Second Unit, Institute of Experimental Endocrinology and Oncology "G. Salvatore" (IEOS), National Research Council (CNR), Naples, Italy.

^6^Laboratory of Biophotonics and Advanced Microscopy, Second Unit, Institute of Experimental Endocrinology and Oncology "G. Salvatore" (IEOS), National Research Council (CNR), Naples, Italy.

^7^Preclinical Models of Tumor Progression Unit, Istituto Nazionale Tumori -IRCCS- Fondazione G. Pascale, Naples, Italy.

^8^Scientific Director, Istituto Nazionale Tumori -IRCCS- Fondazione G. Pascale, Naples, Italy.

Prostate Cancer (PCa) is one of the leading causes of cancer-related deaths among men around the world; advanced stages make the disease androgen-independent and more invasive, resulting in drug resistance. Patient stratification based on circulating prognostic biomarkers is desirable to guide treatment options. Taking advantage of PCa DU145 cells and their derived aggressive subline DU145R80, selected as resistant to mevalonate-pathway inhibitors, we demonstrated DU145R80 cells’ ability *in vivo* to promote distant engraftment of DU145 parental cells. Assuming a pivotal role of DU145R80-secreted factors in tumor-promoting activity, we characterized the molecular content of Extracellular Vesicles (EVs) from DU145R80 cells. Intriguingly, Large Oncosomes (LO) overexpressed both integrin αV and elongation factor1gamma (EEF1G) proteins, compared to small EVs exosomes (EXO). As we previously described for integrin-αV, here we showed a correlation between EEF1G expression and Gleason Score or lymph node status in PCa patients Tissue Microarray. We further investigated miRNA profiles in LOR80 compared to EXOR80. Among the 68 miRNAs upregulated in LOR80, we found that miR181a-5p, miR4454, miR93-5p, miR125a-5p, and let-7d-5p were overexpressed strongly in LOR80 compared to EXOR80. Targets of these miRNAs (LATS2, TP53, PTEN) are reported as key factors in PCa progression. Altogether, these data suggested that LO, reflecting aggressive PCa features, may be used as a reliable source of biomarkers for liquid biopsy, empowering the prognostic factors for PCa patients.

## 58. Involvement of the extracellular vesicles/macrophages/neurons axis in amyotrophic lateral sclerosis

Elisabetta Carata^#^, Marco Muci1^#^, Stefania Mariano, Elisa Panzarini

Department of Biological Sciences and Technologies, University of Salento, Lecce, Italy.

^#^Authors contributed equally.

Extracellular vesicles (EVs) can induce the polarization of macrophages in neuroinflammatory diseases, with consequent alteration of intercellular signal transduction and progression of diseases. The macrophages modulated by EVs can show two phenotypes: the pro-inflammatory M1 and the anti-inflammatory M2, and the polarization is induced by the cargo EVs. For this reason, the EVs/macrophage axis is a potential target for the study of neurodegenerative disease. Macrophage migration inhibitory factor (MIF) is a conserved cytokine involved in numerous processes: cytokine activity, receptor binding, and chemoattraction. MIF may play a protective or pathogenetic role in neurodegenerative disorders. In amyotrophic lateral sclerosis (ALS), MIF action reduces the aggregated misfolded SOD1. In this work, we explore whether EVs and MIF are functionally redundant or additive in regulating macrophage migration in terms of speed and directedness.

We cultured Raw 264.7 macrophages on the upper chamber of the 8 mm pore-size PET transwell and mSOD1 (G93A, A4V, G85R, G37R) NSC-34 cells in the lower chamber for 12, 24, and 48 h to evaluate the migration of macrophages and the inflammatory response of both macrophages and motor neurons. The results of co-culture show migration of macrophages in A4V and G93A mSOD1, due to an overexpression of IL8 released by NSC34 cells and by positive feedback of macrophages. Overall, the results of the present research highlight that macrophages have a high implication in the neuroinflammatory environment. A better understanding of the role of macrophages in amyotrophic lateral sclerosis could allow its use as a novel therapeutic target for monitoring neurodegeneration.

## 59. Surface functionalization of EVs with antibodies and the protein coronavariable

Angelo Musicò^1,2^, Rossella Zenatelli^1,2^, Miriam Romeno^1,2^, Andrea Zendrini^2^, Silvia Alacqua^1,2^, Selene Tassoni^1^, Lucia Paolini^2,3^, Chiara Urbinati^1^, Marco Rusnati^1^, Paolo Bergese^1,2^, Giuseppe Pomarico^1,2^, Annalisa Radeghieri^1,2^

^1^Department of Molecular and Translational Medicine, University of Brescia, Brescia, Italy.

^2^CSGI, Research Center for Colloids and Nanoscience, Florence, Italy.

^3^Department of Medical and Surgical Specialties, Radiological Sciences and Public Health, University of Brescia, Brescia, Italy.

Extracellular vesicles are soft bio-nanoparticles naturally released by cells for intercellular communication, which are to date in the spotlight as the “nanoparticles 2.0” for nanomedicine. A key strategy to improve extracellular vesicle (EV) targeting properties includes EV surface functionalization by covalently bonding tissue-specific protein ligands. However, up to date, works dealing with the functionalization of EV surface with proteins have never considered the protein corona “variable”, namely the fact that extrinsic proteins may spontaneously adsorb on the EV surface, contributing to determining the surface and, in turn, the biological identity of the EV. In this contribution, we show the comparison of the two edge cases of EVs functionalized with the antibody Cetuximab (CTX) by chemisorption (covalent binding of CTX via biorthogonal click-chemistry) and by physisorption (formation of a CTX corona). The two cases were mainly compared in terms of surface composition, morphology, mechanical properties, stability, on-chip affinity, and *in vitro* uptake, providing new biophysical and biological insights into a hot topic of the EV field, such as the protein corona and the EV surface functionalization.

## 60. Comparing EVs from solid and fluid human tissues: do’s and don’ts.

Paolini Lucia^1,2^, Mangolini Valentina^3,4^, Simone Piva^1,5^, Cattaneo Stefano^6^, Brucale Marco^2,7^, Valle Francesco^2,7^, Montis Costanza^2,8^, Federici Stefania^9^, Gazzina Stefano^10^, Guarneri Bruno^1^, Radeghieri Annalisa^2,3^, Latronico Nicola^1,5,11^, Paolo Bergese^2,3,12^

^1^Dept. of Medical and Surgical Specialties, Radiological Sciences and Public Health (DSMC), University of Brescia, Brescia, Italy.

^2^Center for Colloid and Surface Science (CSGI), University of Florence, Sesto Fiorentino, Florence, Italy.

^3^Dept. Molecular and Translational Medicine (DMMT), University of Brescia, Brescia, Italy.

^4^IRCCS Fondazione Don Carlo Gnocchi ONLUS, Milan, Italy.

^5^Dept. of Anesthesia, Critical Care and Emergency, ASST Spedali Civili, Brescia, Italy.

^6^Dept. of Bone and Joint Surgery, ASST Spedali Civili, Brescia, Italy.

^7^Istituto per lo Studio dei Materiali Nanostrutturati, National Research Council of Italy (CNR), Bologna, Italy.

^8^Dept. of Chemistry “Ugo Schiff”, University of Firenze, Firenze, Italy.

^9^Dept. of Mechanical and Industrial Engineering, University of Brescia and INSTM Unit of Brescia, Brescia, Italy.

^10^Dept. of Continuity of Care and Frailty, Neurology Unit, ASST Spedali Civili, Brescia, Italy.

^11^University Research Center on LOng Term Outcome (LOTO) in Survivors of Critical Illness, University of Brescia, Brescia, Italy.

^12^IRIB - Institute for Research and Biomedical Innovation of CNR, Palermo, Italy.

The majority of Extracellular Vesicle (EV) studies performed so far have focused on cell culture media or body fluid-derived EVs, due to the easy accessibility of the starting material and multiple protocols described in literature. A major unmet need in this field is represented by the separation and characterization of EVs from solid tissues. By separating EVs directly from solid tissue, we obtain a snapshot of the EVs in the microenvironment of release; furthermore, we have the potential to understand the mechanisms by which EVs, shed in the extracellular matrix of tissue, mediate a biological effect on their cellular targets, that can be located in different organs. This perspective is in its infancy due to technical challenges and the lack of rigorous approaches that allow comparing EVs of distinct origin, from solid and liquid tissues, minimizing bias due to different separation techniques and highlighting differences due to the EV microenvironments.

In this study, we present an optimized protocol to separate and analyze EVs both from human muscle tissue biopsy and plasma. The solid tissue protocol uses mechanic and enzymatic approaches and converges with the plasma protocol that combines serial (ultra)centrifugation and sucrose density gradient to separate different EV subpopulations. EV preparations have been analyzed using multidisciplinary techniques in terms of biophysical properties, purity from exogenous proteins and other nanoparticles, spectroscopic molecular fingerprint, and biochemical composition. Our results and practical tips will be of interest to all working on EV biogenesis, release, function, and EV-based biomarkers.

## 61. Small extracellular vesicles from human pluripotent stem cells: properties, miRNA/circRNA landscape and functions

Valeria Peli^1^, Mario Barilani^1^, Clelia Pistoni^1^, Francesco Rusconi^1^, Eva Maria Pinatel^2^, Francesca Pischiutta^3^, Alessandro Cherubini^1^, Dorian Tace^1^, Maria Chiara Iachini^1^, Vincenza Dolo^4^, Giovanna Damia^7^, Roberta Roncarati^9^, Beatrice Fontana^10^, Ilaria Pace^10^, Manuela Ferracin^10,11^, Elisa R Zanier^3^, Lorenza Lazzari^1^

^1^Cell Factory, Department of Transfusion Medicine and Hematology, Fondazione IRCCS Ca’ Granda Ospedale Maggiore Policlinico, Milan, Italy.

^2^ITB-CNR, Institute of Biomedical Technologies, National Research Council, Segrate, Italy.

^3^Laboratory of Acute Brain Injury and Therapeutic Strategies, Department of Neuroscience, Istituto di Ricerche Farmacologiche Mario Negri IRCCS, Milan, Italy.

^4^Department of Life, Health and Environmental Sciences, University of L’Aquila, L’Aquila, Italy.

^5^EPIGET Lab, Department of Clinical Sciences and Community Health, Università degli Studi di Milan, Milan, Italy.

^6^Department of Biotechnology and Biosciences, University of Milan-Bicocca, Milan, Italy.

^7^Laboratory of Molecular Pharmacology, Istituto di Ricerche Farmacologiche Mario Negri IRCCS, Milan, Italy.

^8^Laboratory of Transplant Immunology SC Trapianti Lombardia-NITp. Fondazione IRCCS Ca’ Granda Ospedale Maggiore Policlinico, Milan, Italy.

^9^Istituto di Genetica Molecolare “Luigi Luca Cavalli-Sforza- Consiglio” Nazionale delle Ricerche (CNR), Bologna, Italy.

^10^Department of Medical and Surgical Sciences (DIMEC), University of Bologna, Bologna, Italy.

^11^IRCCS Azienda Ospedaliero-Universitaria di Bologna, Bologna, Italy.

The goal of our study is to investigate an innovative cell-free therapy approach based on extracellular vesicles (EVs) isolated from human induced pluripotent stem cells (hiPSC), generated from clinical-grade HLA-typed cord blood mesenchymal stem/stRomel cells (MSCs), to reduce limitations of primary MSCs and generate an inexhaustible EV source.

hiPSC-EVs were isolated by serial centrifugations and purified by size-exclusion chRometography and sucrose density gradient. MISEV2018-compliant characterization was performed by NTA, western blot, flow cytometry, and electron microscopy. Biophysical features were compatible with small EV identity. Interestingly, these EVs did not express HLA class I and II. To investigate the RNA landscape in our EV, we focused our studies on miRNAs and circRNAs as possible novel players of the EV anti-inflammatory functions. To test the EV uptake and function, three different experimental approaches have been applied: we demonstrated an EV dose-dependent uptake by H19-7 rat neural cells; for the anti-inflammatory potential of EVs, the macrophage setting was used (RAW 264.7 macrophage cell line); and an *ex vivo* model of brain ischemia subjected to oxygen and glucose deprivation (OGD) was used to test whether our EVs could induce significant brain protection supporting proliferation of the injured tissue cells and blocking apoptosis. Interestingly, the biological role prediction indicated that the EV RNA cargos are involved in ischemic, hypoxic, and apoptotic brain-related processes. Very crucial, the full-length PGRN transcription factor absence and no subcutaneous teratoma formation indicate a non-teratogenic stemness-associated cargo.

Herein, we presented a novel EV drug with immunomodulatory properties for regenerative medicine.

Supported by Fondazione Regionale per la Ricerca Biomedica, Regione Lombardia, Project nr.CP_10/2018. ERP-2020-EuronanoMed3-NANO4STROKE, Project nr.ERP-2020-23680649.

## 62. Effect of environmental and lifestyle factors on circulating oncomiRs carried by extracellular vesicles in a population of subjects with overweight or obesity

Paola Monti^1^, Chiara Favero^1^, Luca Ferrari^1,2^, Laura Dioni^1^, Francesca Bianchi^3,4,^ Angela Cecilia Pesatori^1,2^, Elia Biganzoli^5^, Valentina Bollati^1,2^

^1^EPIGET LAB, Department of Clinical Sciences and Community Health, University of Milan, Milan, Italy.

^2^Occupational Health Unit, Fondazione IRCCS Ca' Granda Ospedale Maggiore Policlinico, Milan, Italy.

^3^Dipartimento di Scienze Biomediche per la Salute, University of Milan, Milan, Italy.

^4^U.O. Laboratorio Morfologia Umana Applicata, IRCCS Policlinico San Donato, Milan, Italy.

^5^Unit of Medical Statistics, Bioinformatics and Epidemiology, Department of Biomedical and Clinical Sciences (DIBIC), University of Milan, Milan, Italy.

Environmental and lifestyle factors sustain cancer etiopathogenesis and clinical outcomes; therefore, the identification of biomarkers of early drift from homeostasis could be of great importance for cancer prevention. This is particularly important for individuals with pre-existing hypersusceptibility conditions, such as those linked to adiposity. The molecular content of extracellular vesicles (EVs) can change in response to outer stimuli, either as an adaptive response or as a consequence of homeostasis loss. Therefore, dysregulation of EV-borne oncomiRs, i.e., miRNAs implied in cancer-related processes, might precede the clinical manifestation of cancer.

In this framework, we evaluated the possible association between multiple environmental/ lifestyle exposures and plasma levels of EV oncomiRs, which were quantified in 673 women and 238 men with Body Mass Index > 25 kg/m^2^ (SPHERE population). EVs were obtained by ultracentrifugation and the expression of 754 EV miRNA was analyzed by qPCR. Using the *OncomiR* database, we selected the top 50 oncomiRs for each of the three most common cancers in women (breast, colorectal, and lung carcinomas) and men (lung, prostate, and colorectal carcinomas). EV oncomiRs detected in > 20% of our populations were considered for statistical analysis. Our results highlight the presence of EV oncomiR clusters that respond similarly to certain environmental factors. Among them, the mean annual exposure to PM10 stands out for being associated with EV oncomiR changes in all tumor types. Future prospective studies are needed to investigate their potentiality as biomarkers for assessing risk factor susceptibility and performing better cancer risk stratification and preventive interventions.

## 63. Effects of intracellular pathway inhibitors on the secretion, protein, and lipid composition of fluorescent Bodipy sEV

Deborah Polignano^1^, Valeria Barreca^1^, Lorenzo Galli^1^, Francesco Bonanni^1^, Valentina Tirelli^2^, Massimo Sargiacomo^1^, Maria Luisa Fiani^1^

^1^National Center for the Global Health, Istituto Superiore di Sanità, Rome, Italy.

^2^Core Facilities Technical- Scientific Service, Istituto Superiore di Sanità, Rome, Italy.

Exosomes are small extracellular vesicles (sEV) formed within late endocytic compartments/multivesicular bodies (MVBs). Various machineries have been described to regulate their biogenesis, including the ESCRT machinery, the syntenin-ALIX pathway, tetraspanins, and lipids, but several aspects of these processes have not been fully elucidated. We developed a methodology to obtain fluorescent exosomes (Bodipy sEV) of endosomal origin by using Bodipy FL C16 (C16), a fluorescent palmitic acid that, upon internalization by cells, is converted into phospholipids that are incorporated into the bilayer of secreted vesicles. These can be directly quantified by flow cytometry (FC). To gain insight into exosome biogenesis, we combined this approach with the use of a panel of inhibitors of lipid metabolism and vesicular trafficking. Upon incubation of melanoma cells with C16 and selected inhibitors of cellular pathways, significant differences were observed in the secretion of Bodipy sEV as evaluated by FC and Nanoparticle Tracking Analysis, compared to the control cells. Interestingly, under all conditions, Bodipy sEV had the same relative distribution of tetraspanins (CD63, CD81, and CD9) as assessed by colocalization analysis. However, Western Blot analysis of sEV markers highlighted significant differences. Additionally, phospholipid analysis revealed differences that could be attributed to the different metabolisms of Bodipy lipids.

In summary, our results indicate that the use of inhibitors of intracellular pathways not only affects the secretion of sEV, but also their protein and lipid composition. This suggests that this approach has the potential to provide further insights into the process of sEV biogenesis.

## 64. Evaluation of extracellular vesicle-associated TGF-β as a biomarker in patients with endometriosis

Giuseppina Poppa^1^, Giulia Di Fazio^1^, Giulia Capanna^1,2^, Maurizio Guido^1,2^, Vincenza Dolo^1^, Ilaria Giusti^1^

^1^Department of Life, Health and Environmental Sciences, University of L’Aquila, Via Vetoio-Coppito II, L’Aquila, Italy.

^2^Obstetrics and Gynecology Unit, San Salvatore Hospital, L’Aquila, Italy.

Endometriosis is a common chronic gynecological disease characterized by the presence of endometrial-like tissue growing outside the uterine cavity. In many patients, it causes debilitating painful symptoms, thus worsening the quality of life and causing recourse to chronic medical therapy and, eventually, surgery. To date, the diagnostic gold standard remains minimally invasive laparoscopic surgery followed by histological confirmation, but extensive research aims to identify a non-invasive serological marker useful for diagnosis or prediction of the severity; in this regard, studies are also focusing on plasma extracellular vesicles (EVs).

This work aims to evaluate whether the EV-associated TGF-β (Transforming Growth Factor-β) can be used as a diagnostic biomarker for this pathology; TGF-β, indeed, appears to play a key role in the development of endometriosis.

To this end, 8 endometriosis patients and 8 controls were initially recruited, from whom blood samples were collected; the plasma EVs were isolated by differential centrifugation and ultracentrifugation. EVs were characterized by transmission electron microscopy (TEM), Nanoparticle Tracking Analysis (NTA), and western blot for the common EV-associated marker CD63. EV-associated TGF-β levels were quantified by ELISA.

The EVs characterization confirmed their identity; the NTA analysis did not highlight any differences either in terms of size or concentration between patients and controls. Nevertheless, the quantification of EV-associated TGF-β highlighted a trend towards higher levels in patients than controls.

Although the number of subjects analyzed was limited, these preliminary data suggest that, in endometriosis patients, EVs could be characterized by a higher TGF-β level than in healthy subjects, encouraging further studies.

## 65. Extrusion-based biotechnology for EV exogenous loading

Estella Rao^1,2^, Angela Paterna^1,2^, Giorgia Adamo^1,3^, Sabrina Picciotto^1,3,4^, Pamela Santonicola^5^, Valeria Longo^3^, Noemi Aloi^3^, Paola Gargano^1,3^, Daniele Romencino^1,3^, Rita Carrotta^2^, Samuele Raccosta^1,2^, Paolo Colombo^3^, Elia Di Schiavi^5^, Antonella Bongiovanni^1,3^, Mauro Manno^1,2^

^1^Cell-Tech HUB-CNR, Palermo, Italy.

^2^Institute of Biophysics (IBF)-CNR, Palermo, Italy.

^3^Institute for Research and Biomedical Innovation (IRIB)-CNR, Palermo, Italy.

^4^Department of Biological, Chemical and Pharmaceutical Sciences and Technologies, University of Palermo, Palermo, Italy.

^5^Institute of Biosciences and BioResources (IBBR), National Research Council (CNR), Naples, Italy.

Extracellular vesicles (EVs) have a high potential as drug delivery systems due to their intrinsic capability to vehicle biological materials and information. We have already performed the exogenous loading of different bioactive compounds by various methods (such as sonication, electroporation, *etc.*) into nanoalgosomes, which are EVs isolated from the marine microalgae Tetraselmis chuii by tangential flow filtration and thoroughly characterized by biochemical/biophysical analyses. Here, we set up a method for drug loading based on the perturbative extrusion technique, along with an accurate purification of loaded EVs by affinity or size exclusion chRometography. The mechanical stress applied by extrusion causes a destabilization of the vesicle membrane, promoting EV loading. We used both nanoalgosomes and EVs from HEK-293T cell cultures, loaded with a small fluorescent dye, to optimize the extrusion parameters and the loading efficiency. In addition, nanoalgosomes were specifically loaded with (1) a small drug known for its neuroprotective qualities and efficacy in rescuing animal models of spinal muscular atrophy, and (2) a recombinant allergen protein, whose shielding may pave new roads for immunotherapy. Depending on the cargo properties, different techniques were used to assess loading efficiency, including fluorescence, dot blot, and chemical extraction. In conclusion, we showed that nanoalgosomes, biocompatible, sustainable green EVs, are well-suited for transporting both small and large cargoes. Furthermore, we developed a biotechnological approach based on extrusion for efficient exogenous loading of drugs and proteins into extracellular vesicles.

## 66. Characterization of extracellular vesicles in cardiomyopathies

Alessandra Stefania Rizzuto^1^, Maria Francesca Greco^1^, Andrea Faggiano^2^, Marco Vicenzi^1,2^, Stefano Carugo^1,2^, Chiara Macchi^3^, Massimiliano Ruscica^2,3^

^1^Department of Clinical Sciences and Community Health, Università degli Studi di Milan, Milan, Italy.

^2^Department of Cardio-Thoracic-Vascular Area, Foundation IRCCS Ca' Granda Ospedale Maggiore Policlinico, Milan, Italy.

^3^Department of Pharmacological and Biomolecular Sciences, University of Milan, Milan, Italy.

Cardiomyopathies are a heterogeneous group of diseases characterized by ventricular hypertrophy (HCM) or dilatation (DCM) of the myocardium, leading to heart failure. The diverse aetiopathogenesis and the overlapping clinical features between these phenotypes represent challenges when a reliable biomarker must be used. Extracellular vesicles (EVs) are garnering interest, given their ability to carry bioactive cargo, which reflects the physiological and/or pathological state of the cell from which they derive. The aim was to characterize EV size, concentration, and origin in DCM (*n* = 12) and HCM (*n* = 15) patients.

**Results:** Patients with DCM (84% male) were 52 ± 11 (y) with a BMI of 27.4 ± 4 Kg/m^2^. Troponin was 21.,24 ± 16.8 µg/L. Plasma-derived EVs were isolated by size exclusion chRometography and were not contaminated with apo-B- and apo-A-containing lipoproteins (WB). EV concentration was 9.74*108 ± 1*108particles/mL and size was 175 ± 19.15 nm. Among EVs positive for CFSE (FACS analysis), 49.6% derived from monocytes (CD14+), 18.2% from macrophages (CD206+), 61% and 41.2% from endothelium (CD62E+ and CD202b+), 63% from cardiomyocytes (CD172a+), 54.9% from progenitor cells (CD309+) and 47% from platelets (CD41a+).

Patients with HCM (73% male) were 58 ± 9.2 (y) with a BMI of 26.4 ± 4.4 Kg/m^2^.Troponin was 39.25 ± 30.4 µg/L.EV concentration was 5.95*108 ± 4.8*108 particles/mL and size was 189 ± 31.7nm, 42.1% of CFSE stained-EVs derived from monocytes, 20.7% from macrophages, 55.9% and 36.7% from endothelial cells, 63.5% from cardiomyocytes, 40.7% from progenitor cells and 49% from platelets. EV-derived miRNAs affected the inflammatory cascade only in HCM (Gene Ontology analysis).

**Conclusions:** EV concentration and miRNA content can capture clinical differences in cardiomyopathies.

## 67. TDP-43 is a physiological EV cargo and its release is impaired in ALS pathology

Annamaria Nigro^1^, Roberto Furlan^2^, Nilo Riva^1^, Angelo Quattrini^1^, Alessandro Romeno^1^

^1^Neuropathology Unit, Institute of Experimental Neurology, San Raffaele Scientific Institute, Milan, Italy.

^2^Clinical Neuroimmunology Unit, Institute of Experimental Neurology, San Raffaele Scientific Institute, Milan, Italy.

Amyotrophic lateral sclerosis (ALS) is a fatal neurodegenerative disease characterized by the selective and progressive degeneration of upper and lower motor neurons. In > 95% of familial and sporadic ALS cases, the RNA/DNA-binding protein TDP-43 abnormally accumulates in phosphorylated/ubiquitinated inclusions in the cytoplasm of affected neurons and glia. Extracellular vesicles (EVs) are cell-derived vesicles that play roles in intercellular communication in physiological and pathological conditions. Several studies have demonstrated that EVs encapsulate and transport pathological TDP-43 protein, contributing to its spreading and neurodegeneration in ALS pathology. We investigated the physiological mechanisms underlying EVs’ constitutive release of TDP-43 and how this process is dysregulated in ALS pathogenesis. Human neuroblastoma cells and skin fibroblasts from healthy controls and ALS patients were used as cell models to investigate the TDP-43 released by EVs in physiological conditions and ALS disease. Large and small EVs were isolated using differential/density-gradient centrifugation protocols. Flow cytometry and western blot assays were used for EVs and TDP-43 cargo characterization. We demonstrated that (1) TDP-43 and phosphorylated TDP-43 are constitutively released through EVs in neuronal cells (SH-SY5Y); (2) TDP-43 isoforms are actual physiological EV cargoes in healthy fibroblasts; (3) the release of TDP-43 through EVs is impaired in the fibroblasts of ALS patients; (4) the EV biogenesis is altered in ALS fibroblasts. Our data provide new evidence that TDP-43 is a physiological cargo of EVs and may be involved as an RNA-binding protein in regulating the biogenesis of EVs in physiological conditions and ALS disease.

## 68. Fully artificial extracellular vesicles: a biomimicking strategy towards effective theranostic tools in nanomedicine

Giada Rosso, Valentina Cauda

Department of Applied Science and Technology, Politecnico di Turin, Turin, Italy.

Extracellular Vesicles (EVs) are nowadays of utmost interest in the nanomedicine field, being responsible for the delivery of key biomolecules and signaling moieties throughout the body with incredibly high efficiency and *in vivo* site-selectivity. For this reason, pristine, engineered EVs or other EV byproducts are employed as nano-sized theranostic tools. However, large-scale production of EVs at standardized and suitable clinical grade levels is very expensive and time-consuming. We, therefore, propose an innovative approach to design fully artificial EV-mimicking vesicles (EV-mimics) for theranostics scopes; in particular, we decided to mimic EVs produced by PC3 prostate cancer cell line, which have been demonstrated to have a strong homing capability towards bone tissue. Three different lipidic compositions were conceived with increasing complexity, starting from a consolidated liposomal formulation and getting gradually closer to the natural reference composition. This was achieved through a bottom-up approach, employing commercial lipids as building blocks, to obtain artificial lipid bilayers with controllable size, which can be further decorated with key molecules such as peptides to resemble their natural counterparts in terms of cargo delivery efficacy. EV-mimics were produced, experimentally characterized, and, in parallel, analyzed through coarse-grained molecular simulation methods, which provided fascinating insights into lipid organization and dynamics.

Although in its preliminary phases, the proposed approach has great promise in research and industrial fields. Indeed, EV-mimics can become a cost-effective, very powerful off-the-shelf theranostic product, with high reproducibility of morphological and *in vivo* functional properties, in compliance with regulatory standards.

## 69. Spheroids culture affects cellular senescence and increases the angiogenic potential of mesenchymal stRomel cells-derived secretome and extracellular vesicles

Matteo Rovere^1^, Daniele Reverberi^2^, Maria Elisabetta Palmalà^1^, Chiara Gentili^1^

^1^Department of Experimental Medicine, University of Genoa, Genoa, Italy.

^2^IRCCS Ospedale Policlinico San Martino, Genoa, Italy.

Secretion of paracrine factors and extracellular vesicles by mesenchymal stRomel cells gained considerable attention in the regenerative field for possible use as cell-free therapy. However, new culture methods, such as 3D culture, need to be introduced to better mimic the *in vivo* situation and avoid the rapid onset of cellular senescence that leads to the loss of the multipotency characteristics *in vitro* and a change in the secretome profile, normally composed of pro-angiogenic and immunomodulatory factors.

In this study, we demonstrated that, in MSCs, the diameter of the spheroids plays a central role in the onset of cellular senescence and the determination of the secretome composition. Spheroid diameter influences the generation of a hypoxic niche in the internal region of the spheroids, which increases cellular senescence in big spheroids (diameter ~ 300 μm), while in small spheroids (diameter ~ 200 μm), the hypoxic niche is absent, and the senescence decreases compared to 2D culture. It was observed that the type of culture has a drastic influence on secretome composition: in big spheroids, the secretome is enriched in pro-inflammatory cytokines, while in small spheroids, pro-angiogenic molecules. Analyzing the EVs, it was observed that 3D culture shows a higher release of EVs, which maintain characteristics similar to MSCs-EVs from 2D. Soluble factors and EVs from small spheroids exhibit higher angiogenic potential in a HUVECs angiogenic assay with respect to the other conditions.

These data suggest a possible use of this culture method for the production of MSC-derived EVs enriched in pro-angiogenetic factors.

## 70. Small extracellular vesicles and interferons in U-373 astrocytoma cells line

Melissa Gionfriddo^1^, Flavia Giannessi^1,2^, Andrea Sabatini^1^, Alessandra Sacchi^1^ Zulema Antonia Percario^1^, Luca Battistini^2^, Giovanna Borsellino^2^, Daniela F. Angelini^2^, Elisabetta Affabris^1^

^1^Department of Science, Rome Tre University, Rome, Italy.

^2^Neuroimmunology Unit, Santa Lucia Foundation IRCCS, Rome, Italy.

The central nervous system (CNS) can be triggered by various pathogens, including viruses. Neurons, astrocytes, and microglial cells are the major resident cells in CNS and are prepared to sense microbes and viruses, producing several factors such as cytokines, chemokines, and extracellular vesicles (EVs), which are increasingly highlighted as key players in intercellular communication during the immune response.

The goal of the present work was to investigate whether type I (i.e., IFNα2b and IFNβ), type II (IFNγ), and type III (IFNλ) interferons, as fundamental effectors of antimicrobial immunity, can influence the composition of the released EVs in the astrocytoma cell line U-373, as a model of human astrocytes. Cells were treated with IFNs for 20 hours, the supernatant was harvested and small EVs (sEVs) isolated. sEVs were numbered by Nanoparticles Tracking Analysis (NTA), revealing that none of the IFNs treatment changed the amount and dimension of the collected EVs. The vesicular cargo was investigated by a flow cytometric multiplex bead-based platform (MacsPlex exosomes human kit) for the evaluation of 37 different surface markers: we found the expression of the exosomal markers CD9/CD63, as well as CD44 and CD29 cell-to-cell interaction markers. Next, we observed the presence of IFITM1 and IFITM3 (two IFN-induced antiviral restriction factors) in sEVs cargo by western blot, suggesting an active role of sEVs in IFN induction pathways. Our results show that IFN treatments influence sEV content, possibly modifying their influence on target cells.

## 71. Exosomes released by UV-treated keratinocytes activate pDCs via TLR7: a model mechanism of type I interferon triggering in psoriasis

Valentina Salvi^1^, Carolina Gaudenzi^1^, Silvia Alacqua^1^, Silvano Sozzani^2,3^, Paolo Bergese^1^, Daniela Bosisio^1^

^1^Department of Molecular and Translational Medicine, University of Brescia, Brescia, Italy.

^2^Laboratory Affiliated to Istituto Pasteur Italia-Fondazione Cenci Bolognetti, Department of Molecular Medicine, Sapienza University of Rome, Rome, Italy.

^3^IRCCS Neuromed, Pozzilli, Italy.

Psoriasis is a chronic skin disease, systemic, immune-mediated, and non-contagious, affecting 2%-3% of the world population. Excessive production of type I interferons (IFN-I) by plasmacytoid dendritic cells (pDCs) is the crucial event responsible for the local activation of autoimmune T cells and tissue damage. Since we previously described that exosomal GU-rich microRNAs (miRNAs) activate pDCs by triggering TLR7 in systemic lupus erythematosus, we asked whether deregulated miRNA secretion by damaged keratinocytes may represent a new pathogenic mechanism of pDC activation in psoriasis. We found that several GU-miRNAs were upregulated in psoriatic skin lesions and in exosomes derived from UV-treated keratinocytes, used as a model of damaged cells in psoriasis. Exosomes from UV-treated cells stimulated the activation of pDCs in terms of IFN-I production via TLR7 triggering. Finally, pDCs treated with exosomes from UV-treated keratinocytes induced the activation of cytotoxic CD8+ T cells. These findings identify exosomal miRNAs as novel pathogenic mediators of pDC activation in the onset of psoriasis, setting the basis to identify new targets for therapeutic options in psoriasis.

## 72. C. elegans as an *in vivo* model to characterize EV bioactivity and biodistribution

Pamela Santonicola^1^, Rosa Mocerino^1^, Giorgia Adamo^2^, Sabrina Picciotto^2^, Andrea Zendrini^3^, Daniele P. Romencino^2^, Angela Paterna^5^, Estella Rao^5^, Samuele Raccosta^5^, Stella Frabetti^4^, Olivia Candini^4^, Nicolas Touzet^6^, Mauro Manno^5^, Annalisa Radeghieri^3^, Miriam Romeno^3^, Paolo Bergese^3^, Antonella Bongiovanni^2^, Elia Di Schiavi^1^

^1^Institute of Biosciences and BioResources (IBBR), National Research Council (CNR), Naples, Italy.

^2^Cell-Tech HUB and Institute for Research and Biomedical Innovation, National Research Council of Italy (CNR), Palermo, Italy.

^3^University of Brescia and CSGI, Brescia, Italy.

^4^Rigenerand, Modena, Italy.

^5^Cell-Tech HUB and Institute of Biophysics, National Research Council of Italy (CNR), Palermo, Italy.

^6^Atlantic Technological University, Sligo, Ireland.

Extracellular vesicles (EVs) are secreted by almost all cell types and can be successfully isolated from different biological sources. EVs carry intrinsic biological material for cell-cell communication and are one of the most promising cell-derived nanoparticles for drug delivery. For these reasons, the biological activity of EVs and tissue distribution need to be characterized *in vivo*. Caenorhabditis elegans is a unique model to study EVs *in vivo*, thanks to its powerful genetics, short life cycle, and low costs. Moreover, being an invertebrate, it raises less ethical issues and fulfills the 3Rs principles. We successfully used *C. elegans* to test *in vivo* different types of EVs, such as the one derived from microalgae (nanoalgosomes) or from mammalian cells (Mesenchymal Stem Cells, MSCs, and Red Blood Cells, RBCs). We showed that RBC EVs and nanoalgosomes improve animal movement, with the latter also showing antioxidant and anti-aging properties. Moreover, thanks to *C. elegans* body transparency, we visualized in a whole living animal the uptake of labeled EVs, and we could define a specific subcellular localization, tissue tropism, and timing of uptake for each of the investigated EV populations. Finally, we demonstrated through genetics the involvement of clathrin-mediated endocytosis as one of the internalization mechanisms, and we proved that *C. elegans* can be used as a potency assay to establish the quality and reproducibility of EV batches.

## 73. Extracellular vesicles from plasma and skeletal muscle of amyotrophic lateral sclerosis mice models induce differential metabolic alterations in recipient myotubes

Carolina Sbarigia^1^, Stefano Tacconi^2^, Simone Dinarelli^3^, Fratini Nicole^1^, Silvia Scaricamazza^4,5^, Cristiana Valle^4^, Rome Sophie^2^, Luciana Dini^1,6^

^1^Department of Biology and Biotechnology “C. Darwin”, University of Rome Sapienza, Rome, Italy.

^2^CarMeN Laboratory, Lyon-Sud Faculty of Medicine, LYON 1 University, Pierre Bénite, Lyon, France.

^3^Institute for the Structure of Matter (ISM), National Research Council (CNR), Rome, Italy.

^4^Fondazione Santa Lucia IRCCS, c/o CERC, Rome, Italy.

^5^Department of Biology, University of Rome Tor Vergata, Rome, Italy.

^6^Research Center for Nanotechnology for Engineering (CNIS), Sapienza University of Rome, Rome, Italy.

Amyotrophic Lateral Sclerosis (ALS) is a multisystem disorder in which extracellular vesicles (EVs) play a role in neurodegeneration and skeletal muscle alterations. We investigated the effects of circulating and skeletal muscle-derived EVs from SOD1-G93A ALS mice models on recipient myotubes. EVs from wild-type (WT) and SOD1-G93A mice at different stages of the disease (early and late, 90 and 120 days of age, respectively) were isolated from plasma and skeletal muscle (SkM) by ultrafiltration combined with size exclusion chRometography (UF/SEC), and characterized by Transmission Electron Microscopy (TEM), cryo-TEM, Atomic Force Microscopy (AFM) and EV-markers expression. To assess the contribution of circulating and SkM SOD1-G93A EVs in damage spreading, C2C12 myotubes were treated with EVs for 24h and detrimental effects were evaluated in terms of metabolic alterations and oxidative stress.TEM and AFM morphological analysis of circulating and SkM-derived EVs revealed the presence of round-shaped EVs, with differences in size and number between WT and SOD1-G93A. Furthermore, the expression of CD63 and CD9 confirmed the presence of EVs in the isolated fractions. The treatment of C2C12 myotubes with circulating and SkM SOD1-G93A EVs (from 90 and 120 days) induced differential alterations in the AMP-activated protein kinase (AMPK) pathway, mitochondrial function, and antioxidant activity. Our results may provide interesting perspectives on the effects of circulating and SkM-derived EVs on recipient myotubes during disease progression, highlighting a different contribution of the systemic and local EVs crosstalk in ALS skeletal muscle pathogenesis.

## 74. Extracellular vesicles isolated from human plasma as a potential diagnostic and prognostic tool in burn septic shock patients

Martina Schiavello^1^, Barbara Vizio^1^, Filippo Mariano^1^, Stefania Bruno^1^, Ornella Bosco^1^, Anna Pensa^2^, Daniela Risso^2^, Giovanni Camussi^1^, Giuseppe Montrucchio^1^, Enrico Lupia^1^

^1^Department of Medical Sciences, University of Turin, Turin, Italy.

^2^Burn Centre, CTO Hospital, A.O.U. Città della Salute e della Scienza, Turin, Italy.

**Introduction:** Extracellular vesicles (EVs) represent promising non-invasive biomarkers that may aid in the diagnosis and risk stratification of septic shock in burn patients. Here, we investigated the value of EVs in septic shock, which remains the main cause of death in burn patients.

**Methods:** We enrolled twenty-nine burn patients, including burn-septic shock patients (BSP, *n* = 23) and burn non-septic patients (BnSP, *n* = 6). Ten healthy subjects (HS) were used as controls. Plasma-derived EVs were isolated by ultracentrifugation and characterized following the MISEV 2018 guidelines by nanoparticle tracking analysis, flow cytometry, and transmission electron microscopy (TEM). EV surface antigens were investigated by bead-based multiplex flow cytometry. *In vitro*, we reproduced the effects of EVs on platelet function by adding EVs from BSP or HS to platelet-rich plasma by healthy donors.

**Results:** Plasma-derived EVs were successfully isolated and their presence was confirmed by TEM and flow cytometry analyses of small EVs-tetraspanin markers that showed enrichment in CD63 and CD9. When compared to controls, BSP exhibited increased plasma-derived EV concentration. BSP EV phenotyping revealed a pattern of cell surface markers associated with septic shock and identified platelets, leucocytes, lymphocytes, and endothelial cells as EVs’ parental cells. We found a significant increase of CD42a that discriminated BSP from HS and BnSP. Moreover, EVs isolated from septic shock patients primed platelet aggregation.

**Summary/Conclusions:** Plasma-derived EVs are potentially related to septic shock in burn patients. In particular, septic shock in burn patients might be reflected in a pool of heterogeneous circulating EVs that contribute to inflammatory-related signaling mechanisms.

## 75. Human cardiomyocyte-derived extracellular vesicles regulate cardiac fibroblast activation through MIR-24

Giorgia Senesi^1,2,3^, Alessandra M. Lodrini^4^, Davide Ceresa^5^, Sara Bolis^1,6^, Paolo Malatesta^5,7^, Marie-José Goumans^4^, Carolina Balbi^1,2,8^, Giuseppe Vassalli^1,2,3,8^

^1^Cellular and Molecular Cardiology Lab, Istituto Cardiocentro Ticino-EOC, Bellinzona, Switzerland.

^2^Laboratories for Translational Research, EOC, Bellinzona, Switzerland.

^3^Faculty of Biomedical Sciences, Università della Svizzera Italiana, Lugano, Switzerland.

^4^Department of Cell and Chemical Biology, Leiden University Medical Center, Leiden, the Netherlands.

^5^Cellular Oncology Unit, IRCCS Ospedale Policlinico San Martino, Genova, Italy.

^6^Laboratory for Cardiovascular Theranostics, Istituto Cardiocentro Ticino-EOC, Bellinzona, Switzerland.

^7^Experimental Biology Unit, Department of Experimental Medicine (DIMES), University of Genova. Genova, Italy.

^8^Center for Molecular Cardiology, Zurich, Switzerland.

**Introduction:** Cardiac fibrosis involves the increased deposition of collagen type I and activation of cardiac fibroblasts (CF) into myofibroblasts. The contributions of the microenvironment to CF activation are not well understood. Extracellular vesicles (EVs) are nanoparticles released by cells, carrying proteins, lipids, and nucleic acids, which modify the cellular activity of recipient cells. Here, we aim to identify the role of EVs derived from cardiomyocytes (CM) on CF cellular activity in healthy and pathological (MI) conditions, focusing on miR24 activity.

**Methods:** In silico analysis identified miR24 as a putative miRNA with a role in the regulation of CF activation and proliferation. The investigation of miR's role in human CF was assessed following direct miR24 transfection by immunofluorescence and western blot analysis. miR24 presence in human iPS-CM derived-sEV was confirmed by RealTime-PCR.

**Results:** miR24 transfection on hCF demonstrated the role of this miR in the maintenance of the quiescent status of hCF, reducing proliferation and activation mediated by TGFβ treatment. hCM-sEV recapitulated miR transfection, while treatment with sEV derived from cardiomyocytes damaged with hypoxia (MI-hCM) treatment resulted in less efficiency. Interestingly, MI-hCM-sEV showed lower levels of miR24, suggesting the pivotal role of these miRs.

**Conclusion:** These data demonstrate the role of hCM-sEV in the regulation of hCF, possibly through miR-24. Further analysis, including the use of a 3D beating human heart organoid, will help to understand the hCM-hCF’s crosstalk.

## 76. Neural-derived extracellular vesicles and their non-coding RNAS (ncRNAS) cargo in frontotemporal dementia and bipolar disorder: an epigenetic approach useful for a differential diagnosis

Maria Serpente^1^, Chiara Fenoglio^2^, Andrea Arighi1, Emanuela Rotondo^1^, Caterina Visconte^2^, Marina Arcaro^1^, Giorgio Bocca^1^, Giuseppe Delvecchio^1^, Lorena Di Consoli^1^, Adele Ferro^1^, Cecilia Prunas^1^, Antonio Callari^1^, Paolo Brambilla^1,2^, Daniela Galimberti D.^3^

^1^Fondazione IRCSS Ca’ Granda, Ospedale Maggiore Policlinico, Milan, Italy.

^2^University of Milan, Dept of Pathophysiology and Transplantation, Milan, Italy.

^3^University of Milan, Dept. of Biomedical, Surgical and Dental Sciences, Centro Dino Ferrari, Milan, Italy.

Many studies suggested a link between Bipolar Disorder (BD) and behavioral variant Frontotemporal Dementia (bvFTD), but the specific neurobiological signatures characterizing these two disorders are still unclear. NcRNAs (miRNAs and long non-coding RNAs) are regulatory RNAs involved in several cellular processes and enriched in extracellular vesicles (EVs). EVs can be retrieved in several body fluids. The aim of this study was to analyze the ncRNAs cargo of neural-derived (ND)EVs in plasma from bvFTD, BD patients, and healthy controls (HC) in order to identify specific expression patterns. We isolated NDEVs from 20 bvFTD, 40 BD (20 early onset BD, 20 late onset BD) patients, and 20 HC by immunoprecipitation with anti-L1CAM antibody. Real-Time PCR analysis was performed for ncRNA gene expression analysis. We observed the expression of 200 miRNAs in NDEVs and a specific signature was observed for all groups of patients. In particular, miR-126-5p, miR-92a, and miR-106a were statistically deregulated in bvFTD and LOBD (*P* = 0.0253; *P* = 0.0188; *P* = 0.0023), whereas miR-34a and miR-520f only in BD patients (*P* = 0.0067; *P* = 0.0044). We observed, also, the expression of 30 lncRNAs, but only HOTAIR, BACE1-AS, NEAT1 and ANRIL were deregulated in bvFTD patients, reaching the statical significance (*P* = 0.035; *P* = 0.001; *P* = 0.021; *P* = 0.001). In conclusion, we discovered a deregulation of ncRNA expression levels specific to each patient group. These exploratory data could be useful in underpinning the neurobiology underlying bvFTD and BD. Nevertheless, further studies are required to identify a specific epigenetic signature that could aid in a differential diagnosis.

## 77. MicroRNAs loaded in circulating small neural-derived extracellular vesicles: potential biomarkers in early diagnosis of alzheimer's disease

Tatiana Spadoni^1^, Angelica Giuliani^2^, Marica Pagliarini^3^, Michele Guescini^3^, Patrizia Ambrogini^3^, Anna Rita Bonfigli^4^, Giuseppe Pelliccioni^5^, Maria Cristina Albertini^3^, Laura Graciotti^1^, Fabiola Olivieri^2,6^

^1^Department of Biomedical Sciences and Public Health, Università Politecnica delle Marche, Ancona, Italy.

^2^Department of Clinical and Molecular Sciences, Università Politecnica delle Marche, Ancona, Italy.

^3^Department of Biomolecular Sciences, University of Urbino “Carlo Bo”, Urbino, Italy.

^4^Scientific Direction, IRCCS INRCA, Ancona, Italy.

^5^ Neurology Department, IRCCS INRCA, Ancona, Italy.

^6^Center of Clinical Pathology and Innovative Therapy, IRCCS INRCA.

Alzheimer’s disease (AD) is a progressive neurodegenerative disease characterized by decreased memory and cognitive functions. Nowadays, AD still lacks minimally invasive circulating biomarkers that could facilitate the diagnosis and the monitoring of disease progression.

MicroRNAs (miRNAs) are emerging as tissue-specific and/or circulating biomarkers of several age-related and inflammatory diseases, but evidence on AD is still not conclusive. Usually, circulating miRNAs are encapsulated in membrane vesicles or are released from apoptotic bodies. Cells from the central nervous system can secrete small extracellular vesicles (EVs), both in physiological and pathological conditions. These small EVs could thus reflect the state of the neural cells, possibly contributing to the diagnosis of neurodegenerative disease.

## 78. Study of isolated plasma extracellular vesicles bearing SARS-COV-2 nucleoprotein of Covid-19 patients and their roles in the disease progression

Serena Toffanin, Giorgia Nuozzi, Claudia Maria Radu, Elena Campello, Cristiana Bulato, Paolo Simioni

Thrombotic and Hemorrhagic Diseases Unit Department of Medicine – DIMED University of Padua, Padua, Italy.

**Background:** A hypercoagulable state is commonly considered a major component of the pathophysiology of the infectious disease COVID-19. Extracellular vesicles (EVs), originating from cytoplasmic membranes following cell activation or apoptosis, can be identified by cell-specific surface markers. COVID-19 patients show many pathophysiological processes associated with the cellular release of EVs, including endothelial injury, platelet activation, TF-mediated procoagulant activity, and increased thrombin generation. In fact, COVID-19 patients show a significantly higher number of EVs than controls.

**Aims:** To isolate, by CytoFLEX-SRT cytometry, circulating plasma EVs expressing Sars-CoV-2-Nucleoprotein (NP) from COVID-19 patients and evaluate their *in vitro* capability to activate human endothelial cells.

**Methods:** 100 µL plasma from COVID-19 patients was stained with calcein-AM and Sars-CoV-2-NP. The double positive EVs, with a range of 70-200 nm diameter, were isolated by CytoFLEX-SRT cytometry. Human coronary endothelial cells were treated with the isolated EVs and analyzed for the ability to endocytose EVs using confocal microscopy. To evaluate the endothelial cell activation by EVs, the expression of tissue factor was investigated.

**Results:** Human endothelial cells are able to internalize Sars-CoV-2-NP+ and calcein-AM+ EVs isolated from COVID-19 patients’ plasma. Endothelial activation is confirmed by the increased expression of tissue factors.

**Discussion:** These results suggest that EVs are involved in cell-cell communication and play an important role as virus protein vehicles. Further studies are needed to define the exact mechanism by which the different EV subtypes play a role in the pathogenesis of COVID-19 coagulopathy.

## 79. From bench to bed translation of extracellular vesicles-based pharmaceuticals: a comparative study of different preservation methods

Francesca Susa^1^, Tania Limongi^1^, Francesca Borgione^1^, Silvia Peiretti^1^, Marta Vallino^2^, Valentina Cauda^1^, Roberto Pisano^1^

^1^Department of Applied Science and Technology (DISAT), Politecnico di Turin, Turin, Italy.

^2^Consiglio Nazionale delle Ricerche (CNR) di Turin, Turin, Italy.

In recent years, extracellular vesicles (EVs) have been deeply investigated as crucial intercellular communication mediators and key players in many physiological and pathological conditions. These features have been exploited for the development of new diagnostic tools and innovative therapeutic agents.

Despite EVs’ increasing use in research, the standardization of their reliable and reproducible storage methods, simplifying handling and transportation and maintaining intrinsic physical and biochemical characteristics, is still needed. To date, the only effective storage method is freezing at -80 °C, introducing high costs and limits in transportability and availability for therapeutic applications. To fill this gap, in this study, we compared the effectiveness of different storage methods, including freeze-drying, and formulations to preserve B-lymphocytes-derived EVs morpho-functional features, i.e., concentration, size, protein content, and surface antigen expression.

To develop a preservation method that minimally affects EVs' integrity and functionality, we combined the freeze-drying process with different excipients, some already known in the pharmaceutical field, while others used for the first time, such as cell culture media, human serum, and secretome. After lyophilization, some of them, i.e., human serum, cell culture media, secretome, and sugars with dextran and glycine, successfully preserved EVs’ features. We evaluated reconstituted EVs’ cytotoxicity and internalization in healthy (B-lymphocytes) and tumoral (Burkitt's lymphoma) cells to assess the maintenance of EVs' biological activity. They demonstrated statistically significant toxicity only towards cancerous cells, opening new therapeutic perspectives in the oncological field. In addition, results showed that biological or cellular-conditioned fluids can act as cryoprotectants but also as an active pharmaceutical ingredient, tuning EVs’ therapeutic effect and increasing their cellular internalization.

## 80. Looking for potential acute kidney injury biomarkers on the urinary extracellular vesicle surface

Adele Tanzi^1^, Sarah Tassinari^1^, Cristina Grange^2^, Valentina Bettio^3,4^, Daniela Capello^3,4^, Vincenzo Cantaluppi^5^, Benedetta Bussolati^1^

^1^Department of Molecular Biotechnology and Health Sciences, University of Turin, Turin, Italy.

^2^Department of Medical Science, University of Turin, Turin, Italy.

^3^UPO Biobank, University of Piemonte Orientale, Novara, Italy.

^4^Department of Translational Medicine, University of Piemonte Orientale, Novara, Italy.

^5^Nephrology and Kidney Transplant Unit, Department of Translational Medicine (DIMET), University of Piemonte Orientale (UPO), Novara, Italy.

Acute kidney injury (AKI) is defined as a sudden loss of excretory kidney function, which occurs in approximately 20% of hospitalized patients and represents a risk factor for chronic kidney disease development. More efficient diagnostic and prognostic biomarkers are needed to anticipate the diagnosis and monitor the progression of disease. Urinary extracellular vesicles (uEVs) have been shown to be a promising source of potential biomarkers for kidney diseases. The aim of the present study is to identify potential AKI biomarkers within urine and, in particular, on the uEV surface. Additionally, we aim to set up a method that can be easily translated into the clinic, avoiding the necessity to isolate uEVs. AKI patients’ urine samples were kindly provided by biobank UPO (Novara, Italy), who had previously collected and stored them at -80 °C. The present study included patients who developed AKI during hospitalization (*n* = 31) and patients who developed AKI after kidney transplantation (KT, *n* = 36). As controls, we include in the study 15 healthy subjects, 4 non-AKI hospitalized patients, and 28 non-AKI kidney transplanted patients. For all the samples, the expression of 37 surface antigens was characterized by flow cytometry using the MACSPlex Exosome Kit. Preliminary data showed significant differences in the expression level of some markers, such as CD24, HLA-DR, and CD44, comparing hospitalized AKI patients with healthy controls. The analysis of KT-AKI samples is still ongoing. Finally, the correlation of marker expression with functional and clinical parameters will be performed. This study may pave the road for the identification of new AKI biomarkers.

## 81. Colorectal cancer EV ID-card: surface profile of vesicles extracted from different human crc tissues

Sarah Tassinari^1^, Edoardo D’Angelo^2,3^, Federico Caicci^4^, Jacopo Burrello, Alessandro Musso^5^, Giuseppe Giraudo^6^, Marco Ettore Allaix^6^, Giorgio Maria Saracco^1,6^, Mario Morino^6^, Paola Cassoni^1^, Marco Agostini^2,3^, Federica Collino^7,8^, Benedetta Bussolati^1^

^1^Department of Medical Sciences, University of Turin, Turin, Italy.

^2^First Surgical Clinic, Department of Surgery, Oncology and Gastroenterology, University of Padua, Padua, Italy.

^3^Institute of Pediatric Research, Fondazione Città della Speranza, Padua, Italy.

^4^Department of Biomedical Sciences, University of Padua, Padua, Italy

^5^Division of Gastroenterology, A.O.U. Città della salute e della scienza, Molinette Hospital, Turin, Italy.

^6^Department of Surgical Sciences, University of Turin, Turin, Italy.

^7^Laboratory of Translational Research in Paediatric Nephro-urology, Fondazione Ca' Granda IRCCS Ospedale Maggiore Policlinico, Milan, Italy.

^8^Department of Clinical Sciences and Community Health, University of Milan, Milan, Italy.

Extracellular vesicles (EVs) are important in tumor progression and diffusion; their characterization within the tumor microenvironment (TME) may represent a molecular fingerprint of the tumor. In this study, we aimed to characterize EVs from different sources in colorectal cancer (CRC) patients to identify possible biomarkers of tumor stages and progression.

EVs were extracted from 110 fresh tumors (CRC) and coupled with 10-cm far normal (HC) tissue, or 46 decellularized tissues (CRC-ECM) and normal (HC-ECM) tissue. EVs were extracted after enzymatic digestion and ultracentrifugation and analyzed by NTA, TEM, super-resolution microscopy, and bead-based cytofluorimetric analysis.

NTA and TEM confirmed the presence of intact EVs in our preparations. Tetraspanin expression was confirmed by super-resolution microscopy and FACS. Analysis of ECM-EVs showed a different marker distribution related to tumor stage, and a significant increase of immunological molecules in HC-ECM surrounding the tumor of metastatic patients. In addition, CD25 (T-reg marker) was the exclusive marker overexpressed in CRC-ECM-EVs. Comparing CRC-EVs and HC-EVs, we observed alteration of the cellular microenvironment and tumor phenotype markers, such as markers of angiogenesis (CD31), platelet activation (CD41b, CD42a, CD62p, CD142), immune response (CD11c, CD14, CD24, CD69, HLA-ABC, CD86) and epithelial-to-mesenchymal transition (ROR1, CD146). Interestingly, CD25 was also found to be significantly increased in CRC-EVs.

In conclusion, fresh biopsies contain EVs, exposing the heterogeneity of tumor and infiltrating cell composition, their interactions, and the resulting tumor state. At variance, EVs from decellularized tissues, entrapped in the extracellular matrix, may better embody the microenvironment alterations more prominent in the tumor surrounding tissue.

## 82. Wharton's jelly MSCS derived extracellular vesicles as a cell-free therapeutic approach for intervertebral disc degeneration

Veronica Tilotta^1^, Giuseppina Di Giacomo^1^, Claudia Cicione^1^, Luca Ambrosio^2^, Fabrizio Russo^2^, Rocco Papalia^2^, Gianluca Vadalà^2^, Vincenzo Denaro^2^

^1^Laboratory of Regenerative Orthopaedic, Research Unit of Orthopaedics and Traumatology, Campus Bio-Medico University, Rome, Italy.

^2^UOR of Orthopaedics and Traumatology, Campus Bio-Medico University Hospital Fondazione Rome, Rome, Italy.

**Introduction:** Intervertebral disc degeneration (IDD) affects more than 80% of the population. Current approaches to treat IDD are based on conservative or surgical procedures. Studies reported that paracrine factors such as extracellular vesicles (EVs) released by mesenchymal stRomel cells (MSCs) may regenerate IVD.

**Aim:** To explore the effects of Wharton’s Jelly MSC-derived EVs on human nucleus pulposus cells (hNPCs) under inflammatory stimulation within an *in vitro* 3D model of disc degeneration.

**Methods:** EVs were isolated by tangent filtration of WJ-supernatant and characterized by TEM, WB, NTA, and confocal microscopy to detect EV uptake in hNPCs. hNPCs were encapsulated in alginate beads and treated with growth medium, IL1β (10 ng/mL) and co-treated with IL1β and WJ-EVs at 10, 50, and 100 μg/ml. hNPCs proliferation, viability, nitrite production, extracellular matrix (ECM) synthesis, glycosaminoglycans (GAG) content, and the expression of phenotypic markers were analyzed.

**Results:** hNPCs proliferation was promoted by EVs 10 ug/mL at all time points. Live/dead suggested that the higher WJ-EVs dose was able to attenuate cell death compared to the IL1β group. Nitrite production was reduced at 10 μg/mL of WJ-EVs, also supported by gene expression levels of NOS2. A dose-dependent enhancement in GAG content was reported and confirmed by Alcian blue histological staining. Anabolic, catabolic, and inflammatory gene expression levels were positively modulated by WJ-EVs.

**Conclusions:** WJ-EVs ameliorate hNPCs growth, and attenuate ECM degradation and oxidative stress-related IDD progression. These findings offer new opportunities for the potential use of EVs as an alternative strategy for overcoming the side effects of cell therapy.

Acknowledgments: Financial support was received from the ilPSpine No 825925 and RESPINE No

732163 Horizon 2020 projects.

## 83. Autophagy beyond degradation: impairment of autophagic flux results in the release of extracellular vesicles carrying autophagy-associated markers

Giada Cerrotti^1^, Sandra Buratta^1,2,^ Raffaella Latella^1^, Roberto Maria Pellegrino^1^, Husam B.R. Alabed^1^, Brunella Tancini^1^, Paolo Gorello^1^, Francesco Arcioni^3^, Carla Emiliani^1,4^, Lorena Urbanelli^1,2^

^1^Department of Chemistry, Biology and Biotechnology, University of Perugia, Perugia, Italy.

^2^Extracellular Vesicles network (EV-net) of the University of Perugia, Perugia, Italy.

^3^Pediatric Onco-Hematology, Azienda Ospedaliera di Perugia, Perugia, Italy.

^4^CEMIN-Center of Excellence for Innovative Nanostructured Material, University of Perugia, Perugia 06132, Italy.

EVs have been considered an additional manner to contribute to disposing of extracellularly unnecessary material alternatively of digesting/recycling it via the autophagic/lysosomal pathway. Indeed, when the degradative capacity of the autophagic/lysosome pathway is impaired, extracellular disposal via EVs is increased. In this condition, there is an enhancement of autophagy-associated markers localized within EVs. We have characterized EV release under the condition of autophagic/lysosomal impairment induced by Bafilomycin A1 treatment in HEK-293 cells. Small EVs were isolated by density gradient ultracentrifugation, characterized by SEM and NTA, and their associated markers assessed by immunoblotting. Results showed a dose-dependent increase in the release of small EVs that were also enriched in the autophagy-associated markers LC3B. We then investigated whether EVs carrying autophagic markers could be retrieved in biological fluids. EVs were isolated from the plasma of healthy subjects by a combination of SEC and iodixanol density cushion, then blotted to detect the autophagosomal protein LC3B. Results showed that plasma contains LC3B-positive EVs that co-isolate with CD9-positive EVs. Consequently, we are developing a robust method to isolate and characterize EVs from blood with minimal contamination by plasma proteins, to assess whether EV-associated autophagic marker level may be altered in blood pathological conditions characterized by impairment of the lysosomal/autophagic pathway.

## 84. Differential regulation of PBMCs by plasma-derived extracellular vesicles from parkinson’s disease patients

Elena Vacchi^1,2^, Stefano Panella^3^, Carolina Balbi^3^, Lucio Barile^2,3^, Alain Kaelin-Lang^1,2,4,5^, Giorgia Melli^1,2,4^

^1^Laboratory for Biomedical Neurosciences, Neurocenter of Southern Switzerland, Ente Ospedaliero Cantonale, Lugano, Switzerland.

^2^Faculty of Biomedical Sciences, Università della Svizzera italiana, Lugano, Switzerland.

^3^Laboratory for Cardiovascular Theranostics, Cardiocentro Ticino Institute, Ente Ospedaliero Cantonale, Lugano, Switzerland.

^4^Neurology Department, Neurocenter of Southern Switzerland, Ente Ospedaliero Cantonale, Lugano, Switzerland.

^5^Department of Neurology, Inselspital, Bern University Hospital, University of Bern, Bern, Switzerland.

There is extensive and growing evidence about the role of inflammation in Parkinson’s disease (PD). In addition to neuroinflammatory processes occurring in the brain, elevated levels of pro-inflammatory factors are also present in the plasma of PD. Extracellular vesicles (EVs) carry important information on immunity and its dysregulations. We hypothesize that plasmatic inflammatory EVs may provide information about immune dysregulation in PD and have a functional role in disease initiation and progression.

Peripheral blood mononuclear cells (PBMCs) from healthy donors (*n* = 4) were incubated for 22 h with PD *vs.* HC circulating EVs previously labeled with the lipophilic dye DiR. For each PBMC subpopulation, we dissected the differential EV internalization and immune activation by FACS analysis. With a cytokine array on PBMC media treated with EVs from PD or HC, we evaluated the differential secretion of 105 cytokines. Finally, real-time PCR was performed for isolated PBMC subpopulations to analyze the production of specific cytokines.

PBMCs treated with EVs from PD showed increased activation of NKT cells. In this condition, PBMCs also release a higher amount of cytokines, particularly CXCL5, FGF7, IL18BPa, CCL3, CCL4, and CCL19. The real-time PCR analysis on isolated NKT cells, previously treated with EVs from PD, demonstrated the specific production of three out of six of these cytokines.

The differential response of PBMCs to plasma EVs derived from PD or HC suggests a differential message carried by EVs. A deep functional analysis may shed light on the role of inflammatory dysregulation in causing PD.

## 85. The role of extracellular vesicles in the modulation of temozolomide-resistant and sensitive glioblastoma cells

Diana Vardanyan^1^, Iulia Efimova^3,4^, Robin Demuynck^3,4^, Stefano Tacconi^2^, Dmitri Krysko^3^, Luciana Dini^1^

^1^Dept. of Biology and Biotechnologies Charles Darwin, the Sapienza University of Rome, Rome, Italy.

^2^CarMeN Laboratory Lyon-Sud Faculty of Medicine, University of Lyon, Lyon, France.

^3^Dept. Of Human Structure and Repair, Ghent University, Ghent, Belgium.

^4^Cancer Research Institute Ghent, Ghent, Belgium.

Glioblastoma (GBM) is a type of brain cancer that is highly aggressive and difficult to treat. It is characterized by rapid growth and invasion into surrounding brain tissue, making it challenging to completely remove with surgery or treat with chemotherapy or radiation therapy. In recent years, the use of extracellular vesicles (EVs) for diagnostic information, monitoring disease progression, and determining treatment efficacy in GBM has gained increasing attention. As a whole, this study is focused on determining the impact of temozolomide (TMZ) on the molecular profile of GBM cells-derived EVs and the influence of EVs on chemotherapy response, with the aim to understand the aggressive behavior and chemoresistance of GBM. Using shotgun liquid chRometography-tandem mass spectrometry proteomics, the molecular profiles of EVs isolated from TMZ-sensitive U87MG and resistant T98G cells were identified. Differentially expressed proteins associated with cell motility, migration, invasion, and cell death were found. Indeed, when non TMZ-treated sensitive U87MG and resistant T98G cells were co-incubated with EVs isolated from TMZ GBM cells, changes in migratory activity of cells, as detected by Wound Healing Assay, were observed. Thus, the therapeutic potential of the signaling of EVs in reducing chemoresistance and increasing TMZ sensitivity should be further investigated, as well as the possible development of new diagnostic and/or therapeutic approaches based on the proteome profile of isolated EVs.

## 86. Use of extracellular vesicles expressing sars-cov-2 spike protein as a model of virus-like particles for possible theragnostic applications

Roberta Verta^1^, Cristina Grange^2^, Sarah Tassinari^1^, Benedetta Bussolati^1^

^1^Department of Molecular Biotechnology and Health Sciences, University of Turin, Turin, Italy.

^2^Department of Medical Science, University of Turin, Turin, Italy.

Recently, we demonstrated the possibility of generating extracellular vesicles (EVs), expressing the SARS-CoV-2 spike protein on their surface (S-EVs), from HEK-293T-Spike transfected cells, as a model to study the virus and host cell interaction.

Spike protein is essential for the virus entrance in target cells, and it is composed of two different and functional subunits: S1 and S2. The S1 subunit binds angiotensin-converting enzyme 2 (ACE2) receptor on the host cells, while the S2 subunit is responsible for the virus-cell membranes fusion for the virus internalization. The aim of this work was to complete the previous study on S-EV characterization, analyzing the presence of different spike subunits on EV surface. Furthermore, we compared the S-EV uptake capability between ACE2 positive and negative cells in order to validate the specificity of S-ACE2 receptor interaction. Moreover, we observed that colchicine is able to decrease EVs entrance in target cells, independently of spike protein presence. To explore the potential role of colchicine in EV uptake inhibition, we isolated and characterized EVs from SKOV3 and HT29 cells. The SKOV3-EVs and HT29-EVs entrance were assessed in HMEC after colchicine treatment.

The MACSPlex analysis revealed different vesicle populations, in particular: S-EVs positive only for S1 (S1-EVs), S2 (S2-EVs), or both (S1S2-EVs) subunits. All the different subpopulations expressed tetraspanins (CD9, CD63, and CD81) and showed a homogenous surface markers pattern of expression. With super-resolution microscopy, we demonstrated the coexpression of CD63 with S1 or S2 on S-EVs (double or triple positive). In addition, this analysis reported an S-EVs dimension between 50 and 100 nm. Furthermore, the western blot analysis confirmed the expression of S1, S2, or the full-length protein (S1 and S2) in S-EV samples. Then, we investigated the interaction between DiI-labelled S-EVs and ACE2 positive or negative cells. The cytofluorimetric analysis showed that S-EVs were significantly more internalized by ACE2-positive cells compared to EVs without Spike (control EVs). In opposition, we observed a significant increase in the uptake of control EVs in ACE2-negative cells. Interestingly, we also demonstrated the inhibition of SKOV3-EV and HT29-EV entrance in HMEC in the presence of colchicine in comparison to untreated cells.

In conclusion, in our isolated S-EVs, we observed different subpopulations: S1-EVs, S2-EVs, and S1S2-EVs. The S-EVs subpopulations are simultaneously expressing CD9, CD63 and CD81. S-EVs represent a new theragnostic strategy for specific interaction (S1 subunit) and the fusogenic capability (S2 subunit). We showed that S-EVs bind to endothelial and bronchial epithelial cells through ACE2-dependent interaction, similar to the SARS-CoV-2 virus. Moreover, colchicine decreased virus-like particles and tumor EV uptake due to its effect on microtubules. Colchicine seems to be a new possible therapeutic solution to inhibit EV entrance in pathological conditions.

## 87. Brain-derived extracellular vesicles in dementia

Caterina Visconte^1^, Maria Serpente^2^, Maria Teresa Golia^3^, Claudia Verderio^3^, Martina Gabrielli^3^, Federica Sorrentino^1^, Marina Arcaro^2^, Andrea Arighi^2^, Beatrice Arosio^1^, Elio Scarpini^2^, Daniela Galimberti^1,2^, Chiara Fenoglio^1^

^1^University of Milan, Milan, Italy.

^2^Neurodegenerative Diseases Unit, Fondazione IRCCS Ca' Granda, Ospedale Maggiore Policlinico, Milan, Italy.

^3^CNR Institute of Neuroscience, Milan, Italy.

The aim of the present study was to examine the changes in Extracellular Vesicle (EV) content and concentration in the context of dementia, by comparing total EVs and neural and microglial derived EVs (NDVs and MDVs), isolated from plasma of aging controls, Alzheimer’s Disease (AD) and Mild Cognitive Impairment (MCI) patients.

Total EVs were isolated from the plasma of eighty subjects recruited by using ExoQuickULTRA Exosome Precipitation Solution (SBI). Subsequently, total EVs were enriched for neural and microglial sources by immunoprecipitation with anti-L1CAM and anti-TMEM119 antibodies, respectively. Total EVs and subpopulations were characterized through Nanosight Nanoparticle Tracking Analysis (NTA) and Western Blotting. A panel of 754 miRNAs was simultaneously detected by RT-qPCR using TaqMan OpenArray technology in a QuantStudio 12K System (ThermoFisher Scientific). In addition, the V-Plex Human Neuroinflammation Panel was applied to measure cytokines and chemokines using a Meso QuickPlex 120 system.

Results showed an alteration in NDVs and MDVs release during cognitive decline, with MDVs displaying the highest neurotoxic effect. Specific miRNA levels were found to be deregulated according to the EV subpopulations, and they were found to be associated with common and specific biological pathways. Importantly, we also observed a pattern of inflammatory molecules augmented in MDVs from AD patients.

Our study contributes to clarifying the content of specific brain-derived EVs in the context of AD pathology. In particular, these results demonstrated an alteration in NDV and MDV release during cognitive decline, providing remarkable insight into the role of EV content in neurodegeneration.

## 88. Functional effects of plasma-derived extracellular vesicles of patients with parkinsons disease *in vitro* can reveal novel pathways of neurodegeneration

Ankush Yadav^1,2^, Elena Vacchi^1,2^, Sandra Pinton^1,2,3^, Alain Kaelin-Lang^1,2,3,4^, Giorgia Melli^1,2,3^

^1^Laboratories for translational Research, Neurocenter of Southern Switzerland, Ente Osedaliero Cantonale, Bellinzona, Switzerland.

^2^Faculty of Biomedical Sciences, Università della Svizzera Italiana, Lugano, Switerland.

^3^Neurology Department, Neurocenter of Southern Switzerland, Ente Ospedaliero Cantonale, Lugano, Switzerland.

^4^Neurology Department, Inselspital, Bern University Hospital, University of Bern, Bern, Switzerland.

**Objective:** Extracellular vesicles (EVs) are secreted membrane particles in cell-to-cell communication. Cerebrospinal fluid (CSF) and plasma EVs may mediate the spread of neuroinflammation and pathological proteins in Parkinson`s disease (PD) and other neurodegenerative diseases. We aim to analyze the functional role of plasma EVs as pathways/biomarkers, exploring their potential as targets for novel therapeutic approaches.

**Methods:** EVs were isolated from the plasma of PD and sex and age-matched healthy control (HC) by size exclusion chRometography using IZON qEV. By nanoparticle tracking analysis (NTA), we determined the EV concentration and size. The effect of plasma EVs on a neuronal cell line (differentiated SHSY-5Y) was determined by apoptosis assay and axonal length measurement using immune-fluorescence and ImageJ software applications. The impact of EVs from PD Vs. HC on a human glial cell line (HMC3) was also analyzed. Cytokine expression in human HMC3 cells treated with EVs from PD and HC was investigated using Human Neuro Discovery Cytokine Array.

**Result:** The average axonal length was significantly reduced when neurons were treated with plasma EVs from PD for 24 h and 48 h compared to HC. When treated with PD-derived EVs for 24 h and 48 h, the percentage of apoptotic cells gradually increased compared to HC-derived EVs. Pro-inflammatory cytokines such as CRP, IFN-gamma, TNF-alpha, and IL-6 Beta were higher in HMC3 treated with PD-derived EVs compared to HC-derived EVs.

**Conclusion:** These preliminary results suggest that plasma EVs from PD may have a direct and indirect (mediated by glia cells) toxic effect on neurons and influence the initiation and progression of the disease.

